# H3K27me3 demethylases alter *HSP22* and *HSP17.6C* expression in response to recurring heat in *Arabidopsis*

**DOI:** 10.1038/s41467-021-23766-w

**Published:** 2021-06-09

**Authors:** Nobutoshi Yamaguchi, Satoshi Matsubara, Kaori Yoshimizu, Motohide Seki, Kouta Hamada, Mari Kamitani, Yuko Kurita, Yasuyuki Nomura, Kota Nagashima, Soichi Inagaki, Takamasa Suzuki, Eng-Seng Gan, Taiko To, Tetsuji Kakutani, Atsushi J. Nagano, Akiko Satake, Toshiro Ito

**Affiliations:** 1 Division of Biological Science, Graduate School of Science and Technology, Nara Institute of Science and Technology, Ikoma-shi, Nara Japan; 2 Precursory Research for Embryonic Science and Technology, Japan Science and Technology Agency, Kawaguchi-shi, Saitama Japan; 3 Faculty of Design, Kyusyu University, Minami-ku, Fukuoka Japan; 4 Department of Biology, Faculty of Science, Kyusyu University, Nishi-ku, Fukuoka Japan; 5 Faculty of Agriculture, Ryukoku University, Otsu-shi, Shiga Japan; 6 Department of Biological Sciences, Graduate School of Science, University of Tokyo, Bunkyo-ku, Tokyo, Japan; 7 Department of Biological Chemistry, College of Bioscience and Biotechnology, Chubu University, Kasugai-shi, Aichi Japan; 8 Temasek Life Sciences Laboratory, 1 Research Link, National University of Singapore, Singapore, Republic of Singapore; 9 CREST, Japan Science and Technology Agency, Kawaguchi-shi, Saitama Japan; 10 National Institute of Genetics, Mishima-shi, Shizuoka, Japan

**Keywords:** Gene silencing, Plant molecular biology, Heat

## Abstract

Acclimation to high temperature increases plants’ tolerance of subsequent lethal high temperatures. Although epigenetic regulation of plant gene expression is well studied, how plants maintain a memory of environmental changes over time remains unclear. Here, we show that JUMONJI (JMJ) proteins, demethylases involved in histone H3 lysine 27 trimethylation (H3K27me3), are necessary for *Arabidopsis thaliana* heat acclimation. Acclimation induces sustained H3K27me3 demethylation at *HEAT SHOCK PROTEIN22* (*HSP22*) and *HSP17.6C* loci by JMJs, poising the *HSP* genes for subsequent activation. Upon sensing heat after a 3-day interval, JMJs directly reactivate these *HSP* genes. Finally, *jmj* mutants fail to maintain heat memory under fluctuating field temperature conditions. Our findings of an epigenetic memory mechanism involving histone demethylases may have implications for environmental adaptation of field plants.

## Abstract (web summary)

Acclimation to high temperature increases tolerance of heat shock in plants. Here the authors show that JUMONJI H3K27me3 demethylases are needed for heat acclimation in Arabidopsis and act at loci encoding HEAT SHOCK PROTEINS to facilitate induction upon heat stress.

## introduction

The ability to adapt to environmental changes is essential for the survival of plants, which are sessile organisms^1^. Plants “remember” exposure to heat over several days, which affects their responsiveness to subsequent heat exposures^2^. Exposure to moderately elevated temperatures enables plants to acquire thermotolerance to subsequent lethal high temperatures^3^: this phenomenon is known as acquired thermotolerance or heat acclimation. Although epigenetic regulation of plant gene expression is well studied^4–8^, how plants maintain heat memory over time remains unclear. In *Arabidopsis thaliana*, HEAT SHOCK TRANSCRIPTION FACTOR A2 (HSFA2) is necessary for the maintenance of acquired thermotolerance, while HSFA1s play important roles in basal thermotolerance^9–12^. *HEAT SHOCK PROTEIN* (*HSP*) genes encode molecular chaperones that protect cellular proteins from denaturation. When high temperature is sensed, HSFA2 levels rise. The transient binding of HSFA2 to *HSP* genes causes a sustained increase in histone marks, such as histone H3 lysine 4 trimethylation (H3K4me3), and the expression of *HSP* genes^13–15^. After heat exposure, HSFA2 levels decline gradually, while H3K4me3 levels and *HSP* expression remain high. Thus, repressive histone marks that downregulate *HSP* genes^15^ also play an important role in the maintenance of appropriate levels of *HSP* expression. Despite the importance of histone modification enzymes, little is known about the underlying mechanisms by which these enzymes regulate flexible and reversible *HSP* gene expression.

JUMONJI (JMJ) proteins^4–8^ are evolutionarily conserved demethylases that regulate diverse biological processes. Previous studies in *A. thaliana* revealed the link between a specific subset of histone H3 lysine 27 trimethylation (H3K27me3) demethylases and the heat response. *JMJ30* mRNA and JMJ30 protein are stabilized by constant warm temperature (29 °C for 21 days). *JMJ12/RELATIVE OF EARLY FLOWERING* (*REF6*) expression is directly upregulated by HSFA2 under prolonged high temperature conditions^16^ (30 °C for 13 days).

In this study, we investigated the role of H3K27me3 demethylases in acquired thermotolerance in response to recurring heat exposures separated by short intervals. We demonstrated that JMJ proteins maintain low levels of repressive histone marks on small chaperone-encoding *HSP*s^13,14^ that function as memory genes. Using inducible JMJs and mutants of small *HSP*s, we demonstrated that the underlying basis of heat memory is at least partially mediated by the sustained demethylation of H3K27me3 on small *HSPs*. We developed a mathematical stochastic model for this histone modification-based transcriptional memory that successfully predicts expression levels of *HSP*s. Moreover, our data under recapitulated fluctuating temperature conditions indicates that JMJ-mediated sustained H3K27me3 demethylation on small *HSPs* controls recurring heat memory.

## Results

### Heat acclimation defects in H3K27me3 demethylase mutants

**Fig. 1:**
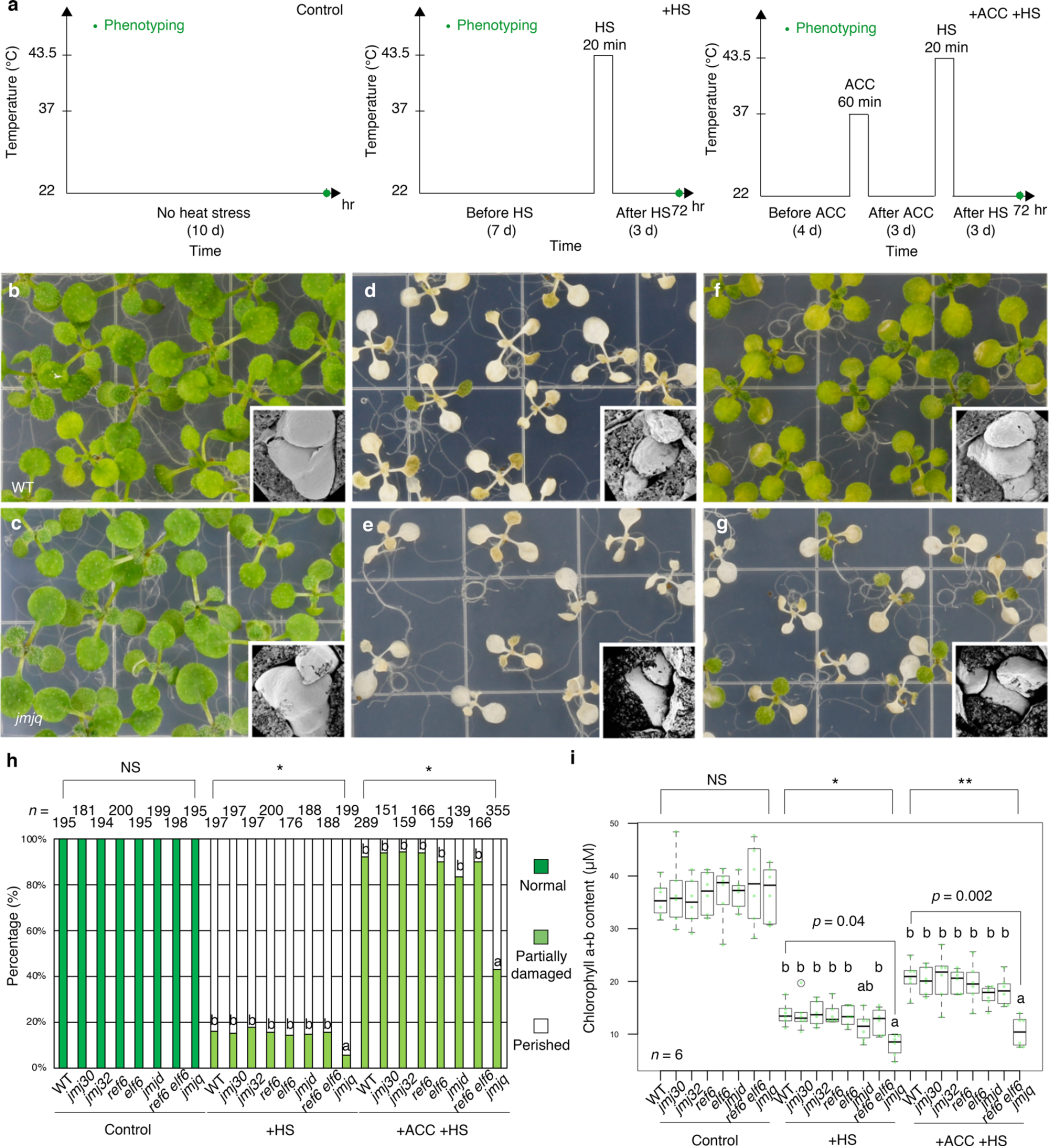
H3K27me3 demethylase activity is required for acquired thermotolerance. **a** Schematic representation of the temperature conditions used in this study. The time of phenotyping is indicated with a green dot. Left, normal plant growth conditions. Center, basal thermotolerance conditions (+HS). Right, heat-stress memory conditions (+ACC +HS). **b**, **c** Wild type (**b**) and *jmj30 jmj32 ref6 elf6* quadruple mutants (*jmjq*) (**c**) grown under control conditions. **d**, **e** Wild type (**d**) and *jmjq* (**e**) grown under +HS conditions. **f**, **g** Wild type (**f**) and *jmjq* (**g**) grown under +ACC +HS conditions. Insets show scanning electron microscope images of representative shoot apical meristems. **h** Quantification of survival rate. In all, 10-day-old seedlings grown under the three different temperature conditions were categorized into three groups based on phenotypic severity: green, normal growth; light green, partially damaged; and white, perished. Different letters indicate significant differences. Significance was determined by the *χ*^2^ test followed by a post-hoc test. *n* > 138. **i** Quantification of chlorophyll contents. Sample minimum (lower bar); lower quartile (box); median (middle line); upper quartile (box); and sample maximum (upper bar). Light green dots and white circles represent the chlorophyll contents of individual samples and statistical outliers, respectively. One-way ANOVA test, *p < 1.0 × 10^–3^; **p < 1.0 × 10^–4^. Different letters indicate significant differences based on post-hoc Tukey’s HSD test. *p*-values based on post-hoc Tukey’s HSD test between wild type and *jmjq* mutants are shown. *p* < 0.05. NS nonsignificant. *n* = 6.

To gain insight into the role of histone modification enzymes in the maintenance of heat memory, we focused on a group of the Jumonji-C-domain-containing proteins (JMJs) involved in H3K27me3 removal. Among the 21 JMJ proteins in *A. thaliana*, five reportedly possess H3K27me3 demethylation activity: JMJ30, JMJ32, JMJ11/EARLY FLOWERING 6 (ELF6), REF6, and JMJ13^4–8^. To see whether these proteins are involved in heat memory, we investigated the heat responses of a series of *jmj* mutants (Fig. 1a–i). In addition to the single mutants *jmj30-2*, *jmj32-1*, *elf6-3*, *ref6-1*, and *jmj13G*, we also looked at a *jmj30-2 jmj32-1* double mutant (*jmjd*) and a *jmj30-2 jmj32-1 elf6-3 ref6-1* quadruple mutant (*jmjq*). At normal growth temperatures, we observed no difference in leaf size between 10-day-old wild-type and *jmjd* and *jmjq* seedlings (Supplementary Fig. 1). We used a weak mutant allele of *elf6* (*elf6-3*) for the quadruple mutant because having no differences in leaf size at this developmental stage^8^ would allow us to address the role of H3K27me3 demethylases in heat memory without interference from secondary effects due to morphological changes. When grown at 22 °C (the control condition), the wild type and all mutant plants, including *jmj30*, *jmjd*, and *jmjq*, survived normally (Fig. 1a–c, h, i). Exposure to heat shock at 44 °C (+HS) led to a significant reduction in survival rates in both wild-type and mutant plants (Fig. 1d, e, h, i). Among seedlings that had been acclimated by prior exposure to a sub-lethal heat treatment (ACC), ~80% of the wild type and all mutants except *jmjq* survived (Fig. 1f–i), and they had similar a chlorophyll content and fresh weight (Fig. 1f–i and Supplementary Fig. 2). In the wild type, the acclimation effect lasted for at least 4 days (Supplementary Fig. 3). We also generated a *jmj30-2 jmj32-1 jmj13G*^8^ triple mutant, which did not show any defects in heat acclimation phenotypes (Supplementary Fig. 4). The *jmjq* mutant seedlings showed lower HS survival, indicating that these four JMJ demethylases are involved in the primary heat response. Furthermore, *jmjq* mutants showed a significantly lower survival rate, chlorophyll content, and fresh weight after acclimation and heat shock (+ACC +HS) than the wild type, *jmj30*, *jmjd*, or any other mutant combination (Fig. 1f–i and Supplementary Fig. 2). Although *jmjq* mutants had defects in both basal and acquired thermotolerance, the effect on acquired thermotolerance was greater than that on basal thermotolerance based on the *p*-values (Fig. 1i and Supplementary Fig. 2). Thus, our results suggested that JMJ30, JMJ32, ELF6, and REF6 are redundantly required for heat acclimation at least in part.

### H3K27me3 demethylases prime *HSP22* and *HSP17.6C* activation

**Fig. 2:**
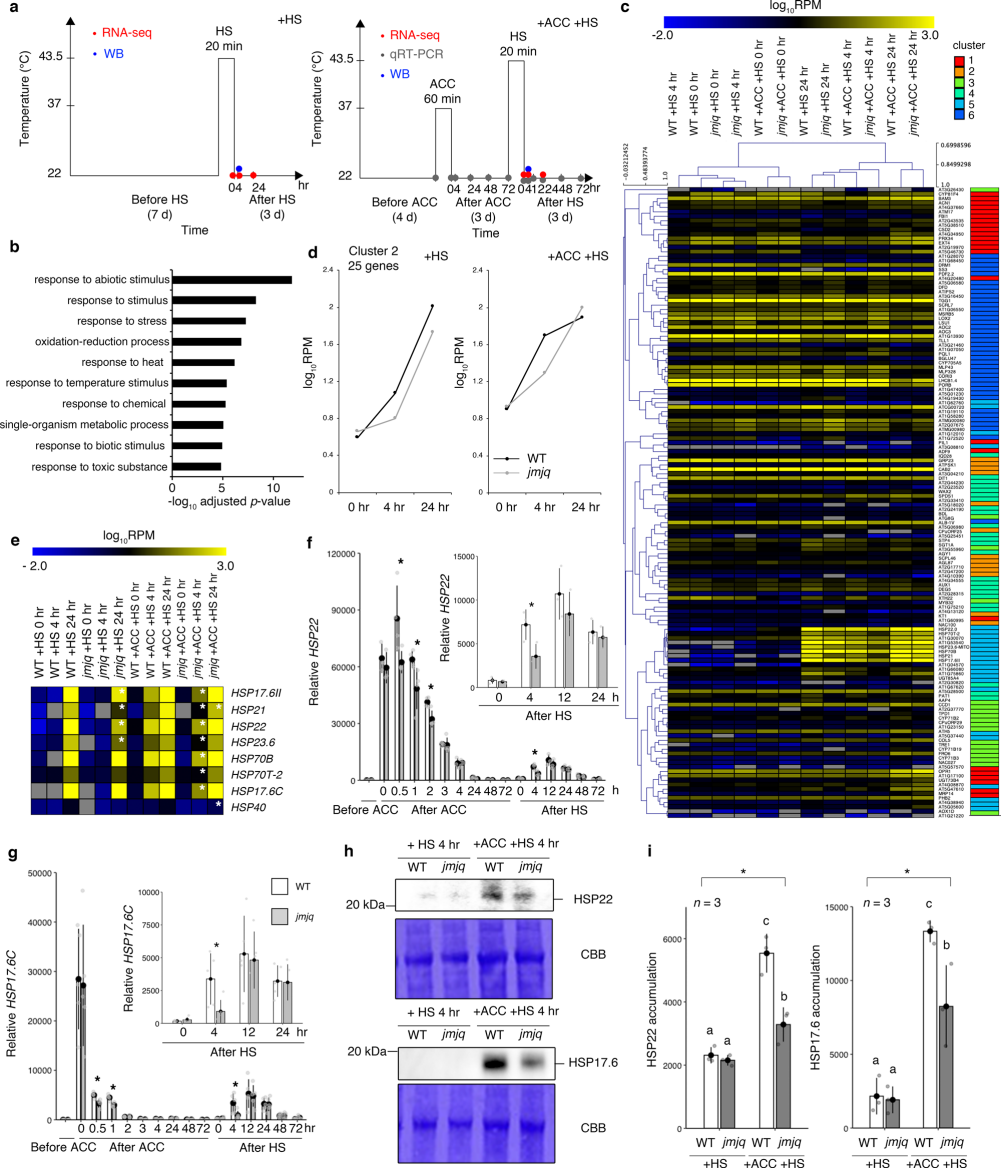
Histone H3K27me3 demethylases mediate rapid reactivation of *HSP* genes during heat acclimation. **a** Schematic representation of the temperature conditions used. Left, basal thermotolerance condition (+HS); right, heat-stress memory condition (+ACC +HS). Colored dots indicate the times of different assays: RNA-seq, red; qRT-PCR, gray; immunoblotting, blue. **b** GO term enrichment analysis of 142 differentially expressed genes. The top 10 terms determined by their –log_10_-adjusted *p*-values based on two-tailed *z*-test are shown. **c** A *k*-means clustering of genes differentially expressed between acclimatized wild type and *jmjq* mutants after 0, 4, or 24 h of a tester heat stress. The FDR was <0.05. **d** Gene expression over time in cluster 2 under basal thermotolerance (left) and heat-stress memory (right) conditions. **e** Heatmap of expression of the *HSP* genes in cluster 2. White asterisks indicate significant differences between the wild type (WT) and *jmjq* grown under the same conditions. FDR < 0.05. **f**, **g** qRT-PCR verification of *HSP22* (**f**) and *HSP17.6C* (**g**) transcript levels in the wild type and *jmjq* mutants grown under the condition shown in Fig. 2a (right). Gray dots represent the expression levels of individual samples. Asterisks indicate significant differences (*p* < 0.05) between the wild type and *jmjq* at the same time point based on a two-tailed Student’s *t* test. **h**, **i** Immunoblotting (WB) of HSP22 and HSP17.6 in the wild type and *jmjq* grown under conditions shown in Fig. 2a. **h** WB analysis using the HSP22 (above) and HSP17.6 (below) antibodies to probe protein extracts. Coomassie brilliant blue-stained membranes (CBB) are shown as loading controls. **i** Quantification of WB signals. One-way ANOVA test; *p < 0.05. Different letters indicate significant differences based on a post-hoc Tukey’s HSD test (*p* < 0.05). NS nonsignificant. *n* = 3.

Next, we conducted RNA-seq to identify JMJ-dependent targets whose expression changed in response to acclimation and heat shock (Fig. 2a and Supplementary Fig. 5). In general, plants acclimatized to a stress often respond to the triggering stress faster, earlier, and/or more strongly than non-acclimatized plants^2^. Thus, we screened for genes that showed no difference in expression immediately after HS in acclimatized wild-type and *jmjq* mutant seedlings, but showed significant differences after either 4 or 24 h of HS (Fig. 2a). These analyses identified 142 genes that were differentially expressed in the *jmjq* mutant compared to the wild type (FDR < 0.05) (Fig. 2b, c and Supplementary Data 1 and 2). As expected, stress-related Gene Ontology (GO) terms such as “response to stress” and “response to heat” were significantly enriched among these genes (Fig. 2b and Supplementary Data 3). Figure 2c shows a clustering heatmap of the expression changes observed for these 142 genes in all datasets. To determine which of the 142 candidate genes are up- or downregulated during heat acclimation, we used k-means clustering. An optimal number of six clusters were identified using Pearson correlation based on levels of gene expression observed at 0 h, 4 h, and 24 h (Fig. 2c and Supplementary Fig. 6). In all, 62 genes were upregulated and 80 were downregulated in *jmjq* relative to the wild type. Two clusters (1 and 6; 62 genes) comprised genes upregulated in the *jmjq* mutant. Four clusters (2, 3, 4, and 5; 80 genes) comprised genes downregulated in the *jmjq* mutant (Supplementary Fig. 6). Cluster 2 (25 genes) showed a rapid increase in expression after HS in acclimatized wild-type seedlings (Fig. 2d and Supplementary Fig. 6), but this induction was delayed in acclimatized *jmjq* mutants (Fig. 2d). This cluster included eight differentially expressed *HSP* genes whose products function as molecular chaperones (Fig. 2d, e and Supplementary Data 2). Some of these genes were differentially expressed in *jmjq* mutants grown under HS conditions, but only at later time points (Fig. 2d, e).

Among the cluster 2 *HSP* genes, the small *HSP* genes *HSP22*, *HSP17.6C*, and *HSP21* are known for their importance in heat acclimation^17,18^. To explore the expression dynamics of these small *HSP* genes during heat acclimation, we performed qRT-PCR. In the acclimatized wild-type plants, *HSP22*, *HSP17.6C*, and *HSP21* were rapidly induced to high levels after exposure to 20-min ACC at 37 °C is complete (hereinafter referred to as “0 h after ACC”), and were moderately activated within 4 h of HS (Fig. 2f, g and Supplementary Figs. 7 and 8). This relatively moderate and delayed activation of *HSP* genes upon HS might reflect the time needed to recover from cell damage caused by the HS^19^ (Supplementary Fig. 9). The expression of *HSP22*, *HSP17.6C*, and *HSP21* following ACC and after 4 h of HS was significantly lower in acclimatized *jmjq* mutants than in acclimatized wild-type seedlings (Fig. 2f, g and Supplementary Figs. 7 and 8). Similar expression patterns of *HSP22* gene were observed in heat acclimation-defective mutants, such as *hsfa2*^9^ (Supplementary Fig. 10). In addition, basal thermotolerance-defective *hsfa1a/b/d*^11^ mutants did not show upregulation of *HSP* genes upon either ACC or HS (Supplementary Fig. 10). The similar *HSP* expression patterns of the *jmjq* and *hsfa2* mutants support our idea that JMJ demethylases contribute more to heat acclimation than to basal thermotolerance. This is also consistent with our phenotypic observations (Fig. 1) and transcriptome analysis (Fig. 2a–e). Furthermore, the accumulation of HSP22, HSP17.6, and HSP21 proteins was lower in acclimatized *jmjq* mutants compared to that in the wild type 4 h after HS^20,21^ (Fig. 2h, i and Supplementary Figs. 11 and 12). These data indicate that JMJs play an important role in the rapid induction of small *HSP* genes.

### Sustained H3K27me3 demethylation and H3K4me3 methylation alter *HSP22* and *HSP17.6C* induction

**Fig. 3:**
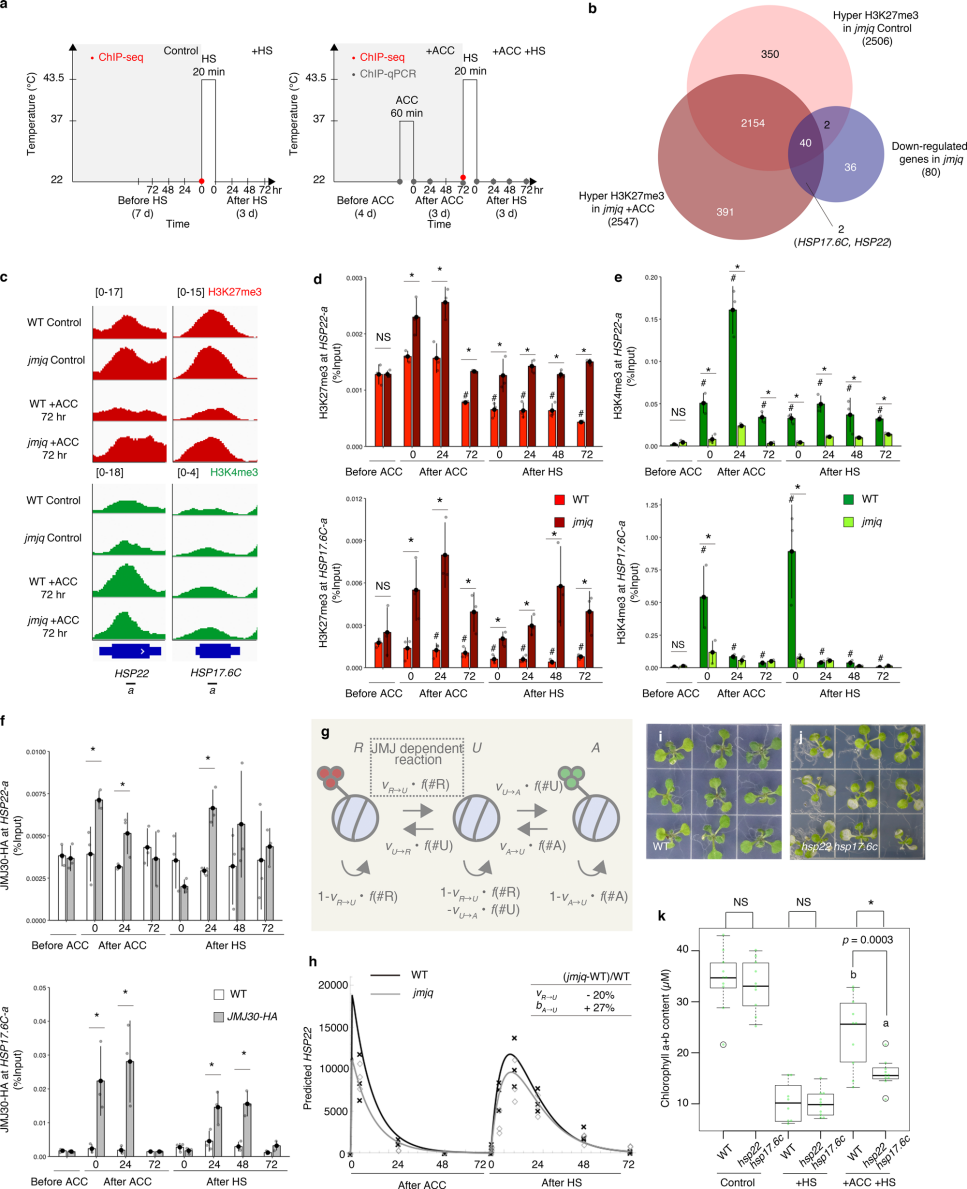
JUMONJI demethylases remove H3K27me3 histone modifications from key heat-memory genes in response to heat. **a** Schematic representation of the temperature conditions used. Colored dots indicate the times of different assays: ChIP-seq, red; ChIP-qPCR, gray. Left, basal thermotolerance condition (+HS); right, heat-stress memory condition (+ACC +HS). **b** Venn diagram showing the overlaps between genes downregulated in *jmjq* mutants and genes with elevated H3K27me3 in the mutant, with and without acclimation (*p* = 6.2 × 10^–26^ for elevated H3K27me3). **c** H3K27me3 levels by ChIP-seq along *HSP* gene regions in the wild type and *jmjq*, without and with acclimation. *HSP22-a* and *HSP17.6C-a* indicate the ChIP amplicons used in the ChIP assays. **d**–**f** H3K27me3 (**d**) and H3K4me3 (**e**) levels in the wild type and *jmjq*, and JMJ30-HA (**f**) levels in the wild type and *jmjq*, as determined by ChIP-qPCR. Gray dots represent the levels of individual samples. A two-tailed Student’s *t* test compared to the wild type before ACC; ^#^*p* < 0.05. A two-tailed Student’s *t* test comparing wild type and *jmjq* mutants at the same time point; *p < 0.05. NS not significant. **g** Diagram of the mathematical model describing the histone modification process. **h** The profile of *HSP22* expression in the wild type and *jmjq* predicted by the model under the conditions shown in Fig. 3a. Experimental data from the wild type (crosses) and *jmjq* (diamonds) are also plotted. **i**, **j** Wild type (**i**) and *hsp22 hsp17.6c* mutants (**j**) grown under +ACC +HS conditions. **k** Quantification of chlorophyll contents. Sample minimum (lower bar); lower quartile (box); median (middle line); upper quartile (box); and sample maximum (upper bar). One-way ANOVA test; *p < 0.05. Different letters indicate significant differences based on a post-hoc Tukey’s HSD test (*p* < 0.05). NS nonsignificant. *n* > 7.

We hypothesized that acclimation-dependent rapid *HSP* activation mediated by JMJs was associated with sustained demethylation of H3K27me3. Based on our observation that the acclimatized wild-type plants remembered heat exposure for at least 3 days, we harvested samples from wild-type and *jmjq* mutant plants 3 days after ACC and from equivalent non-acclimatized controls and analyzed them by chromatin immunoprecipitation followed by sequencing (ChIP-seq) (Fig. 3a–c). In agreement with a role for JMJs in H3K27me3 removal, >2500 genes were H3K27me3 hypertrimethylated in the gene body in *jmjq* compared to the wild type, irrespective of whether the plants were non-acclimatized or acclimated (Fig. 3b and Supplementary Figs. 13–15). For each condition, 55% of genes identified by RNA-seq as being downregulated in *jmjq* also showed H3K27me3 hypermethylation (Fig. 3b and Supplementary Data 4). An overlap that was significantly greater than that expected by chance (*p* = 6.2 × 10^−26^). Among eight *HSP* genes that were downregulated in *jmjq* mutants, the *HSP22* and *HSP17.6C* loci showed acclimation-dependent removal of H3K27me3 by JMJs (Fig. 3b, c and Supplementary Data 4 and 5). Thus, we mainly focused on these two genes for further analysis.

To understand the histone modification dynamics of these small *HSP* genes during heat acclimation, ChIP followed by qPCR (ChIP-qPCR) was conducted. In the wild type, H3K27me3 levels at the *HSP22* and *HSP17.6C* loci in the gene body (*HSP22-a* and *HSP17.6C-a*, respectively) reduced gradually and kept lower following ACC and HS (Fig. 3c, d (#)). The gradual reduction of H3K27me3 levels at the *HSP22-a* and *HSP17.6C-a* between ACC and HS was not observed without ACC (Supplementary Fig. 16). In the *jmjq* mutant, however, the H3K27me3 levels after acclimation were significantly higher than those in wild type (Fig. 3d (*) and Supplementary Fig. 16). This could be due to failure to active removal of H3K27me3 and/or prevention of H3K27me3 spreading (see Discussion section). No difference in H3K27me3 was observed between the wild type and *jmjq* mutants under the HS condition (Supplementary Fig. 16). In addition, these differences were specific to *HSP22* and *HSP17.6C*, since H3K27me3 levels in the *TA3* (transposable element) negative control were lower overall than those in *HSP22-a* and *HSP17.6C-a* regardless of genotype (Fig. 3d and Supplementary Fig. 17). Furthermore, the *jmjq* mutant had higher H3K27me3 levels at the *HSP22-a* and *HSP17.6C-a* than *jmj30-2 elf6-3 ref6-1* and *jmj32-1 elf6-3 ref6-1* (Supplementary Fig. 18), suggesting that H3K27me3 levels are also regulated by functional redundancy. There was no difference between wild type and *jmjq* mutants in histone H3 signals at these two loci following ACC and HS (Supplementary Fig. 19), suggesting that it is histone modifications, rather than the locations of histones (or nucleosomes) along the DNA that are affected by acclimation. Hence, JMJs such as JMJ30 and JMJ32 mediate H3K27me3 removal at *HSP22* and *HSP17.6C* in response to ACC at least in part.

The delicate balance between H3K27me3 and H3K4me3 results in a poised transcriptional state^15,22^. The levels of the permissive H3K4me3 histone mark at *HSP22-a* and *HSP17.6C-a* increased after ACC and HS in wild type, but each gene shows different histone modification patterns (Fig. 3e and Supplementary Data 5 and 6). In the case of H3K4me3 at *HSP22-a*, the first significant differences between wild type and *jmjq* were seen just after acclimation (0 h after ACC). The *HSP22* locus is decorated with H3K4me3 during the phase between ACC and HS, as reported previously^14^. Consistent with the high levels of H3K27me3 at *HSP22-a* in *jmjq*, H3K4me3 deposition was greatly attenuated in the mutant (see Discussion). On the other hand, H3K4me3 deposition at the *HSP17.6C-a* locus in wild type was observed just after ACC and after HS and this deposition was not observed in *jmjq*, suggesting that other permissive histone modification(s) might antagonize H3K27me3 at *HSP17.6C* to switch on the gene. Furthermore, H3K4me3 levels in the *TA3* negative control locus were overall lower than those in the *HSP22-a* locus regardless of genotype (Supplementary Fig. 17). Thus, JMJs mediate the balance between H3K27me3 and H3K4me3 at *HSP22*.

We also performed the ChIP assay using the previously described biologically functional JMJ30-HA tagged line^4^ in the *jmj30* mutant background. This rescued line had *JMJ30* expression levels similar to those of the wild type (Supplementary Fig. 20). Consistent with the levels of H3K27me3 and gene expression of *HSP22* and *HSP17.6C*, JMJ30 directly bound to these genes in response to ACC and HS (Fig. 3f), whereas JMJ30 binding was not observed at the *TA3* locus (Supplementary Fig. 20). The highest JMJ30 binding peaks were observed in the gene bodies of *HSP22-a* and *HSP17.6C-a* (Supplementary Fig. 20), suggesting that the distribution of JMJ30-HA overlaps with that of the changes in histone modification over the length of these regions. REF6 binding peaks at these two loci were also examined. Two independent public REF6 ChIP-seq data sets^7,23^ showed no REF6 binding at *HSP22* or *HSP17.6C* (Supplementary Fig. 21). Furthermore, REF6 did not bind to these two genes in response to ACC and HS based on ChIP-qPCR. Taken together, our results suggest that JMJ30 directly removes H3K27me3 at *HSP22* and *HSP17.6C* in response to heat.

To formulate the dynamics of H3K27me3 and H3K4me3 histone modification by JMJs, we established a mathematical model using the *HSP22* data^24,25^ (Fig. 3g). Recovery from HS was considered to explain the gradual upregulation of *HSP22* after HS in acclimatized wild type and *jmjq* mutants (Fig. 2f and Supplementary Fig. 9). On the basis of our calculations from the model, *HSP22* expression is immediately induced upon ACC and gradually induced after HS (Fig. 3h). Furthermore, the model predicted that *HSP22* expression after HS would be lower in acclimatized *jmjq* mutants than in acclimatized wild type plants (Fig. 3h). Consistent with the biochemical function of JMJs, a comparison of the best-fit parameters of the wild type and the *jmjq* mutant indicated that the *jmjq* mutant transitioned from the repressively modified to the derepressed, unmodified state (${v}_{{\rm{R}}\to {\rm{U}}}$) at a lower rate (Fig. 3h and Supplementary Note 1). The model highlighted the importance of the presence of multiple modified nucleosomes for lengthening the duration of the memory effect (Supplementary Fig. 22).

To address the functions of *HSP22* and *HSP17.6C* during heat acclimation, double mutants were generated. Genetic analysis revealed that *hsp22*^18^ *hsp17.6c* double mutants exhibited reduced heat-acclimation ability in terms of chlorophyll content and fresh weight, although *hsp17.6c* could be a weak allele (Fig. 3i–k and Supplementary Fig. 23). Unlike the *jmjq* mutants, acclimatized *hsp22 hsp17.6c* double mutants had the same survival rate as acclimatized wild-type plants (Supplementary Fig. 23). Because the heat-acclimation defects in *hsp22 hsp17.6c* double mutants were less severe than those in *jmjq* mutants, the conferral of gene memory by these two genes accounts for only a part of the phenotypic effect of *jmjq* mutants. Ectopic expression of *HSP17.6C* in the *jmjq* mutant led to increased heat tolerance (Supplementary Fig. 24), similar to the effect of *HSP22* overexpression^18^. These results indicated that *HSP22* and *HSP17.6C* function to confer heat tolerance.

### JMJ30 induction prior to acclimation results in sustained H3K27me3 demethylation

**Fig. 4:**
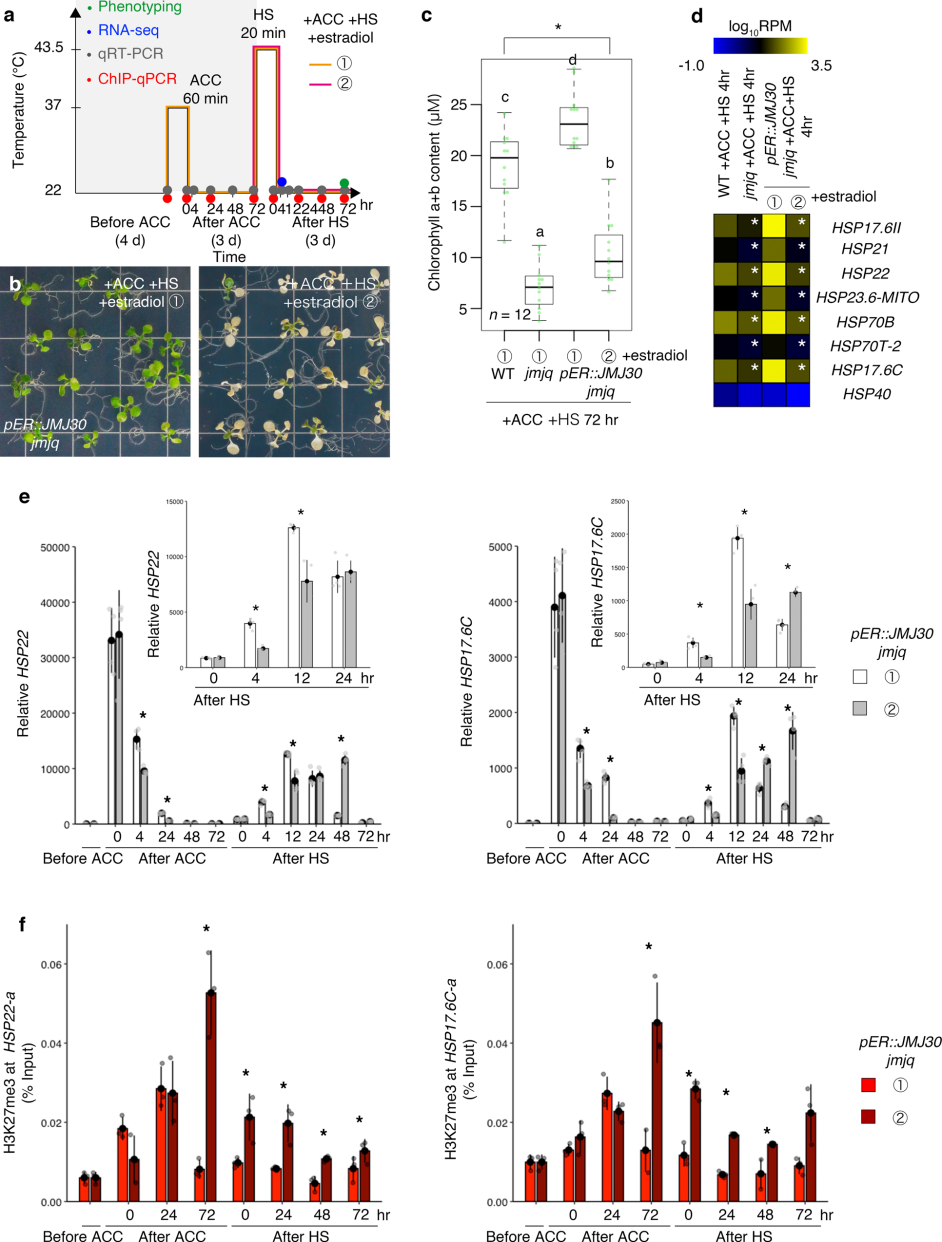
JUMONJI demethylases are required for the removal of H3K27me3 histone modifications from heat-memory genes before acclimation. **a** Schematic representation of heat-stress memory conditions. Colored dots indicate the times of different assays: phenotyping, green; RNA-seq, blue; qRT-PCR, gray; and ChIP-qPCR, red. Orange and magenta lines show the two different time-courses of β-estradiol application. **b** *pER8::JMJ30* transgenic plants in the *jmjq* mutant background subjected to β-estradiol application before acclimation (1, left) and before heat shock (2, right). Plants were grown under the heat-stress memory condition. **c** Quantification of chlorophyll contents of plants shown in Fig. 4b. Sample minimum (lower bar); lower quartile (box); median (middle line); upper quartile (box); and sample maximum (upper bar). One-way ANOVA test; *p < 0.05. Different letters above bars indicate significant differences based on a post-hoc Tukey HSD test (*p* < 0.05). *n* = 12. **d** Expression heatmap of the *HSP* genes downregulated in *jmjq*, determined by RNA-seq. White asterisks indicate significant differences between WT and *jmjq* grown under the same conditions or between *pER8::JMJ30* transgenic plants in the *jmjq* mutant background subjected to β-estradiol application before acclimation or before heat shock. The FDR was <0.05. **e**, **f** Gene expression levels (**e**) and H3K27me3 levels (**f**) of *HSP22* and *HSP17.6C* in *ER8::JMJ30* transgenic plants in the *jmjq* mutant background subjected to β-estradiol application before acclimation (①) or before heat shock (②). A two-tailed Student’s *t* test between *pER8::JMJ30* transgenic plants in the *jmjq* mutant background subjected to β-estradiol application before acclimation and before heat shock; *p < 0.05.

To investigate whether the timing of demethylation activity is important for heat acclimation, we introduced a *JMJ30* transgene with an estradiol-inducible promoter into the *jmjq* mutant background^26^ (Fig. 4a). Upon application of estradiol, *JMJ30* expression was immediately induced to high levels. A 100-fold increase in *JMJ30* transcript abundance was detected at 0 h after ACC, suggesting that JMJ30 is present immediately after induction (Supplementary Fig. 25). Induction of *JMJ30* before ACC (①) rescued the *jmjq* mutant phenotype, whereas induction just before HS (②) did not (Fig. 4b, c and Supplementary Fig. 26). *jmjq* mutants with JMJ30 induced prior to ACC contained significantly more chlorophyll than *jmjq* mutants with JMJ30 induced prior to HS (Fig. 4c). These results imply that heat memory requires JMJ30 activity prior to acclimation.

We next used RNA-seq to compare the changes in gene expression in *jmjq* mutants with JMJ30 induced prior to ACC versus HS. The identification of differentially expressed genes revealed that similar gene pathways were affected in JMJ30-induced plants prior to ACC and in the *jmjq* mutant (Supplementary Fig. 27 and Supplementary Data 7 and 8). The overlap between these two datasets was significantly larger than expected by chance (*p* = 3.0 × 10^−18^) (Supplementary Fig. 27). As expected, heat acclimation-related genes, including seven out of the eight *HSP* genes downregulated in *jmjq* mutants, were differentially expressed after 4 h of HS when *JMJ30* was significantly induced before ACC, based on RNA-seq (Fig. 4d, Supplementary Fig. 27, and Supplementary Data 7 and 8). Time-course qRT-PCR revealed that induction of *JMJ30* expression prior to ACC enables *jmjq* mutants to sustain *HSP22*, *HSP17.6C*, and *HSP21* expression until at least from 4 h after ACC (Fig. 4e and Supplementary Fig. 28). Furthermore, expression of the three *HSP* genes was activated within 4 h after HS in *jmjq* mutants with JMJ30 induced prior to ACC (Fig. 4e and Supplementary Fig. 28). This activation was significantly delayed in *jmjq* mutants with JMJ30 induced just before HS (Fig. 4e and Supplementary Fig. 28). In addition to an increase in chlorophyll content, higher *HSP* expression was also observed in *jmjq* plants with JMJ30 induced prior to ACC than in the wild type, suggesting that JMJ30 activity in induced plants is higher than that of the endogenous JMJ30 in the wild type (Supplementary Fig. 29). Nevertheless, JMJ30 induction before ACC was responsible for the JMJ-regulated small *HSP* gene expression in response to HS.

To gain more insight into the epigenetic basis of JMJ30 induction, we examined histone modifications by ChIP-qPCR. When the *jmjq* mutant carrying *pER8::JMJ30* was treated with β-estradiol prior to ACC, we detected significant downregulation of H3K27me3 levels at *HSP22-a* and *HSP17.6C-a* initially after 72 h of acclimation (Fig. 4f). No significant differences in H3K27me3 levels at the *TA3* locus were observed in *jmjq* mutants with JMJ30 induced prior to ACC or HS (Supplementary Fig. 30). Taken together, these results demonstrate that JMJ30 induction before ACC mainly functions to maintain proper H3K27me3 levels between ACC and HS for subsequent induction of *HSP* transcription upon heat stress.

### H3K27me3 demethylases control heat acclimation under fluctuating temperature field conditions

**Fig. 5:**
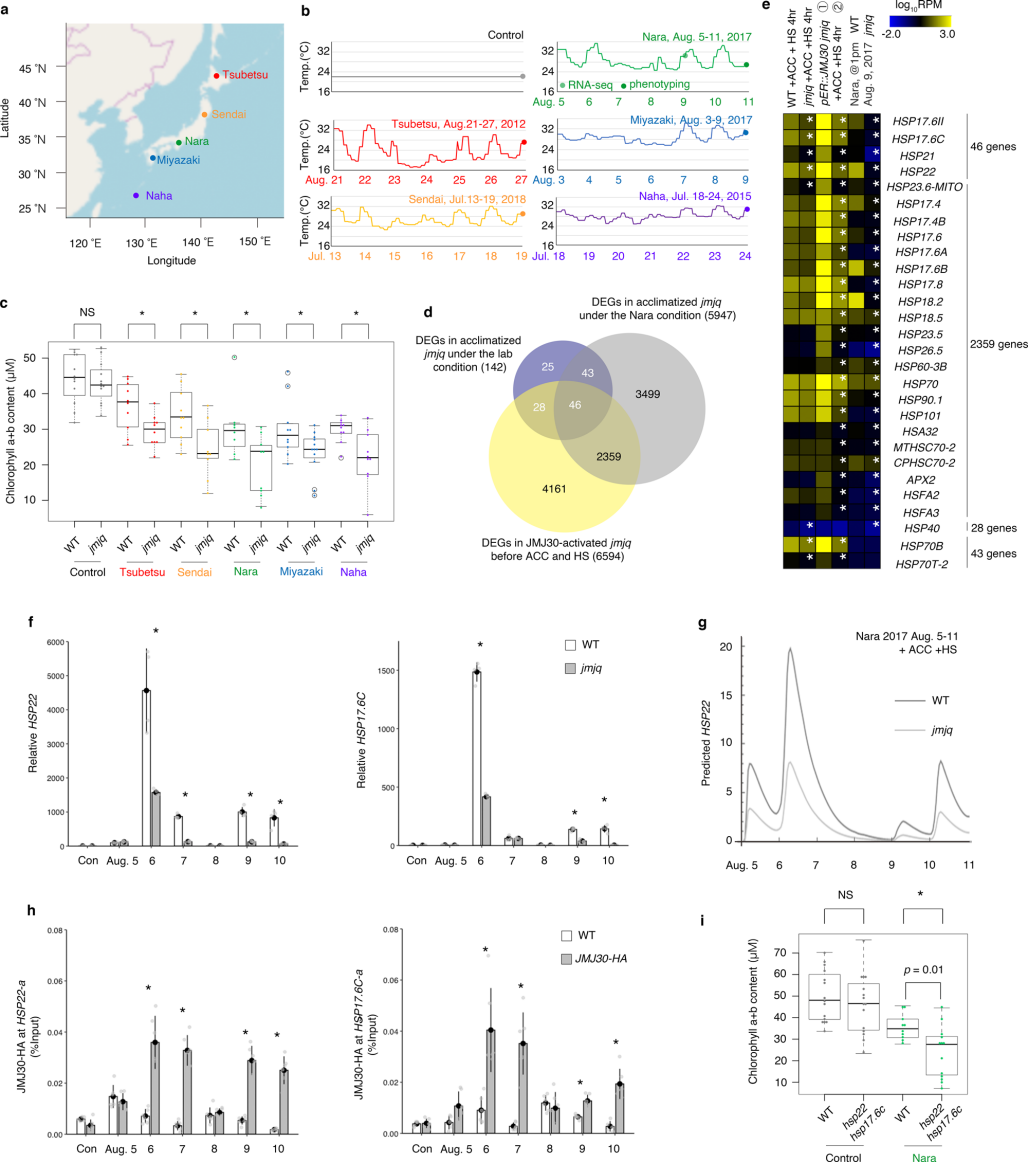
JUMONJI demethylases are required for normal heat response under fluctuating temperature conditions. **a** Locations of cities in Japan whose temperature conditions were mimicked to provide a set of naturally varying growth conditions. Red, orange, green, blue, and purple dots represent Tsubetsu, Sendai, Nara, Miyazaki, and Naha, respectively. **b** Schematic representation of the temperature conditions. Time points for phenotyping are indicated by colored dots. **c** Quantification of chlorophyll contents of plants grown under the five conditions shown in Fig. 5b. Sample minimum (lower bar); lower quartile (box); median (middle line); upper quartile (box); and sample maximum (upper bar). Colored dots and white circles represent the chlorophyll contents of individual samples and statistical outliers, respectively. A two-tailed Student’s *t* test; *p < 0.05). **d** Venn diagram showing the overlaps between genes downregulated in *jmjq* under the lab and Nara conditions. **e** Expression heatmap of genes downregulated in *jmjq* mutants under the Nara condition. White asterisks indicate significant differences between WT and *jmjq* grown under the same conditions or between *pER8::JMJ30* transgenic plants in the *jmjq* mutant background subjected to β-estradiol application before acclimation and before heat shock. The FDR was <0.05. **f** qRT-PCR verification of the *HSP22* (left) and *HSP17.6C* (right) transcript levels in the wild type and *jmjq* grown under the Nara conditions. Gray dots represent the expression levels of individual samples. Asterisks indicate significant differences (*p* < 0.05) between the wild type and *jmjq* at the same time point based on a two-tailed Student’s *t* test. **g** Mathematical model prediction for *HSP22* expression under the Nara condition. **h** JMJ30-HA levels in the wild type and *pJMJ30::JMJ30-HA* (**h**), as determined by ChIP-qPCR. Gray dots represent the expression levels of individual samples. A two-tailed Student’s *t* test between the wild type and *jmjq* or the *pJMJ30::JMJ30-HA* line at the same time point; *p < 0.05. **i** Quantification of chlorophyll contents in wild-type and *hsp22 hsp17.6c* plants grown under the Nara condition. One-way ANOVA test; *p < 0.05. Different letters indicate significant differences based on a post-hoc Tukey’s HSD test (*p* < 0.05). NS, nonsignificant. *n* > 9.

To address the key function of JMJs in heat acclimation under natural conditions, we grew the wild type and the *jmjq* mutant under fluctuating temperature conditions based on actual meteorological data from five Japanese cities at different latitudes^27^ (Fig. 5a, b and Supplementary Data 9). Four-day-old seedlings grown in the laboratory at 22 °C were transferred to fluctuating temperature conditions, which included at least two temperature spikes to >30 °C separated by a 2-day gap. Under these fluctuating conditions, we referred to the first spike >30 °C as ACC and to the second spike as HS (Fig. 5b). Under all five of the fluctuating temperature conditions, the *jmjq* mutant contained significantly less chlorophyll than the wild type (Fig. 5c).

To examine the molecular basis of JMJ function in the response to naturally fluctuating temperatures, RNA-seq was performed 4 h after the second heat spike >30 °C in the treatment that mimicked temperatures in the city of Nara (Fig. 5b circle). The transcript levels of JMJ-regulated *HSP* genes, including *HSP17.6C*, *HSP17.6II*, *HSP21*, *HSP22*, and *HSP23.6-MITO*, were all reduced in *jmjq* mutants grown under the Nara temperature conditions, similar to mutants grown under the standard laboratory conditions. By contrast, all five genes were significantly induced when *JMJ30* expression was induced in the mutant before ACC (Fig. 5d, e, Supplementary Fig. 31, and Supplementary Data 10–12).

To further understand epigenetic control of gene expression under natural temperature conditions, we examined the effect of JMJs on gene expression and histone modification. qRT-PCR detected high temperature-dependent *HSP22* and *HSP17.6C* transcript fluctuations in the wild type grown under the Nara conditions (6, 7, 9, and 10 Aug 2017 at 13:00) (Fig. 5f). This upregulation was significantly attenuated in the *jmjq* mutant (Fig. 5f). The stochastic model predicted that *HSP22* gene expression would be reduced after heat stress in acclimated *jmjq* mutants under the Nara temperature conditions (Fig. 5g). Furthermore, JMJ30 directly bound to *HSP22* and *HSP17.6C* genes in response to heat (6, 7, 9, and 10 Aug 2017 at 13:00) (Fig. 5h), whereas JMJ30 binding was not observed at the *TA3* locus (Supplementary Fig. 32). This fluctuation of JMJ30-HA binding could be partially due to stabilization of JMJ30 mRNA^4^ (Supplementary Fig. 33).

To address the contribution of *HSP22* and *HSP17.6C* to JMJ downstream effects under fluctuating field conditions, we examined the phenotype of *hsp22 hsp17.6c* double mutant plants. The double mutants exhibited reduced chlorophyll contents relative to the wild type under the Nara conditions, but not under the control conditions (Fig. 5i). However, this reduction was less significant than the reduction observed for *hsp22 hsp17.6c* double mutants grown under laboratory conditions (Figs. 3k and 5i). This difference in the severity of the phenotype could be due to differences in the activity of other *HSP* or heat stress-induced genes. Indeed, many other *HSP* or heat stress-induced genes were differentially expressed in *jmjq* mutants. In addition to the five small *HSPs* studied here, many other small *HSPs* were also differentially expressed in acclimated *jmjq* mutants under the Nara conditions, based on RNA-seq (Fig. 5e). qRT-PCR also revealed that a handful of heat response genes showed expression patterns similar to those of *HSP22* and *HSP17.6C* in wild-type plants grown under the Nara conditions (Supplementary Fig. 34). Furthermore, the expression levels of *HSP18.2* and *HSFA2* genes were significantly decreased in *jmjq* mutants grown under the Nara conditions (Supplementary Fig. 34). Among those genes, JMJ30 directly bound to the gene body of the *HSP18.2* gene after heat stress (Supplementary Figs. 32). These results suggest that other condition-dependent gene(s) in addition to *HSP22* and *HSP17.6C* may promote heat acclimation under fluctuating field temperature conditions.

**Fig. 6:**
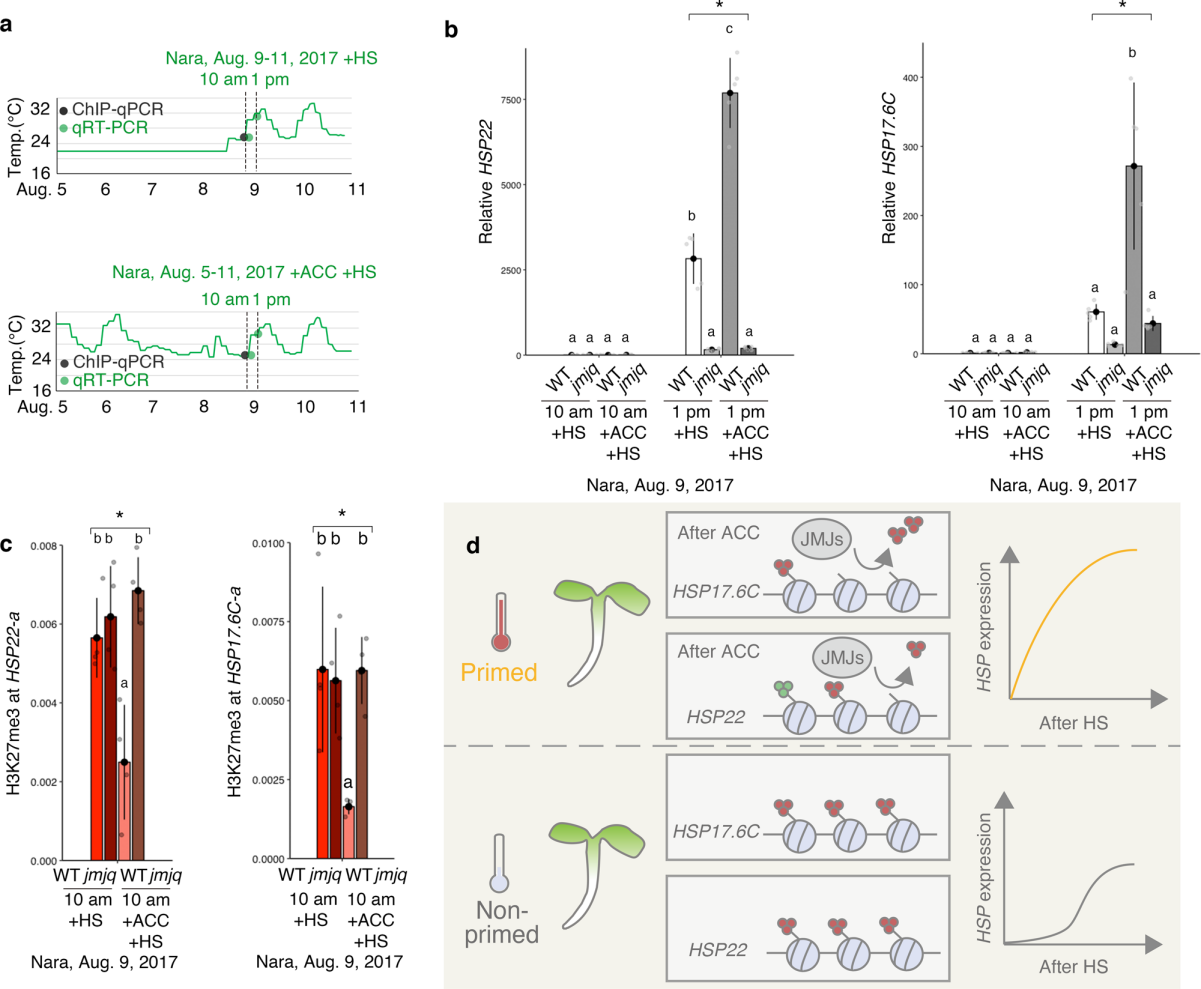
JUMONJI demethylases are required for the removal of H3K27me3 histone modifications from heat-memory genes under fluctuating temperature conditions. **a** Schematic representation of the temperature conditions used. Colored dots indicate the times of different assays: qRT-PCR, red; ChIP-qPCR, gray. Upper, basal thermotolerance condition (+HS); Lower, heat-stress memory condition (+ACC +HS). **b** Gene expression levels of *HSP22* and *HSP17.6C* in the wild type and *jmjq* grown under the Nara conditions. Gray dots represent the expression levels of individual samples. One-way ANOVA test; *p < 0.05. Letters above bars indicate significant differences based on a post-hoc Tukey HSD test (*p* < 0.05). **c** H3K27me3 levels at *HSP22* and *HSP17.6C* in the wild type and *jmjq* grown under the Nara conditions. One-way ANOVA test; *p < 0.05. Different letters above bars indicate significant differences based on a post-hoc Tukey HSD test (*p* < 0.05). **d** Current model of JUMONJI-mediated heat acclimation. Upon exposure to a triggering heat, plants are acclimatized. Three red and green circles represent H3K27me3 and H3K4me3, respectively. JMJ proteins keep H3K27me3 at lower levels on *HSP17.6C* and *HSP22*. The primed plants respond to recurring heat faster or more strongly than non-primed plants do.

To examine further the role of JMJs in heat memory, wild-type and *jmjq* mutant plants were grown with or without ACC under fluctuating temperature conditions prior to gene expression and JMJ30 binding assays (Fig. 6a). qRT-PCR confirmed that acclimatized *jmjq* mutants had lower levels of *HSP22*, *HSP17.6C*, and *HSP21* expression 4 h after exposure to HS under the Nara conditions compared to the wild type (9 Aug 2017 at 13:00). By contrast, there was no difference between the wild type and *jmjq* mutants in the expression of the same genes immediately after HS under the same conditons (9 Aug 2017 at 10:00) (Fig. 6a, b and Supplementary Fig. 35). Furthermore, upregulation of *HSP22*, *HSP17.6*, and *HSP21* was significantly compromised in the absence of ACC between 5 Aug and 9 Aug (Fig. 6a, b). These results suggest that JMJ-mediated transcriptional changes are required at least in part to remember recurring heat, even under natural conditions.

To further test the hypothesis that histone modifications underlie the heat memory-related transcriptional changes, H3K27me3 levels at *HSP22* and *HSP17.6C* were examined by ChIP-qPCR. Without ACC between 5 Aug and 9 Aug, we observed no differences in the H3K27me3 levels at *HSP22* and *HSP17.6C* between 8-day-old wild-type and *jmjq* mutant plants grown under control conditions. Before HS under the Nara temperature conditions, the *jmjq* mutant had higher levels of H3K27me3 at *HSP17.6C* and *HSP22* than the wild type (Fig. 6a, c). Because these two datasets were obtained from tissues at the same developmental stage, the changes in histone modifications were largely caused by the stress memory rather than developmental effects. H3K4me3 levels at *HSP17.6C* were not affected by ACC (Supplementary Fig. 36). Unlike under laboratory conditions, the wild type had higher levels of H3K4me3 at *HSP22* than the *jmjq* mutant before HS under the Nara conditions (Supplementary Fig. 36). Under this condition, the accumulation of H3K4me3 at *HSP22* in the wild type could be long lasting and this could be partially due to higher *HSFA2* expression, as reported previously^14^. Furthermore, H3 levels were unaffected (Supplementary Fig. 37). Thus, the epigenetic memory implemented by H3K27me3 demethylases might facilitate environmental adaptation under natural conditions, although additional pathways probably also exist.

## Discussion

In this study, we have uncovered a molecular mechanism for heat memory in *A. thaliana* based on the modulation of H3K27me3 histone modification at small *HSP* genes by JMJs. Our results suggest that H3K27me3 demethylases specifically control the level of histone modification at *HSP22* and *HSP17.6C* genes by altering their reinduction to maintain heat memory. In response to ACC, JMJs contribute to induce faster *HSP22* and *HSP17.6C* expression after HS. Five pieces of evidence support this. First, the *jmjq* mutant showed a reduced ability to remember heat. Second, the expression of *HSP22* and *HSP17.6C* genes was significantly delayed in *jmjq* mutants than in the wild type in response to heat. Third, the level of H3K27me3 was significantly higher in the *jmjq* mutant than in the wild type, due to the failure to remove H3K27me3 in the mutant. Fourth, JMJ30 directly bound to the *HSP22* and *HSP17.6C* loci in response to heat. Fifth, induction of JMJ30 in the *jmjq* mutant partially rescued not only its acclimation defects, but also its changes in gene expression and histone modification. Therefore, the maintenance of H3K27me3 at *HSP22* and *HSP17.6C* by JMJs is likely to render these genes more sensitive to induction by subsequent heat (Fig. 6d). The proper removal of H3K27me3 at *HSP22* and *HSP17.6C* through JMJs is required for their repression during the memory phase (i.e., between ACC and HS). While ectopic expression of JMJ30 were able to remove H3K27me3, triple mutant did not have excess H3K27me3 and corresponding phenotypes (Figs. 1, 4 and Supplementary Fig. 18). Since, hyper trimethylation of H3K27me3 was observed in *jmjq* mutant (Fig. 3 and Supplementary Fig. 18), this could be due to functional redundancy. To understand specific contribution of any of individual JMJ proteins, genome-wide identification of JMJ30, JMJ32, REF6, and ELF6 targets during heat acclimation is required. Some genes are known to exhibit elevated expression levels between ACC and HS for heat acclimation^2^. But, our selection criteria is not designed for screening out such genes. Although a large set of stress-inducible genes are hypertrimethylated in *jmjq* compared to the wild type without heat stress (Fig. 3b), the role of those genes has not been characterized yet. To fully understand the role of JMJs on heat acclimation, further analysis is needed.

A previous report^16^ suggested that expression of the *REF6* is regulated by heat under prolonged high temperatures, but we detected no REF6 binding at *HSP* genes during heat acclimation under our heat acclimation conditions. Different H3K27me3 demethylases may accumulate depending on the temperature and duration of the heat stress. Our findings that *HSP* genes were still induced in *jmjq* mutants in response to recurring heat and that acclimation improved the ability of the mutant to survive heat stress suggested that additional, redundant H3K27me3 demethylase(s), such as JMJ13, and/or parallel pathway(s) contribute to heat acclimation. Furthermore, H3K27me3 levels are determined by the balance between the activities of histone demethylases and methyltransferases acting on H3K27. Although the Polycomb Group-mediated H3K27me3 methylation plays a critical role in plant growth and development^28,29^, nothing is known about the function of these methyltransferases during heat acclimation. In *jmjq* mutants, H3K27me3 levels at *HSP22* and *HSP17.6C* were significantly increased within 24 h after ACC (Fig. 3d). The active removal of H3K27me3 from the gene body by JMJs is important for the activation of Polycomb-silenced genes during the developmental transition or in response to the internal or external stimuli^8^. Also, the plant H3K27me3 demethylases function not only delimiting Polycomb-silenced chromatin regions but also preventing the uncontrolled spreading of the epigenetic silencing^8^. During the memory phase, such mechanisms could be important for the proper H3K27me3 maintenance. A better understanding of the interactions between methyltransferases and demethylases is an important goal for future studies to uncover the molecular mechanisms underlying heat memory-mediated modulation of H3K27me3.

As expected, based on numerous previous studies^15,22^, there was a negative correlation between H3K27me3 and H3K4me3 levels at *HSP22* throughout the memory phase, which is also consistent with a requirement of the mathematical model established for *HSP22*. In *jmjq* mutants, H3K4me3 at *HSP22* were not accumulated. Our result suggests that the H3K27me3 removal and H3K4me3 deposition may be interconnected. A previous report showed that HSFA2 was essential for H3K4me3 accumulation at the *HSP22* locus after ACC^14^. During the plant development, transcription factors act as a docking point for the different histone modification enzymes^30^. Such a mechanism might lead to the incorporation of the H3K27me3 removal and the H3K4me3 deposition to render the *HSP22* gene more sensitive to induction by subsequent heat.

In the case of *HSP17.6C*, however, H3K4me3 levels increased in concert with gene expression only after heat exposure. This result suggests that deposition of permissive histone modification(s) other than H3K4me3 at *HSP17.6C* counteracts the inhibitory effects of H3K27me3. Other research has found no H3K4me3 accumulation at *HSP70* after heat stress regardless of the transcriptional activity of this gene^14,31^. Therefore, it is likely that H3K4me3 enrichment does not always accompany transcriptional activation of *HSP* genes. The timing of deposition varies with different permissive histone modifications^32^, and their combinatorial effects on epigenetic modifications and the associated factors that bind neighboring chromatin sequences could be different for different genes. Thus, time-courses and comprehensive genome-wide analyses of histone modifications will be needed to better understand the interactions between histone modifications and their effects on gene expression.

In a parallel approach, genetic experiments using higher-order *hsp* mutants indicate that *HSP22* and *HSP17.6C* play important roles during heat acclimation in *A. thaliana*. Based on differences in the severity of the phenotypes of the *jmjq* mutant and the *hsp22 hsp17.6c* double mutant, we conclude that *HSP22* and *HSP17.6C*, along with additional genes or mechanisms, contribute to increased survival after heat acclimation. The additional genes might include the other differentially expressed *HSP* genes we identified in this study. Additional analyses of different histone modifications (other than H3K27me3) and changes in chromatin structure at multiple time points will reveal whether these candidate genes play regulatory epigenetic roles in enhancing plant survival. Furthermore, plants have evolved many acclimation strategies, including memory-based strategies involving proteins and metabolites^33,34^. To completely understand the heat acclimation phenotype of the *jmjq* mutant will require additional, less chromatin-biased analyses at multiple levels.

Interestingly, orthologs of key heat-memory genes, such as *Ahg474482* (*HSP17.6C*), *Ahg489869* (*HSP22*), and *Ahg945273* (*HSP21*), were highly expressed in field-grown *Arabidopsis halleri* during the summer^35^. These results suggest that these demethylases play a key role in heat acclimation and *HSP* regulation in the field. Notably, more genes, such as *HSP18.2* and *HSFA2*, were differentially expressed in *jmjq* mutants under fluctuating field temperature conditions than under laboratory conditions. Furthermore, the differences in gene expression and histone modification between the wild type and the *jmjq* mutant were larger under fluctuating field temperature conditions than they were under laboratory conditions. One explanation of this difference could be the previously reported stabilization of *JMJ30* mRNA and JMJ30 proteins by heat^4^. However, differentially expressed genes between *jmjq* mutants under fluctuating field temperature and JMJ30-induced lines also are different, suggesting that a threshold mechanism of all-or-none transcriptional target activation by JMJ30 proteins is not the only explanation. In other words, additional regulatory mechanisms or pathways other than JMJ30 stabilization probably exist. Fluctuating field temperature-regulated associated co-activator(s) might allow JMJ complexes to bind different DNA binding sites and regulate additional target genes.

Further support for our findings comes from a recent study of the acquisition of freezing tolerance in response to rapid versus gradual decreases in temperature^36^. Plants might have evolved more than one system to respond to and remember different rates of temperature change. Together with H3K27me3, other histone modifications or epigenetic changes, such as H3K36me3, DNA methylation, and chromatin structure alterations, might also contribute to heat-stress memory^21,23,37^. Stress-induced nucleosome remodeling mediated by the FORGETTER (FGT) complex might be involved in this regulation, since direct targets of FGT, such as *HSP18.2* and *HSA32*, were identified as differentially expressed genes in *jmjq* mutants only under fluctuating field temperature (Fig. 5e). In addition to co-activators, epigenetic modifications and associated factors that bind neighboring chromatin sequences might contribute to additional target regulation. Whether histone modification and nucleosome abundance at memory genes act together or independently remains to be established. Alternatively, H3K27me3 demethylases might remove repressive histone marks repeatedly in response to each and every recurring heat stress under fluctuating field temperature conditions. Moreover, these two possibilities are not mutually exclusive. Our findings, coupled with potential future discoveries, will further elucidate the mechanisms of plant adaptation and memory.

## Methods

### Plant materials and growth conditions

*Arabidopsis thaliana* plants were grown at 22 °C under continuous light unless otherwise specified. Culture medium consisted of half-strength Murashige and Skoog (MS) salts (Nacalai Tesque) and 0.8% agar (Nacalai Tesque) at pH 5.6 was used for all experiments except for crossing and transformation. For mature plant materials, seeds were sown in pots containing vermiculite and Metro-Mix. The following plant lines were previously described: *jmj30-2*^4^, *jmj32-1*^4^, and *jmj30-2 jmj32-1*^4^ mutants and *pJMJ30::JMJ30-HA jmj30-2*^4^; *elf6-3*^38^, *ref6-1*^38^, and *jmj13G*^8^ (Supplementary Data 13); and *hsp22*^18^; *hsfa1a/b/d*^11^, and *hsfa2*^9^. The *hsp17.6c-1* (SALK_56782) mutant line was obtained from the Arabidopsis Biological Resource Center. All plants were in the Columbia background. Genotyping primers are listed in Supplementary Data 14.

### Plasmid construction and plant transformation

For the *GUS* constructs, the genomic regions of the *HSP22*, *HSP17.6C*, and *HSP21* loci, including sequence upstream of the translational start site, and coding regions, and excluding the translation termination codon, were amplified by PCR using PrimeSTAR GXL DNA polymerase (Takara) and gene-specific primer sets. The resulting PCR products were subcloned into the pENTR/d-TOPO vector (Thermo Fisher Scientific). After sequences were confirmed with an ABI3130 sequencer (ABI), the insert fragments were transferred into the pBGWF7 destination vector^39^ using LR clonase (Thermo Fisher Scientific). All the *GUS* constructs were transformed into wild-type plants by floral dip using *Agrobacterium tumefaciens* (GV3101)^40^. T_1_ plants grown on soil were treated with the nonselective herbicide Basta. T_2_ seeds were harvested from Basta-resistant plants. *GUS* expression testing with and without heat treatment was performed using at least 15 independent T_2_ plants. Representative lines in the wild type were established first. Subsequently, gHSP22-GUS, gHSP17.6C-GUS, and gHSP21-GUS were crossed into *jmjq* mutants. Thus, GUS staining and the MUG assay in the wild type and *jmjq* mutants were conducted using transgenes located in the same genomic position. Cloning primers are listed in Supplementary Data 14.

For the overexpression construct, the *HSP17.6C* cDNA was amplified by PCR using PrimeSTAR GXL DNA polymerase (Takara) and gene-specific primer sets. cDNA prepared from *A. thaliana* Columbia was used as a template. The resulting PCR products were subcloned into pENTR/d-TOPO vector (Thermo Fisher Scientific). After sequences were confirmed with an ABI3130 sequencer (ABI), the insert fragments were transferred into the pB2GW7.0 destination vector^38^ using LR clonase (Thermo Fisher Scientific). The construct was transformed into *jmjq* plants by floral dip using *Agrobacterium* (GV3101)^40^. T_1_ plants grown on soil were treated with Basta. T_2_ seeds were harvested from Basta-resistant plants. Expression levels of *HSP17.6C* were examined using more than 15 independent T_2_ plants. Representative lines were used for further analysis. Cloning primers are listed in Supplementary Data 14.

For the *pER8::JMJ30* construct, *JMJ30* cDNA was amplified by PCR using gene-specific primer sets with restriction enzyme sequences. cDNA prepared from *Arabidopsis* Columbia was used as a template. The amplified DNA fragment was Gateway-cloned into pENTR/d-TOPO vector (Thermo Fisher Scientific). After sequencing using vector-specific primers, the fragment was introduced into the pER8 vector^26^. The construct was transformed into wild-type plants by floral dip using *Agrobacterium* (GV3101)^40^. T_1_ plants were grown on MS medium supplemented with hygromycin for transgenic selection. More than 20 independent inducible lines were further screened with phenotypic and expression analyses. Representative lines were used for further analysis. Cloning primers are listed in Supplementary Data 14.

### Heat stress treatment

*Arabidopsis* seeds were sterilized with 1 ml of 100% bleach in a 1.5-ml tube for <1 min. After being washed at least three times with sterilized water, seeds were sown on half-strength MS medium containing 0.8% agar. The plates were kept at 4 °C for 5–7 days to synchronize seed germination. Plates were then moved into growth chamber at 22 °C under continuous light. After 24 h, radicle tip emergence (germination) was observed regardless of genotype. When required, seedlings were subjected to heat-stress treatment. For heat-stress treatment in laboratory conditions, a PERSONAL-11 water bath shaker (TAITEC) was used^41^. Plates were placed into re-sealable zipper storage bags (S.C. Johnson & Son) and submerged in the water bath. Two kinds of stress were used: acclimation treatment (37 °C for 20 min) and heat-shock treatment (43.5 °C for 60 min) were performed on 4-day-old (96 h after germination) and 7-day-old (168 h after germination) seedlings, respectively. After each heat treatment, seedlings were returned to the growth chamber and allow to recover for further experiments.

To grow plants under fluctuating temperature conditions, seeds were prepared as described above. Plants were grown for 4 days after germination in a growth chamber at 22 °C under continuous light. Then, the plants were moved and grown in a SGCmini growth chamber (Clockmics inc.) under fluctuating temperature conditions. The conditions used followed the actual environmental data for Tsubetsu (lat. 43°42.1′ N. and long. 144°2.0′ E.) from August 21 to 27, 2012; Sendai (lat. 38°15.7′ N. and long. 140°53.8′ E.) from July 13 to 19, 2018; Nara (lat. 34°41.6′ N. and long. 135°49.6′ E.) from August 4 to 10, 2018; Miyazaki (lat. 31°56.3′ N. and long. 131°24.8′ E.) from August 3 to 9, 2017; and Naha (lat. 26°12.4′ N. and long. 127°41.2′ E.) from July 18 to 24, 2015. Past temperature data from five cities at different latitudes in Japan were obtained from the Japan Meteorological Agency (https://www.data.jma.go.jp/obd/stats/etrn/index.php). The temperature in the SGCmini growth chamber was changed for 6 days following the pattern for a given city. Japan map was generated by R (Version 3.5.2) using the data from Natural Earth (https://www.naturalearthdata.com/). Temperatures recorded in the SGCmini growth chamber every hour are listed in Supplementary Data 9.

### Estradiol treatment

For β-estradiol treatment, the compound was dissolved in dimethyl sulfoxide (DMSO) just prior to use. For mock treatment, the same amount of DMSO was used as control. Plants were grown on MS medium without β -estradiol and then transplanted onto MS medium with 10 µM β -estradiol^26^ using ethanol-sterilized forceps in a hood. Once we started treatment, plants were transplanted every 2 days.

### Phenotypic analyses

For all phenotypic analyses, plants to be directly compared were grown side by side at the same density per plate to minimize potential microenvironmental differences in the growth chamber. To determine the survival rate, phenotypic strength in the wild type and each mutant were categorized into three different classes^42^. If plants were vigorous and looked entirely green, those plants were categorized as “normal”. If plants were largely pale, those were counted as “partially damaged”. Dead plants were categorized as “perished”. *n* > 138. At least three independent experiments were performed, and similar results were obtained. Representative plate images were photographed with a Nikon D750 camera. A chi-squared (*χ*^2^) test, followed by post-hoc test, was performed through IBM SPSS Statistics 26 (IBM).

For chlorophyll measurement, total chlorophyll levels were measured using *N*,*N*′-dimethylformamide (DMF) extraction and spectrophotometric quantification^43^. Five seedlings were placed into a 2-ml tube containing 1 ml of DMF. The experiments were repeated more than five times. *n* > 5. Tubes were incubated overnight at 4 °C. The absorbance at 646.8 and 663.8 nm was measured in 1.00-cm cuvettes on spectrophotometer (IMPLEN NanoPhotometer P-Class). Total chlorophyll was calculated as reported previously^43^ (Chl *a* + *b* (µM) = 19.43 *A*_646.8_ + 8.05 *A*_663.8_). Maximum, 75th percentile, 50th percentile, 25th percentile, and minimum values are shown in the graphs. At least three independent experiments were performed, and similar results were obtained. Normal distribution was verified by the Kolmogorov–Smirnov test for all analyses. Statistical significance was computed using a one-way ANOVA test followed by post-hoc Tukey’s HSD test (https://astatsa.com/OneWay_Anova_with_TukeyHSD/).

For fresh weight assay, 10-day-old seedlings were used. Ten *Arabidopsis* seedlings were harvested in a 2-ml tube and measured with an analytical balance^44^ (Mettler Toledo XS104). *n* > 11. Maximum, 75th percentile, 50th percentile, 25th percentile, and minimum values are shown in the graphs. At least three independent experiments were performed, and similar results were obtained. Normal distribution was verified by the Kolmogorov–Smirnov test for all analyses. Statistical significance was computed using one-way ANOVA test followed by post-hoc Tukey’s HSD test (https://astatsa.com/OneWay_Anova_with_TukeyHSD/).

For leaf measurement, 5th true leaves from 10-day-old seedlings were used. Leaves were dissected using forceps and surgical scissors. Images were taken with a Nikon D750 camera and were used to measure leaf area with ImageJ software (http://rsb.info.nih.go.ij/). To quantify cell size and number, leaves were nicked at the edges and the resulting samples were placed into fixation solution and placed under vacuum for 20 min for infiltration. The resulting leaf tissue samples were transferred into clearing solution and kept for at least 16 h at room temperature. The samples were then mounted onto microscope slides with one or two drops of clearing solution, and imaged using an AxioScope A1 microscope (Zeiss). The cell area was measured with ImageJ software (http://rsb.info.nih.go.ij/). Leaf and cell areas were subsequently used to calculate cell numbers^45^. Values in graphs are mean ± SEM. Statistical significance was computed using one-way ANOVA test followed by post-hoc Tukey’s HSD test (https://astatsa.com/OneWay_Anova_with_TukeyHSD/).

### Scanning electron microscopy

For scanning electron microscopy (SEM), the aboveground parts of 10-day-old seedlings were placed in FAA (45% EtOH, 2.5% formaldehyde, and 2.5% acetic acid), placed under vacuum until the tissues sank into the fixative, and left at least 16 h at room temperature. Tissues were then transferred successively through a gradient series of ethanol solutions (50%, 60%, 70%, 80%, 90%, 95%, 100% × 2) in water for 20 min each, and then a gradient series of acetone (25%, 50%, 75%, 95%, 100% × 2) in ethanol for 30 min each. Then, the tissues were critical-point-dried with liquid CO_2_ in an EM CPD300 critical-point dryer (Leica Microsystems) and gold-coated with E-1010 (Hitachi) prior to SEM imaging. The tissues were imaged under an S-4700 SEM (Hitachi) with an accelerating voltage of 15 kV. At least five shoot apical meristems for each genotype and treatment were observed, and representative images are shown.

### RNA analyses

For RNA extraction, total RNA was extracted from whole seedlings using the RNeasy Plant Mini Kit (Qiagen). About 100 mg of plant tissues was harvested, immediately frozen in liquid nitrogen, and kept at −80 °C until use. The tissues were ground to a fine powder with ice-cold mortar and pestle. RNA extraction was performed following the manufacturer’s instructions. An RNase-Free DNase Set (Qiagen) was used to remove genomic DNA. Concentration was measured by spectrophotometer (IMPLEN NanoPhotometer P-Class).

For qRT-PCR, cDNA was synthesized from 100 ng of RNA using a PrimeScript 1st strand cDNA Synthesis Kit (Takara). The resulting cDNA was quantified with a LightCycler 480 (Roche) using FastStart Essential DNA Green Master mix (Roche). The signals were normalized against the internal control gene *UBIQUITIN CONJUGATING ENZYME 21*^46^ (*UBC21*; *AT5G25760*). Values in graphs are mean ± SEM. At least, three independent experiments were performed. Each result is shown by jitter plot. Statistical significance was computed using a two-tailed Student’s *t* test. Primers for qRT-PCR are listed in Supplementary Data 14.

For RNA-seq, libraries were prepared as reported previously^47^ and sequenced by HighSeq 2500 using 50-base-pair single-end mode (Illumina). Mapping was conducted using the *Arabidopsis thaliana* reference genome (TAIR10). The read count for each gene was calculated by RSEM^48^. After normalization, FDR and FC were calculated using the edgeR package^49^ following likelihood raito test (version 3.24.3) for R (Version 3.5.2). Genes with false discovery rate (FDR) <0.05 in each comparison were identified as differentially expressed genes. The agriGO web-based tool (version 1.2) and database (http://bioinfo.cau.edu.cn/agriGO/) was used for a Gene Ontology (GO) term enrichment analysis^50,51^. *p*-values were calculated based on two-tailed *z*-test. Heatmaps and *k*-means clustering graphs were generated with MeV (version 4.8.1) (http://mev.tm4.org/#/welcome). Optimal six clusters were identified using Pearson correlation. The sequence data were deposited into the DNA Data Bank of Japan (DRA008818, DRA009425).

### Trypan blue staining

To identify dead cells, trypan blue staining was performed as previously described with minor modification^52^. Cotyledons were collected into 1.5-ml tubes containing 0.05% trypan blue (Nacalai Tesque). The tubes were then boiled for 1 min to stain dead cells. After staining, tissues were transferred into chloral hydrate solution and kept at least 6 h. The resulting tissues were placed on glass slides, mounted in a drop of 50% glycerol, and immediately observed under an AxioScope A1 microscope (Zeiss) equipped with an AxioCam ERc 5s camera (Zeiss) and analyzed using the ZEN2 software (Zeiss). At least five cotyledons were observed, and representative images are shown.

### DAB staining

To observe reactive oxygen species (ROS) accumulation, H_2_O_2_ staining was conducted as previously described with minor modification^53^. Cotyledons were collected into 1.5-ml tubes and stained using the peroxidase stain DAB Kit (Nacalai Tesque) for 2 h in darkness with gentle shaking. After staining, tissues were transferred to fresh tubes containing bleach solution (60% ethanol, 20% acetic acid, and 20% glycerol) and boiled for 15 min to decolorize chlorophyll. The resulting tissues were placed on glass slides, mounted on a drop of 50% glycerol, and immediately observed under an AxioScope A1 microscope (Zeiss) equipped with an AxioCam ERc 5s camera (Zeiss) and analyzed using the ZEN2 software (Zeiss). More than five cotyledons were observed, and representative images are shown.

### Immunoblotting

For GUS staining, protein extracts were prepared from 10 mg of seedlings in 10 µl of SDS sample buffer. All of the eluate was loaded onto a 4–12% SDS-PAGE gel (Invitrogen) and the gel was run in a Bolt Mini Gel tank (Thermo Fisher Scientific). The resulting gel was subjected to immunoblot analysis using the iBlot2 dry blotting system (Thermo Fisher Scientific). Rabbit polyclonal anti-HSP22 (Eurofins Genomics) (1:1000 diluted), anti-HSP17.6 (ab80183; Abcam) (1:1000 diluted), and anti-HSP21 (ab80175; Abcam) (1:1000 diluted) and anti-rabbit IgG HRP conjugate (1:5000 diluted, Thermo Fisher Scientific) were used as primary and secondary antibodies, respectively. Signals were detected using chemiluminescence HRP substrates (Millipore) and an image analyser (LAS4000, GE healthcare). Protein size was determined by MagicMark XP (Thermo Fisher Scientific). Coomassie Brilliant Blue (CBB)-stained membranes were used as loading controls. The signal intensity of each band was quantified by ImageJ (NIH). Values in graphs are mean ± SEM. Three independent experiments were performed. Statistical significance was computed using a one-way ANOVA test followed by a post-hoc Tukey’s HSD test (https://astatsa.com/OneWay_Anova_with_TukeyHSD/).

### GUS expression analysis

For GUS staining, tissues were kept in 90% acetone for 20 min to infiltrate solution. Tissues were rinsed with GUS staining buffer without 5-bromo-4-chloro-3-indolyl-β-d-glucuronic acid (X-Gluc) three times and put into GUS staining buffer with X-Gluc. They were placed under vacuum until the tissues sank, and then left overnight or for two nights, depending on the expression levels. After GUS staining, chlorophyll was removed by allowing tissues to sit in 70% EtOH for at least 1 week. Representative images were taken under an AXIO Zoom.V16 (Zeiss) microscope.

To quantify GUS activity, a MUG assay was performed as previously described with minor modification^54^. Ten-day-old seedlings were harvested, immediately frozen in liquid nitrogen, and kept at −80 °C until use. Tissues were homogenized in extraction buffer and protein solution was obtained after removal of debris by centrifugation. Reaction buffer containing 4-methylumbelliferyl-β-d-glucuronide (4-MUG) was mixed with the resulting protein solution. Reactions were stopped after 0 and 60 min incubation at 37 °C by adding stop buffer. 4-MU fluorescence was measured with a Tristar^2^-LB942 microplate reader (BERTHOLD) using an excitation and emission wavelengths of 365 and 455 nm, respectively. Protein amounts were determined using a Qubit4 Fluorometer and Qubit Protein Assay kit (Thermo Fisher Scientific). GUS activity was calculated in (nmol 4-MU) min^−1^ (mg protein)^−1^. Statistical significance was computed using one-way ANOVA test followed by post-hoc Tukey’s HSD test (https://astatsa.com/OneWay_Anova_with_TukeyHSD/).

### Chromatin immunoprecipitation

For ChIP-qPCR, ChIP was carried out as described below^55^. For each sample, 100–300 mg of seedlings were fixed with 1% formaldehyde for 15 min. After quenching of formaldehyde with glycine for 5 min, tissues were frozen in liquid nitrogen and kept at −80 °C until use. Tissues were ground to a fine powder with an ice-cold mortar and pestle. Using nuclei extraction buffer, chromatin was isolated from a nuclear extract. Fragmentation was conducted using an Ultrasonic Disruptors UD-201 sonicator (TOMY). After preclearing, antibodies were added and the mixtures were rotated overnight at 4 °C. H3K4me3 (ab8580; Abcam; 1 µl), H3K27me3 (ab6002; Abcam; 2 µl), H3 antibodies (ab1791; Abcam; 1 µl), and HA (12CA5; Roche; 5 µl) were used. For immunoprecipitation to capture DNA-protein complexes, Dynabeads Protein A or G (Thermo Fisher Scientific) were used. Beads were washed and DNA was eluted from beads overnight at 65 °C. The resulting DNA was purified using QIAquick PCR Purification Kit (Qiagen). DNA was quantified with a LightCycler 480 (Roche) using FastStart Essential DNA Green Master mix (Roche). The ratio of ChIP over input DNA (% Input) was compared based on the reaction threshold cycle for each ChIP sample compared to a dilution series of the corresponding input sample. The *TA3* retrotransposon (*AT1G37110*) was used as the negative control region for ChIP-qPCR. Values in graphs are mean ± SEM. At least. three independent experiments were performed. Each result was shown by jitter plot. Statistical significance was determined by one-way ANOVA followed by post-hoc Tukey’s HSD test for multiple-pair comparisons or a two-tailed Student’s *t* test for single-pair comparisons. Primers for qPCR are listed in Supplementary Data 14.

ChIP-seq was performed as previously described with minor modifications^56^. In all, 1.5 g of seedling tissues were frozen in liquid nitrogen and kept at −80 °C before use. Tissues were ground to a fine powder with an ice-cold mortar and pestle and post-fixed in nuclei isolation buffer for 10 min. Glycine was added and kept for 5 min at room temperature. After removal of debris with a Miracloth (Merck), chromatin was dissolved into ChIP dilution buffer and sonication was conducted by S2 sonicator (Covaris). Chromatin and antibody were mixed and rotated overnight at 4 °C. Antibodies were described above. Dynabeads M280 Sheep anti-mouse IgG or Dynabeads Protein G (Thermo Fisher Scientific) were used for immunoprecipitation. Beads were washed, and chromatin was eluted by ChlP direct elution buffer. The resulting chromatin was incubated overnight at 65 °C to reverse crosslinking. After digesting RNase and Proteinase K, ChIP DNA was purified with the Monarch PCR & DNA Cleanup Kit (NEB). Libraries were prepared using ThruPLEX DNA-seq Kit (Rubicon Genomics) according to the manufacturer’s instructions. Dual size selection was performed using Agencourt AMpure XP beads (Beckman Coulter). The libraries were pooled and sequenced by Next-Seq 500 (Illumina). Two independent biological replicates were analyzed for each genotype.

Prior to mapping of reads onto the *Arabidopsis thaliana* TAIR10 genome, trimming, and filtering of reads were conducted. Bowtie^57^ (version 1.2.2) with -m 1 -best parameters was used to control multi-reads and SAM files were obtained. The SAM files were converted to sorted BAM files using SAMtools^58^ (version 1.10). The resulting BAM files were then converted to BED files through BEDTools^59^ (version 2.27.0). To extend the 5′ end of reads toward the 3′ direction, the slop function in the BEDTools was utilized. Mapping were performed on the NIG supercomputer at the ROIS National Institute of Genetics. The resulting reads were counted using the coverage function in the BEDTools. Hypermethylated regions of H3K27me3 and hypomethylated regions of H3K4me3 in *jmjq* compared to WT were identified using a fold enrichment threshold of 1.5 by R (Version 3.5.2). To visualize binding peaks, Integrative Genome Viewer^60^ (version 2.4.14) was used. ngs.plot was used to generate metablot and heatmap for ChIP-seq data^61^. The sequence data were deposited into the DNA Data Bank of Japan (DRA011879).

### Reporting summary

Further information on research design is available in the Nature Research Reporting Summary linked to this article.

## Supplementary information

Supplementary Information

Descriptions of Additional Supplementary Files

Supplementary Data 1

Supplementary Data 2

Supplementary Data 3

Supplementary Data 4

Supplementary Data 5

Supplementary Data 6

Supplementary Data 7

Supplementary Data 8

Supplementary Data 9

Supplementary Data 10

Supplementary Data 11

Supplementary Data 12

Supplementary Data 13

Supplementary Data 14

Reporting Summary

## Data Availability

Data supporting the findings of this work are available within the paper and its Supplementary Information files. The datasets, plant materials, and mathematica programing language-written source code file (nb format file) are available from corresponding authors upon request. The RNA-seq (DRA008818, DRA009425) and ChIP-seq (DRA011879) data have been deposited in the DDBJ database (DRA008818, DRA009425, DRA011879). Source Data files are available in the online version of the paper. Source data are provided with this paper.

## Source data

Source Data
