# Supplementary material for: H3K27me3 demethylases alter HSP22 and HSP17.6C expression in response to recurring heat in Arabidopsis
Source: Nat Commun. 2021 Jun 9;12:3480. doi: 10.1038/s41467-021-23766-w (PMC8190089; doi:10.1038/s41467-021-23766-w)
Supplement: Supplementary file 1 — Supplementary Information [file 41467_2021_23766_MOESM1_ESM.pdf]

Supplementary Information for

H3K27me3 demethylases alter *HSP22* and *HSP17.6C* expression in response to recurring heat in *Arabidopsis*

Authors: Nobutoshi Yamaguchi[1,2*], Satoshi Matsubara[1], Kaori Yoshimizu[1], Motohide Seki[3], Kouta Hamada[4], Mari Kamitani[5], Yuko Kurita[5], Yasuyuki Nomura[5], Kota Nagashima[1], Soichi Inagaki[2,6], Takamasa Suzuki[7], Eng-Seng Gan[8], Taiko To[6], Tetsuji Kakutani[6,9,10], Atsushi J. Nagano[5,9], Akiko Satake[4], Toshiro Ito[1*]

Correspondence to: nobuy@bs.naist.jp; itot@bs.naist.jp

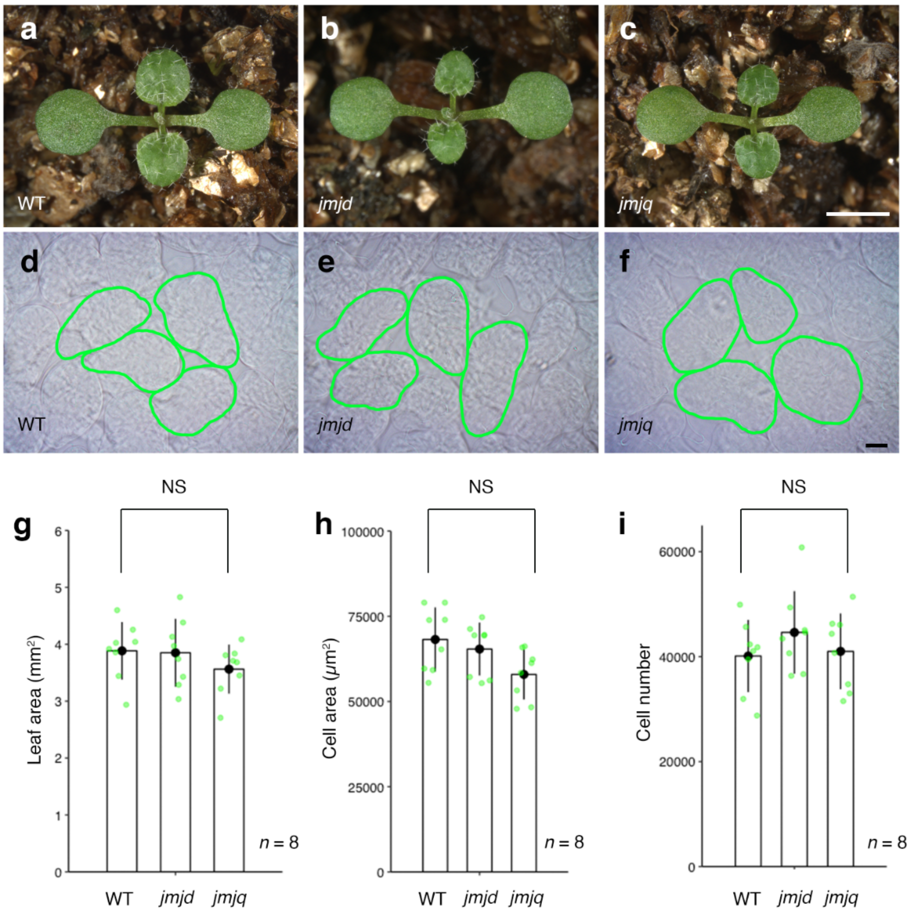

Supplementary Fig. 1 Leaf phenotypes in 10-day-old wild-type, *jmjd*, and *jmjq* seedlings. a–**c**, Seedlings of the wild type (a) and *jmjd* (b) and *jmjq* mutants (c) at 10 days after germination. At least, three independent experiments were performed and similar results were obtained. Bar = 1 mm. **d**–**f**, Palisade cells in the cotyledons of wild type (d), *jmjd* (e), and *jmjq* (f). Representative cells are marked in green. At least, three independent experiments were performed and similar results were obtained. Bar = 20 µm. **g**–**i**, Leaf area (g), cell area (h), and projected subepidermal palisade cell number (i) of wild type, *jmjd*, and *jmjq* at 10 days after germination. NS, nonsignificant based on one-way ANOVA test. $p < 0.05$. No significant differences in leaf area, cell area, or cell number were observed in these three mutants grown under control condition. These result suggest that acclimation defects seen in the *jmjq* mutant are not caused by morphological changes.

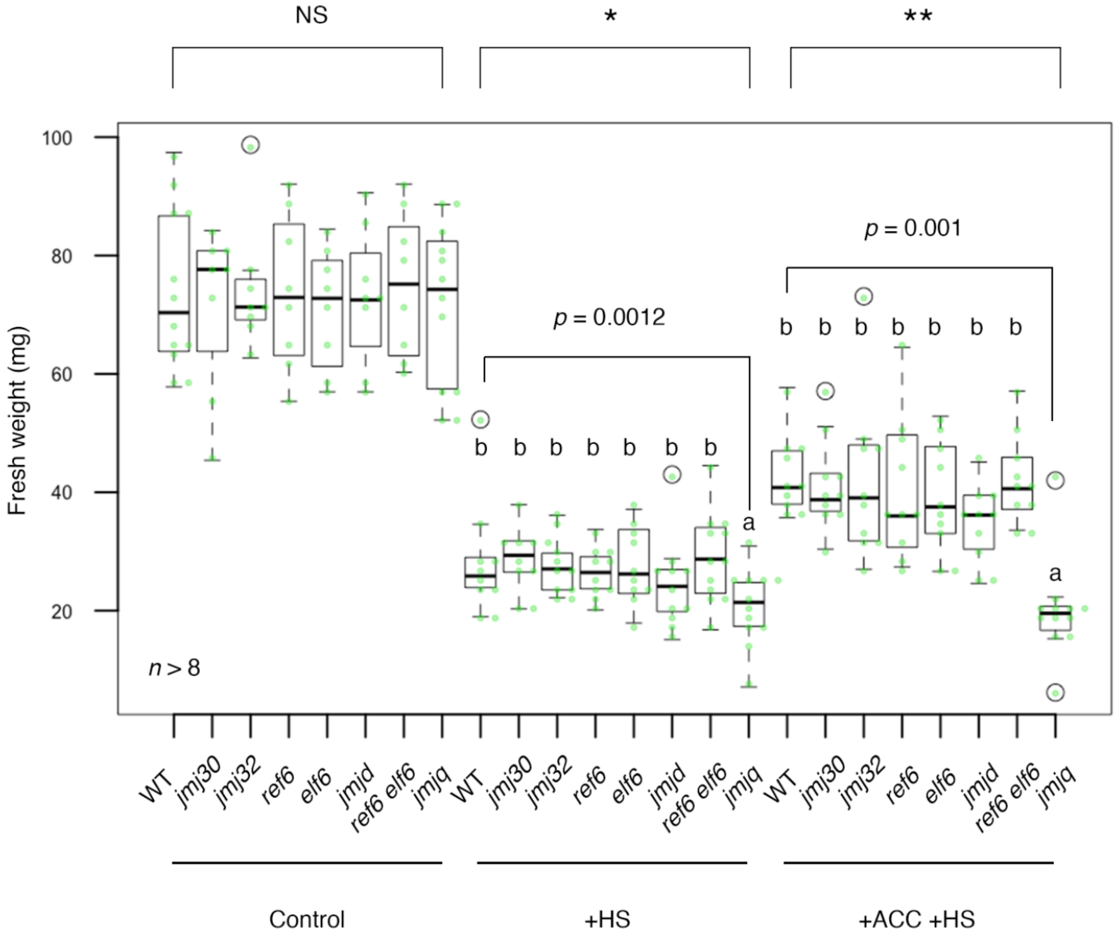

Supplementary Fig. 2 Heat acclimation phenotype in *jmjq* mutants. Quantification of seedling fresh weights. Sample minimum (lower bar); lower quartile (box); median (middle line); upper quartile (box); sample maximum (upper bar). Light green jitter dots and white circles represent the fresh weight from each sample and from statistical outliers, respectively. Asterisks indicate significant differences based on one-way ANOVA test. $*p < 1.0 \times 10^{-3}$, $p < 1.0 \times 10^{-4}$. Different letters indicate significant differences, while the same letters indicate non-significant differences based on post-hoc Tukey's HSD test. *p*-values based on post-hoc Tukey's HSD test between wild type and *jmjq* mutants are shown. $p < 0.05$. NS, nonsignificant.

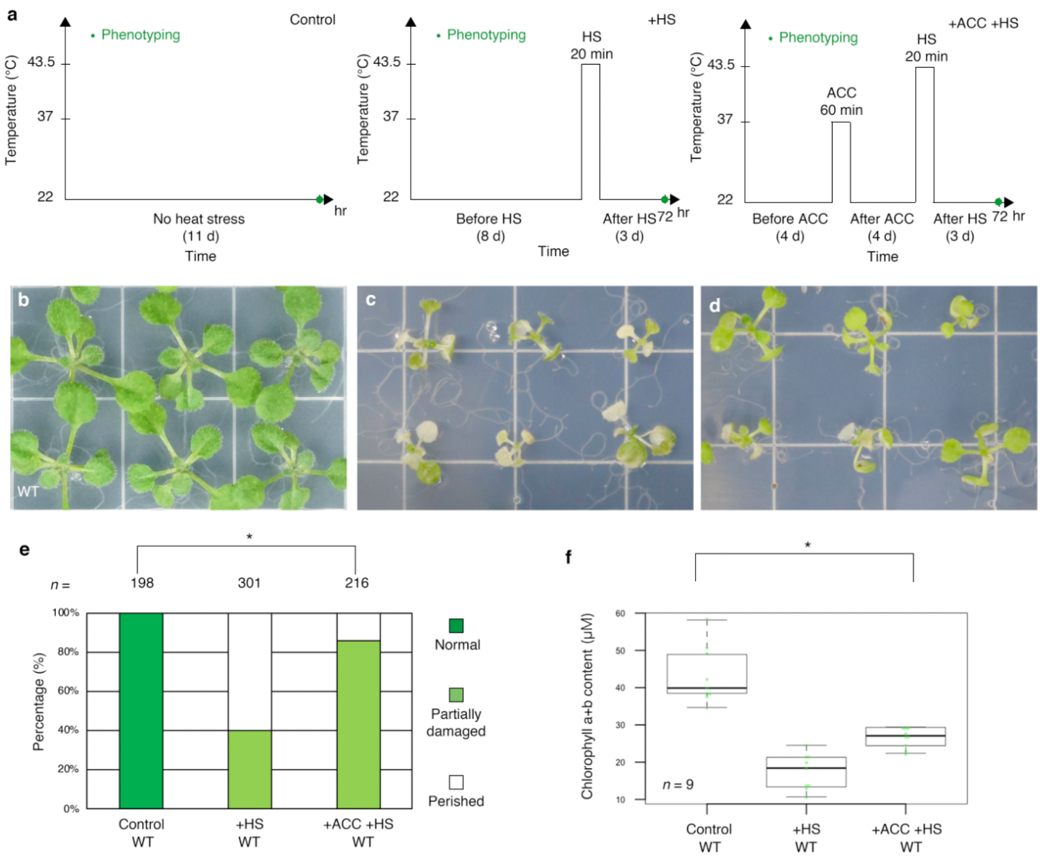

Supplementary Fig. 3 Heat acclimation phenotype in the wild type under different conditions. a, Schematic representation of the temperature conditions used. Phenotype, green. Left, normal plant growth conditions. Center, basal thermotolerance conditions. Right, heat-stress memory conditions. **b-d**, Wild type grown under control (b), +HS (c), and +ACC +HS (d) conditions. **e,** Quantification of survival rate. Seedlings grown under the two different heat-stress memory conditions were categorized into three groups based on phenotypic severity: green, normal growth; light green, partially damaged; white, perished. Significance was determined by χ^2 test followed by post-hoc test. $n > 197$. **f**, Quantification of chlorophyll contents. Sample minimum (lower bar); lower quartile (box); median (middle line); upper quartile (box); sample maximum (upper bar). Light green jitter dots represent the chlorophyll contents from each sample and from statistical outliers, respectively. One-way ANOVA test, $*p < 0.05$. Different letters indicate significant differences, while the same letters indicate

non-significant differences based on post-hoc Tukey's HSD test. $p < 0.05$. NS, nonsignificant. $n = 9$.

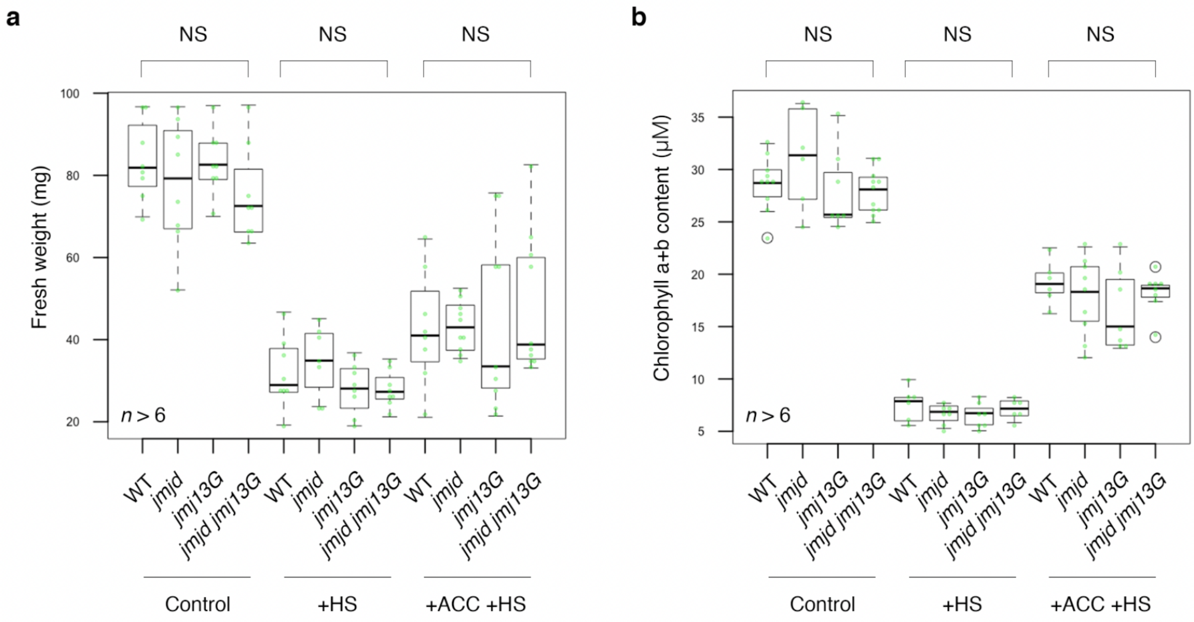

Supplementary Fig. 4 Heat acclimation phenotype of *jmj30 jmj32 jmj13G* triple mutants.

a, Quantification of seedling fresh weights. Sample minimum (lower bar); lower quartile (box); median (middle line); upper quartile (box); sample maximum (upper bar). Light green jitter dots and white circles represent the fresh weight from each sample and from statistical outliers, respectively. Asterisks indicate significant differences based on one-way ANOVA test. NS, nonsignificant. *n* > 6. **b**, Quantification of chlorophyll contents. Sample minimum (lower bar); lower quartile (box); median (middle line); upper quartile (box); sample maximum (upper bar). Light green jitter dots and white circles represent the chlorophyll contents from each sample and from statistical outliers, respectively. One-way ANOVA test. NS, nonsignificant. *n* > 6.

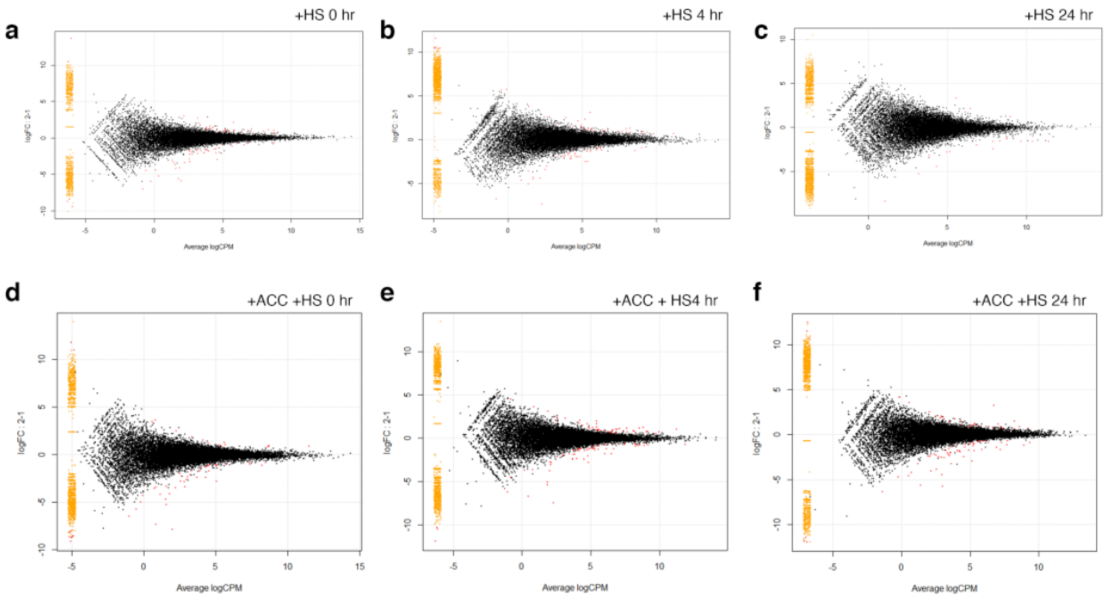

Supplementary Fig. 5 MA plots of the log fold change of all genes. a–f, RNA-seq results under basal thermotolerance condition (a-c) and heat-stress memory condition (d-f). Timing for RNA-seq, is shown in Fig 2a. The MA plots represent each gene with a dot. The *x* axis is the average log CPM over all genes; the *y* axis is the $\log_2$ fold change of normalized count between wild type and *jmjq* mutants. Genes with FDR <0.05 are shown in red. +HS 0 h (a), +HS 4 h (b), +HS 24 h (c), +ACC +HS 0 h (d), +ACC +HS 4 h (e), and +ACC +HS 24 h (f).

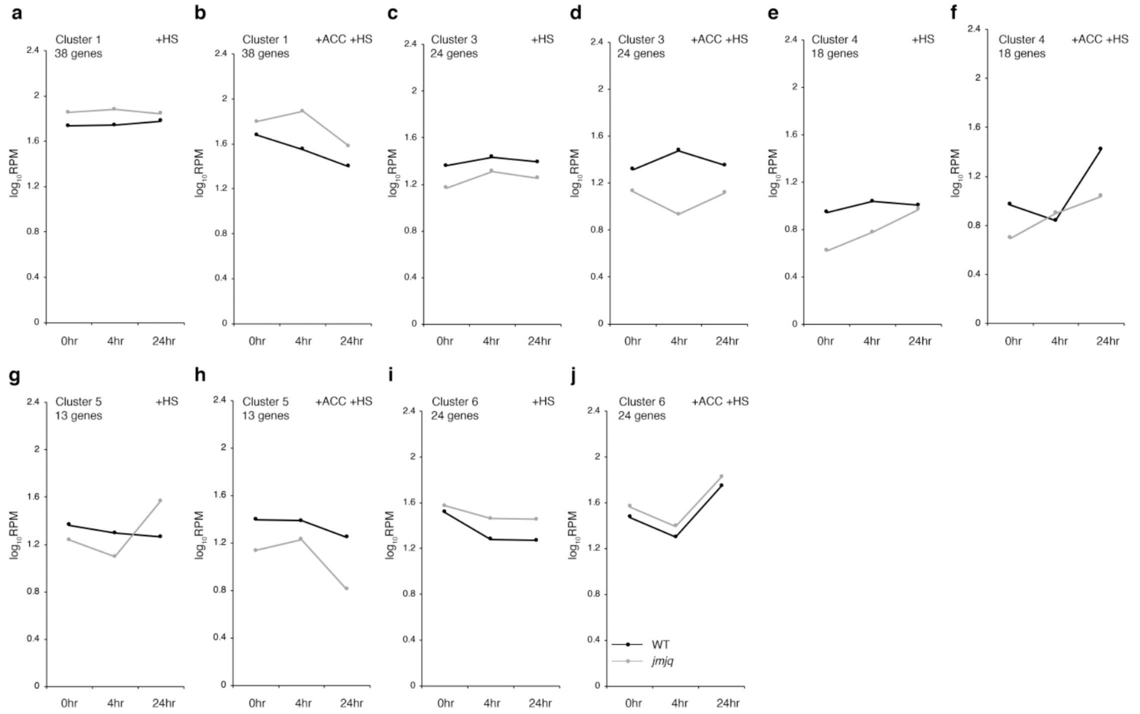

Supplementary Fig. 6 Gene expression of from the six hierarchical clusters. a–j, *k*-means clustering of genes differentially expressed between wild type and *jmjq* mutants. An optimal number of six clusters were identified using 142 differentially expressed genes in *jmjq* mutants grown under +ACC +HS conditions. Shown are cluster 1 +HS (a), cluster 1 +ACC +HS (b), cluster 3 +HS (c), cluster 3 +ACC +HS (d), cluster 4 +HS (e), cluster 4 +ACC +HS (f), cluster 5 +HS (g), cluster 5 +ACC +HS (h), cluster 6 +HS (i), and cluster 1 +ACC +HS (j). Two of these clusters comprise genes upregulated in the *jmjq* mutant: clusters 1 and 6 (62 genes). Four clusters comprise genes downregulated in the *jmjq* mutant: clusters 2, 3, 4, and 5 (80 genes). The graphs of cluster 2 are shown in Fig. 2. Since JMJs activate gene expression though demethylation of H3K27me3, 80 genes that were downregulated in the *jmjq* mutant were analyzed further.

a

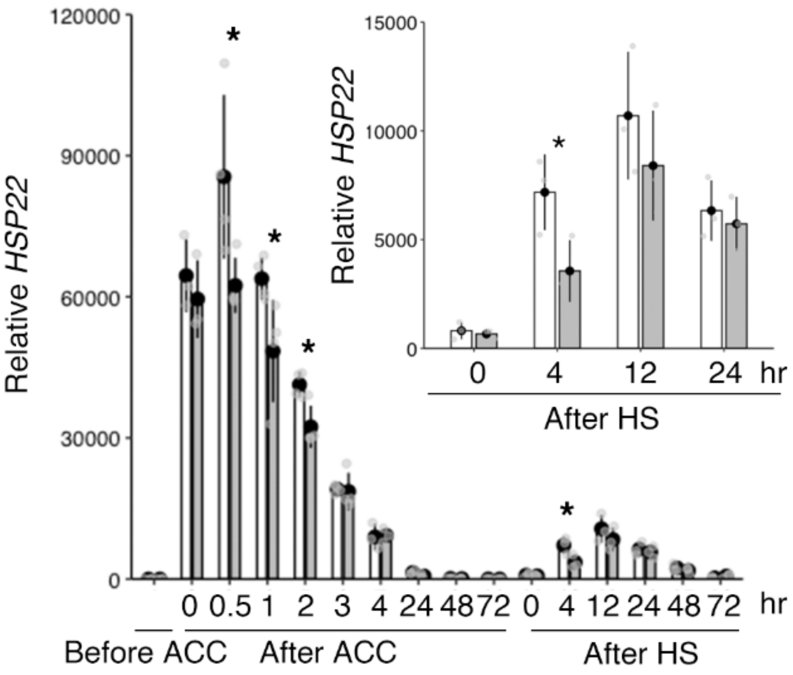

Relative HSP22
120000
90000
60000
30000
0
0 0.5 1 2 3 4 24 48 72 0 4 12 24 48 72 hr
Before ACC
After ACC
After HS
15000
10000
5000
0
0 4 12 24 hr
After HS

b

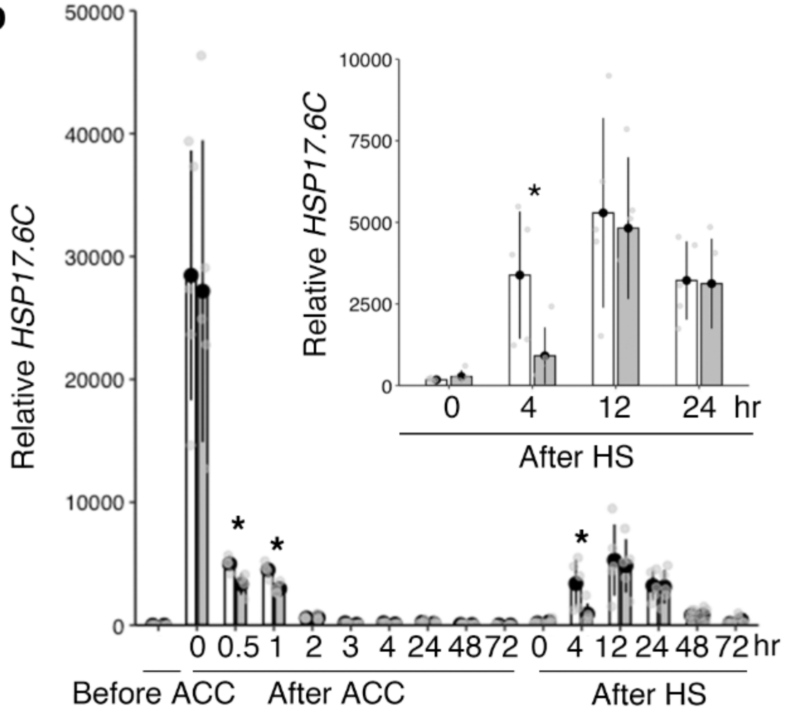

Relative HSP17.6C
50000
40000
30000
20000
10000
0
0 0.5 1 2 3 4 24 48 72 0 4 12 24 48 72 hr
Before ACC
After ACC
After HS
10000
7500
5000
2500
0
0 4 12 24 hr
After HS

Supplementary Fig. 7 *HSP22* and *HSP17.6C* expression in plants subjected to acclimation and heat shock.

a and **b**, Close-up view of Fig. 2f and g. qRT-PCR verification of *HSP22* (a) and *HSP17.6C* (b) expression in the wild type and *jmjq* mutant grown under the conditions shown in Fig. 2a. Gray jitter dots represent expression level from each sample. Asterisks indicate significant difference at the 0.05 level between the wild type and *jmjq* mutants at the same time point based on a two-tailed Student's *t*-test.

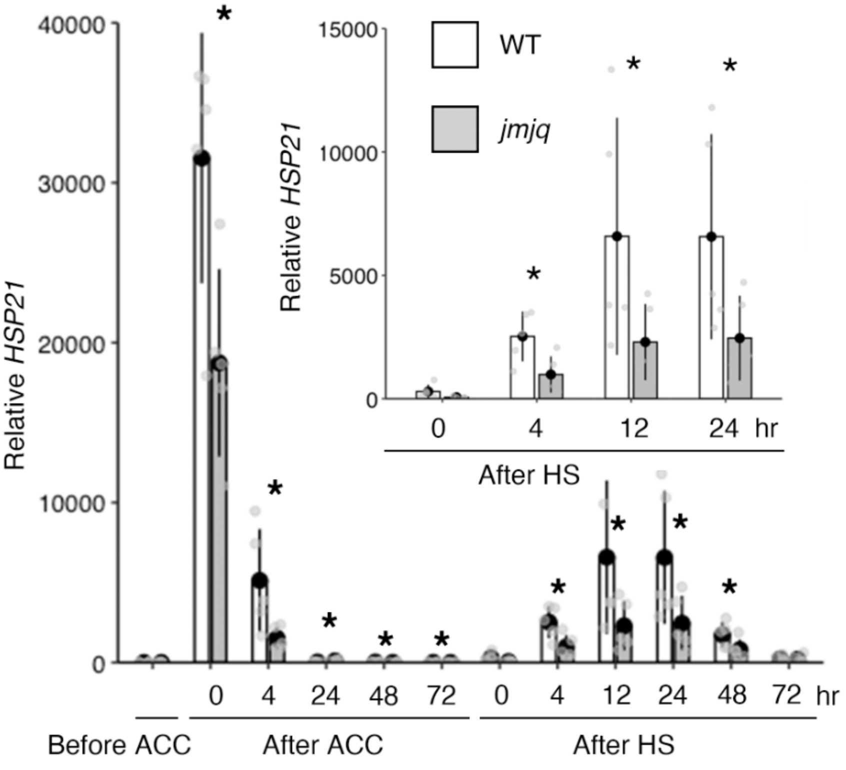

Supplementary Fig. 8 *HSP21* expression in plants subjected to acclimation and heat shock. qRT-PCR verification of *HSP21* expression in the wild type and *jmjq* mutant grown under the conditions shown in Fig. 2a. Gray jitter dots represent expression level from each sample. Asterisks indicate significant difference at the 0.05 level between the wild type and *jmjq* mutants at the same time point based on a two-tailed Student's *t*-test. *HSP21* expression after HS was reduced in acclimatized *jmjq* mutants compared to acclimatized wild-type plants.

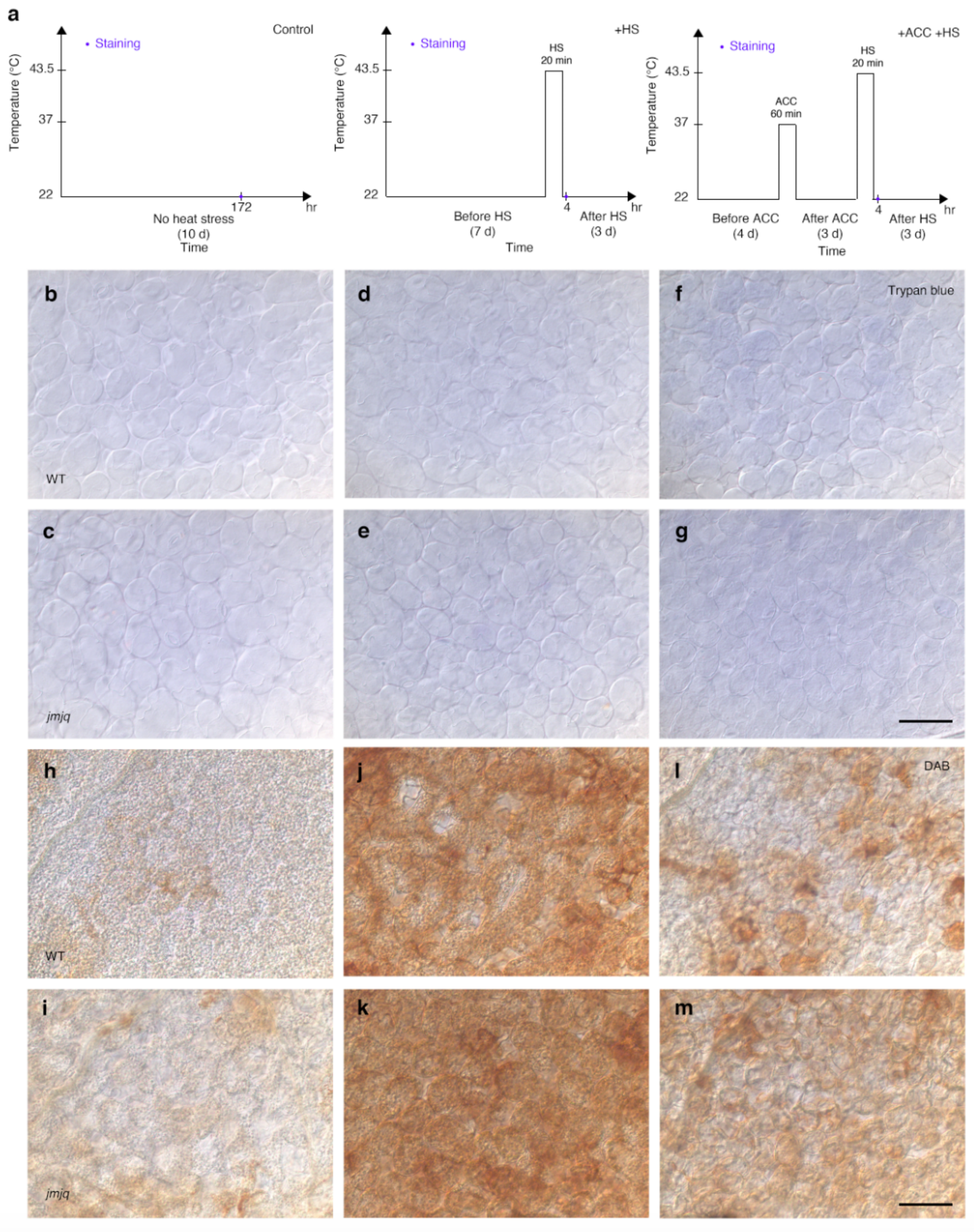

Supplementary Fig. 9 Cell death and ROS accumulation after heat shock. **a**, Schematic representation of temperature conditions. Staining (navy dots) was conducted at 7 days after germination. Left, normal plant growth condition. Center, basal thermotolerance condition. **b–m**, Trypan blue staining for the detection of cell death. (b and c) Wild type (b) and *jmjq* (c) grown under control

condition. (d and e) Wild type (d) and *jmjq* (e) grown under +HS condition. (f and g) Wild type (f) and *jmjq* (g) grown under +ACC +HS condition. (h–m) DAB staining for the detection of H_2O_2 production. (h, i) Wild type (h) and *jmjq* (i) grown under control condition. (j and k) Wild type (j) and *jmjq* (k) grown under +HS condition. (l and m) Wild type (l) and *jmjq* (m) grown under +ACC +HS condition. Scale bars = 100 μm. The majority of cells are damaged after heat shock treatment. However, cells remained alive under all conditions at the time point shown in Supplementary Fig. 9a. At least, three independent experiments were performed and similar results were obtained. These result suggest that the lack of expression of *HSP* genes right after the heat shock could be due to damage of cells.

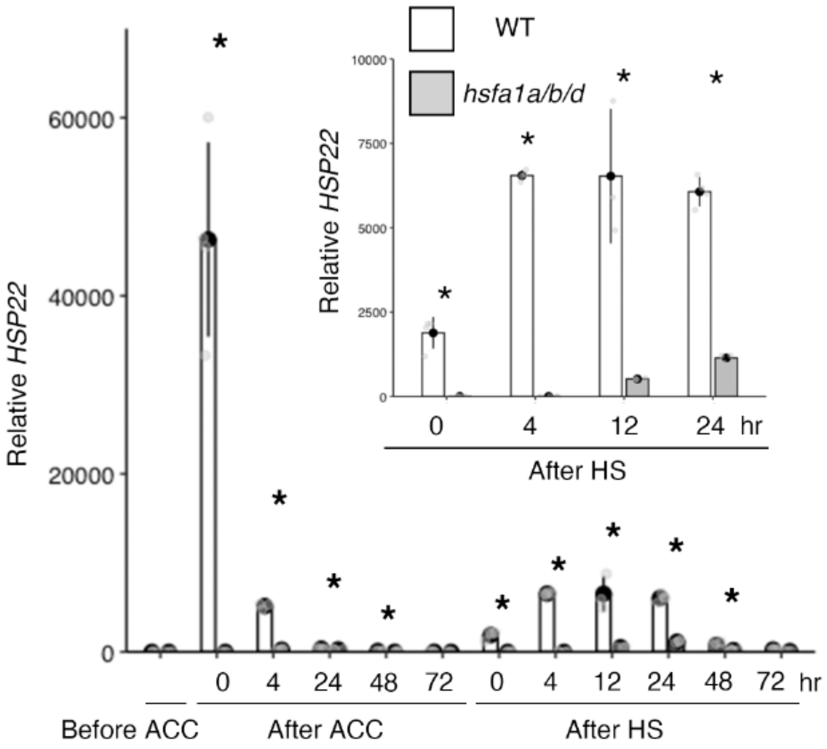
WT
hsfa1a/b/d
Relative HSP22
After HS
Before ACC
After ACC
After HS
0 4 24 48 72 0 4 12 24 48 72 hr

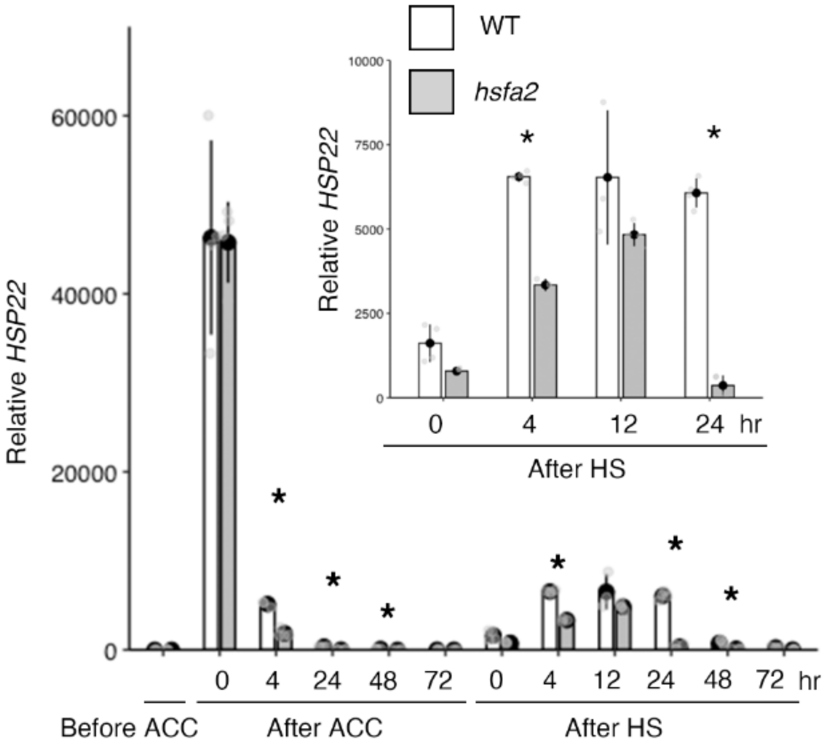
WT
hsfa2
Relative HSP22
After HS
Before ACC
After ACC
After HS
0 4 24 48 72 0 4 12 24 48 72 hr

Supplementary Fig. 10 Expression of *HSP* genes with acclimation and heat shock in *hsfa1* and *hsfa2* mutants.

qRT-PCR verification of the *HSP22* gene in the wild type, *hsfa1* (above), and *hsfa2* (below) mutants grown under the conditions shown in Fig. 2a. Gray jitter dots represent expression level from each sample. Asterisks indicate significant difference at 0.05 levels between the wild type and *hsfa* mutants at the same time point based on a two-tailed Student's *t*-test.

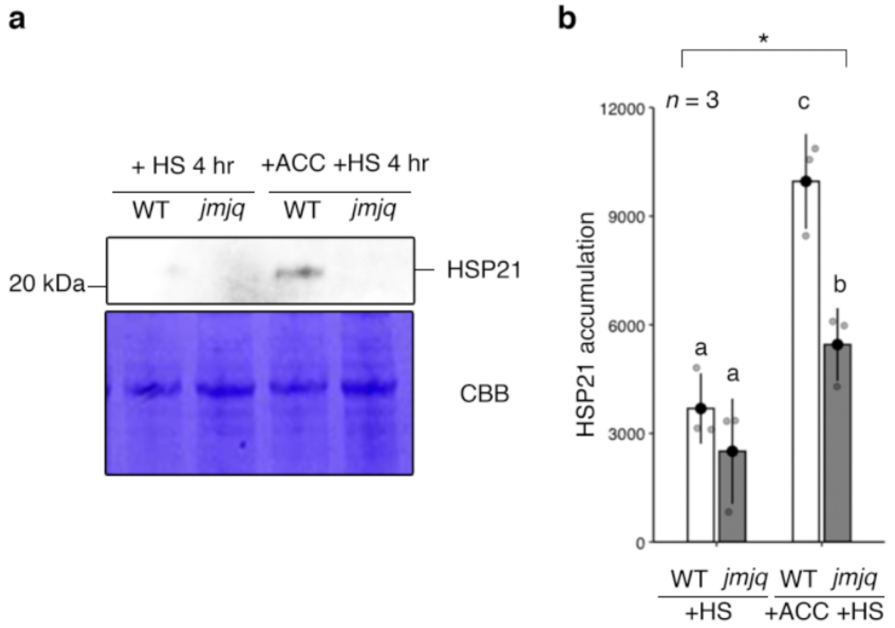

Supplementary Fig. 11 HSP21 accumulation in the wild type and *jmjq* mutants.

a and **b**, Immunoblotting of HSP21 in wild type and *jmjq* mutants grown under conditions shown in Fig. 2a. (a) Immunoblot analysis of protein extracts using the HSP21 antibody. Coomassie Brilliant Blue-stained membranes (CBB) are shown as loading controls. Three independent experiments were performed and similar results were obtained. (b) Quantification of immunoblot signals. One-way ANOVA test, *$p < 0.05$. Different letters indicate significant differences, while the same letters indicate non-significant differences based on post-hoc Tukey's HSD test ($p < 0.05$). NS, nonsignificant. $n = 3$.

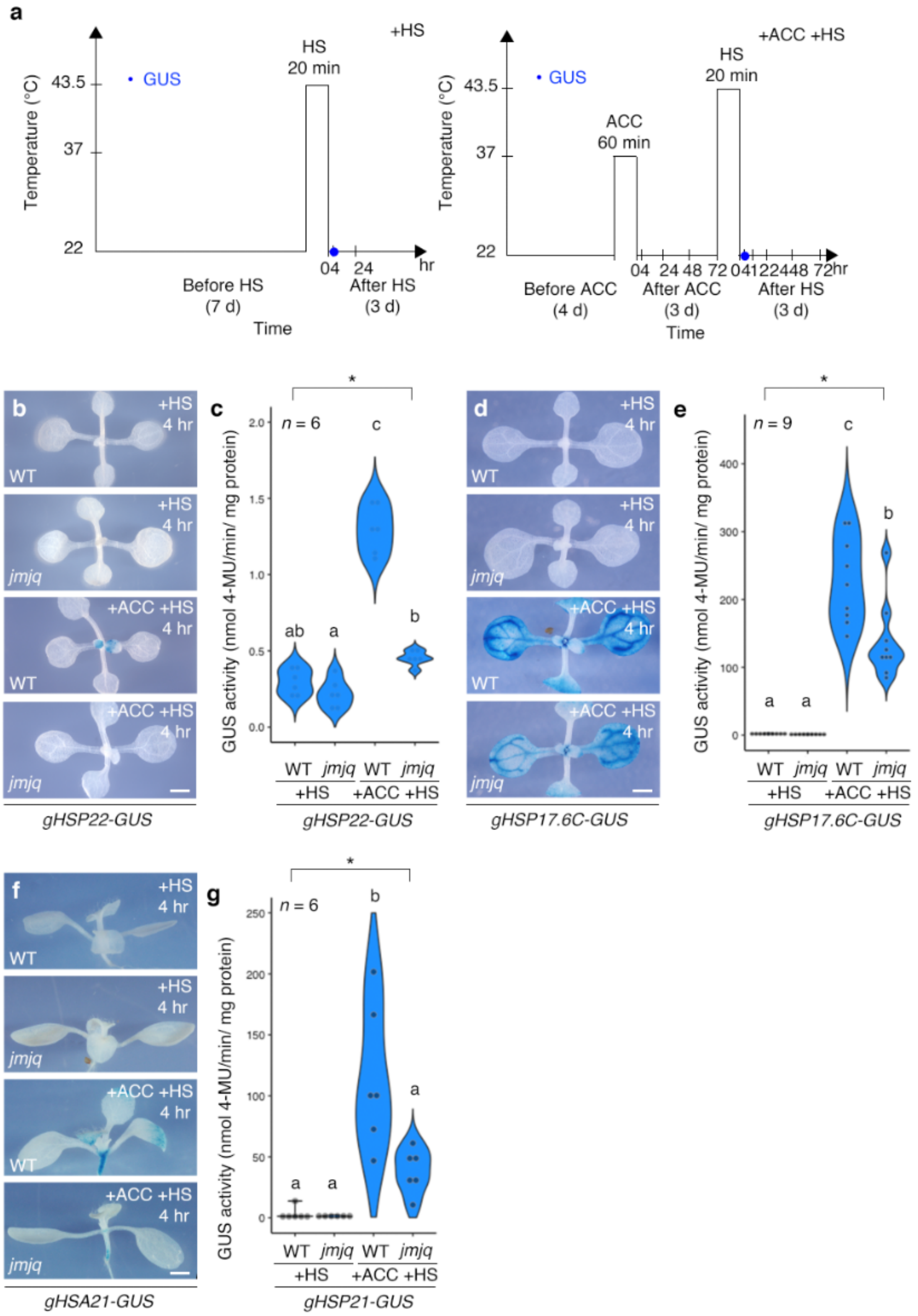

Supplementary Fig. 12 HSP22-GUS, HSP17.6C, and HSP21 accumulation in wild-type plants and *jmjq* mutants.

a, Schematic representation of the temperature conditions used. Left, basal thermotolerance condition; right, heat-stress memory condition. GUS, blue. **b-g**, gHSP22-GUS (b, c), gHSP17.6C-GUS (d, e), and gHSP21-GUS (f, g) expression in wild-type and *jmjq* mutant plants grown under the conditions shown in Supplementary Fig. 12a. Whole mount GUS staining of gHSP22-GUS (b), gHSP17.6C-GUS (d), and gHSP21-GUS (f). gHSP22-GUS, gHSP17.6C-GUS, and gHSP21-GUS were highly accumulated in acclimatized wild type plants after HS. HSP22 accumulation was not detected in acclimatized *jmjq* mutants 4h after HS treatment. Only faint HSP17.6C and HSP21 accumulation were detected in acclimatized *jmjq* mutants 4h after HS treatment. At least, three independent experiments were performed and similar results were obtained. Scale bar, 1 mm. Quantification of GUS activity by MUG assay in gHSP22-GUS (c), gHSP17.6C-GUS (e), and gHSP21-GUS (g). Asterisk indicates significant differences based on one-way ANOVA test (p < 0.05). Different letters indicate significant differences, while the same letters indicate non-significant differences based on post-hoc Tukey's HSD test (p < 0.05). NS, nonsignificant. n > 5.

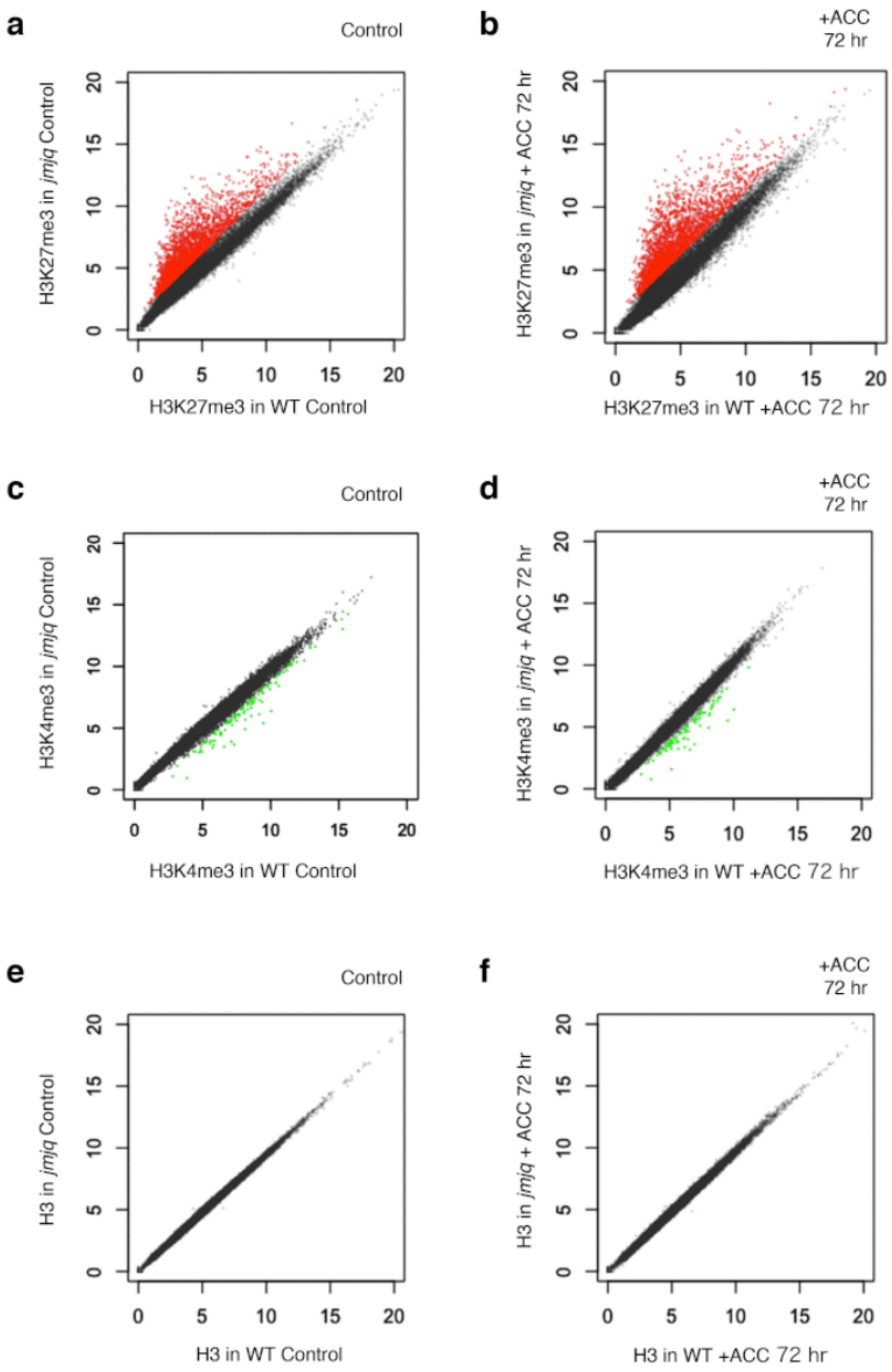

Supplementary Fig. 13 Scatter plot of H3K27me3, H3K4me3, and H3 ChIP-seq a and **b**, H3K27me3 levels determined by ChIP-seq in wild type and *jmjq* mutants without acclimation (a) and 3 days (72 h) after acclimation (b). **c** and **d**, H3K4me3 levels determined by ChIP-seq in wild type and *jmjq* mutants without

acclimation (c) and 3 days (72 h) after acclimation (d). **e** and **f**, H3 levels determined by ChIP-seq in wild type and *jmjq* mutants without acclimation (e) and 3 days (72 h) after acclimation (f). Each dot represents the square root of the read counts per million mapped reads (RPM). Genes that were hyper H3K27 trimethylated in the *jmjq* mutant and hyper H3K4 trimethylated in wild type are shown in red and light green, respectively. Consistent with the biochemical role of JMJ in the removal of H3K27me3, more than 2000 genes were hypermethylated in the *jmjq* mutant. Hundreds of transcription start sites of genes were hypertrimethylated by H3K4me3 in wild type under two different conditions. No differences in H3 levels were observed between wild type and *jmjq* mutants with and without acclimation.

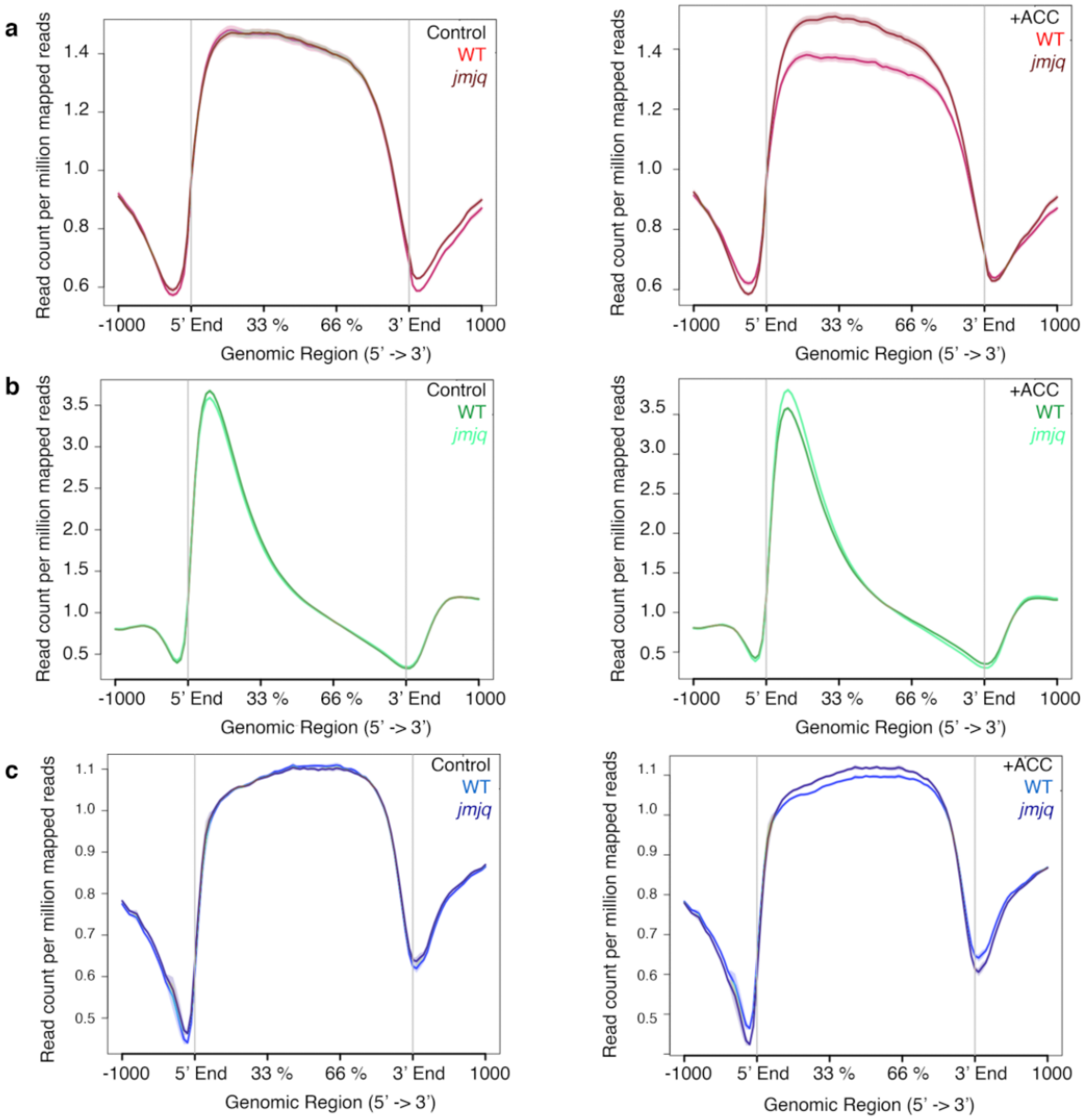

Supplementary Fig. 14 Averaged profiles of H3K27me3, H3K4me3, and H3 around genes. a–c, Metablots of H3K27me3 (a), H3K4me3 (b), and H3 (c) around genes in wild type and *jmjq* mutants without acclimation (left) and 3 days (72 h) after acclimation (right). Regardless of conditions or genotypes, H3K27me3 and H3K4me3 were observed in gene body and near transcription start sites. H3 enrichment in wild type and *jmjq* mutants with and without acclimation were also observed in gene bodies.

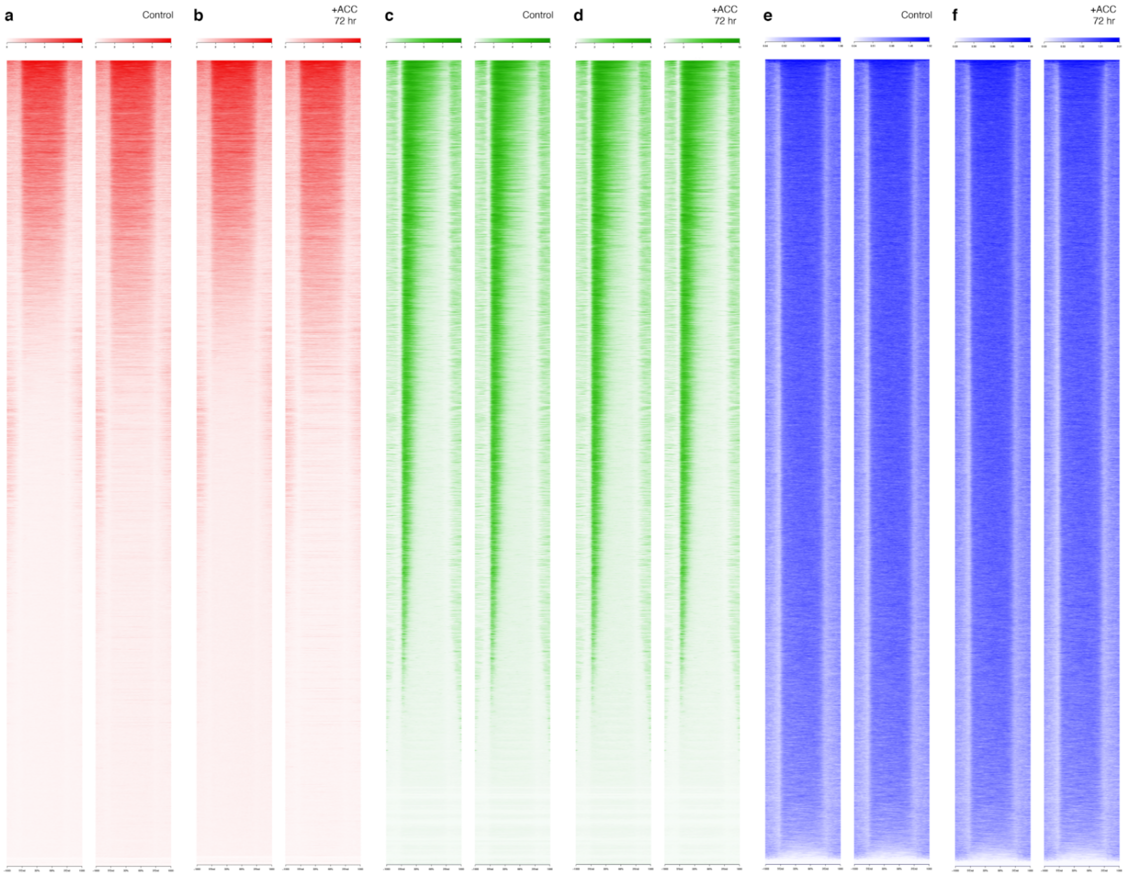

Supplementary Fig. 15 Heatmaps of H3K27me3, H3K4me3, and H3. a and **b**, Heatmaps of H3K27me3 around genes in wild type and *jmjq* mutants without acclimation (a) and 3 days (72 h) after acclimation (b). **c** and **d**, Heatmaps of H3K4me3 around genes in wild type and *jmjq* mutants without acclimation (c) and 3 days (72 h) after acclimation (d). **e** and **f**, Heatmaps of H3 around genes in wild type and *jmjq* mutants without acclimation (e) and 3 days (72 h) after acclimation (f).

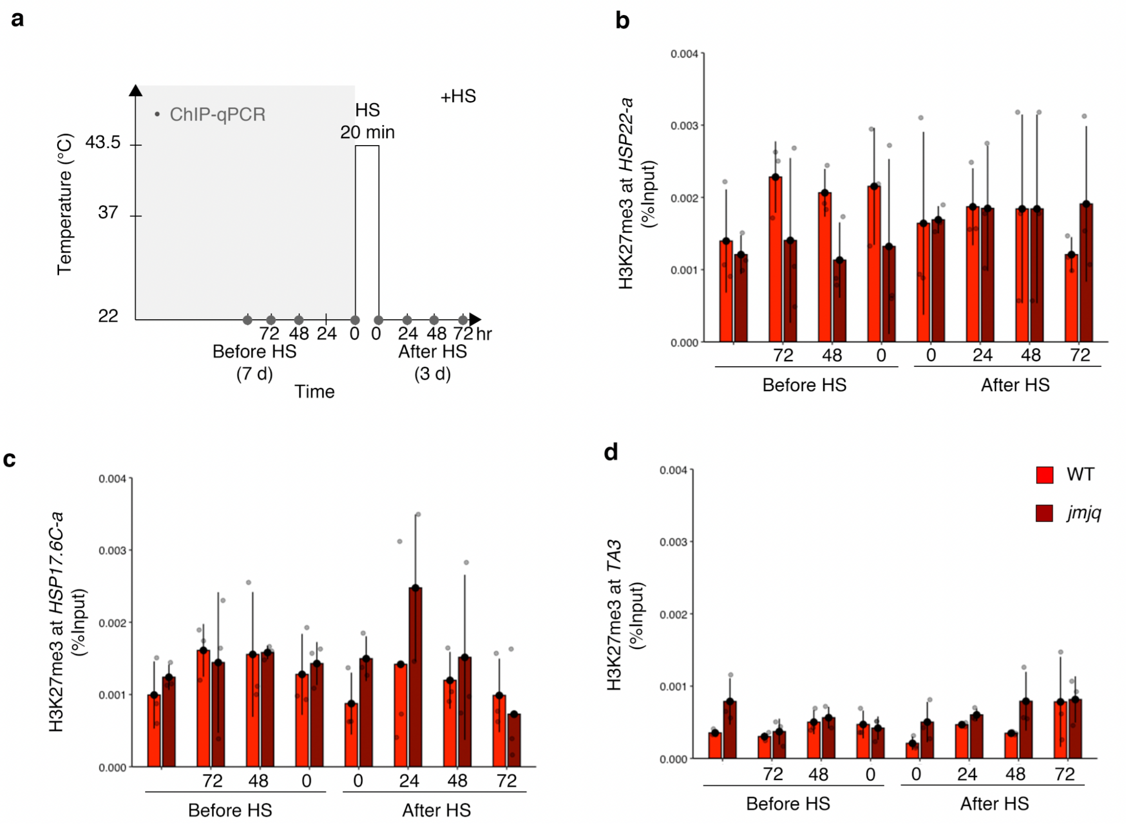

Supplementary Fig. 16 H3K27me3 levels at the *HSP22*, *HSP17.6C*, and *TA3* loci in the wild type and *jmjq* mutants under HS conditions. a, Schematic representation of the basal thermotolerance condition used. **b-d**, H3K27me3 levels at the *HSP22* (b), *HSP17.6C* (c), and *TA3* (d) loci in the wild type and *jmjq* mutants under the conditions shown in Supplementary Fig. 16a, as determined by ChIP-qPCR. Gray jitter dots represent expression level from each sample. n = 3.

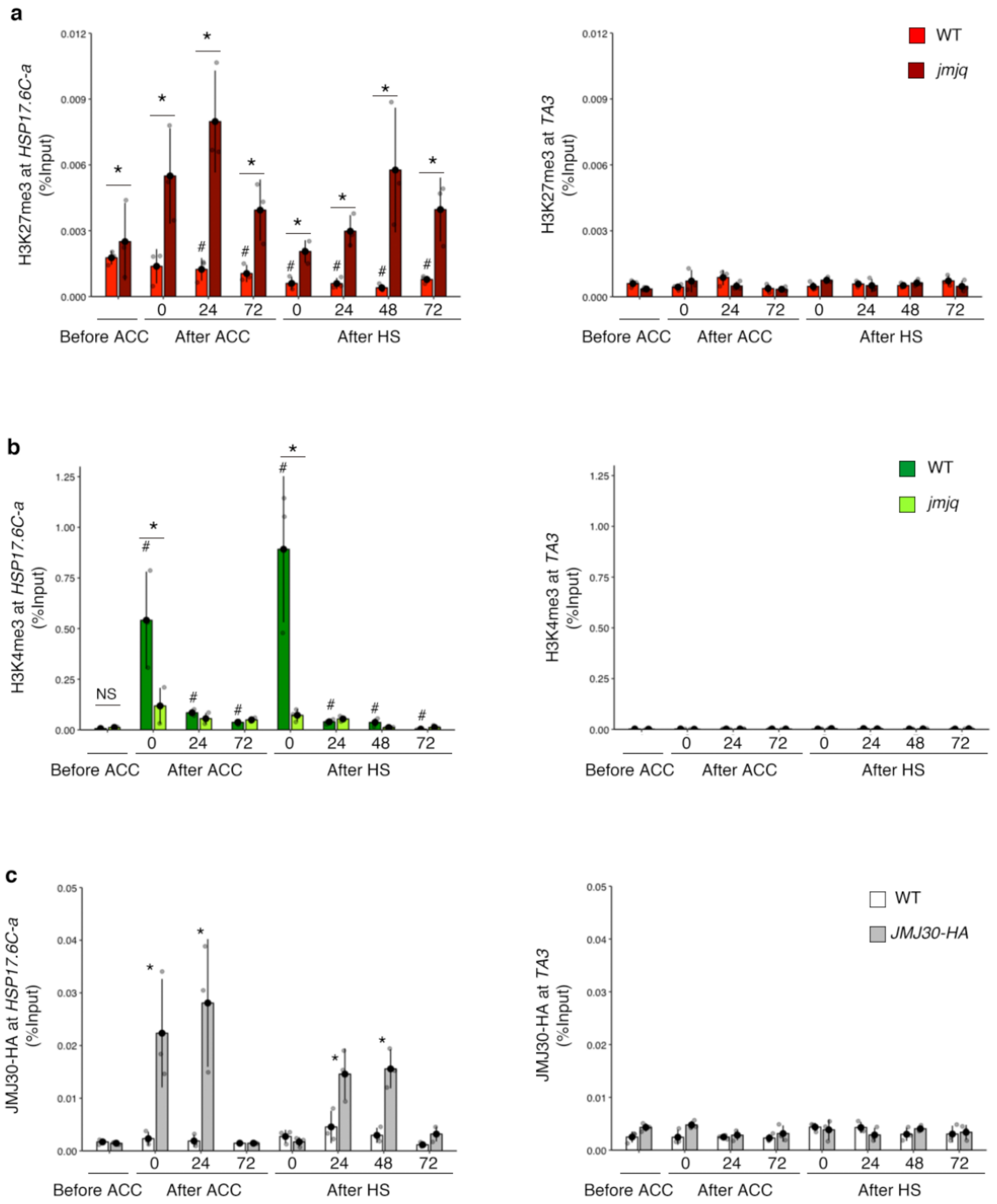

Supplementary Fig. 17 H3K27me3, H3K4me3, and JMJ30-HA levels at the *TA*3 locus in the wild type and *jmjq* mutants. a-c, H3K27me3 (a) and H3K4me3 (b) levels in the wild type and *jmjq* mutants and JMJ30-HA (c) levels in the wild type and the previously published biologically-functional JMJ30-HA line at *TA3* determined by ChIP-qPCR. Grey jitter dots represent expression level from each sample. Left: positive controls from Fig. 3 (the same graphs (Fig. 3d-f)) are shown for comparison). Right: no difference at the *TA3* locus was observed by a two-tailed Student's *t*-test.

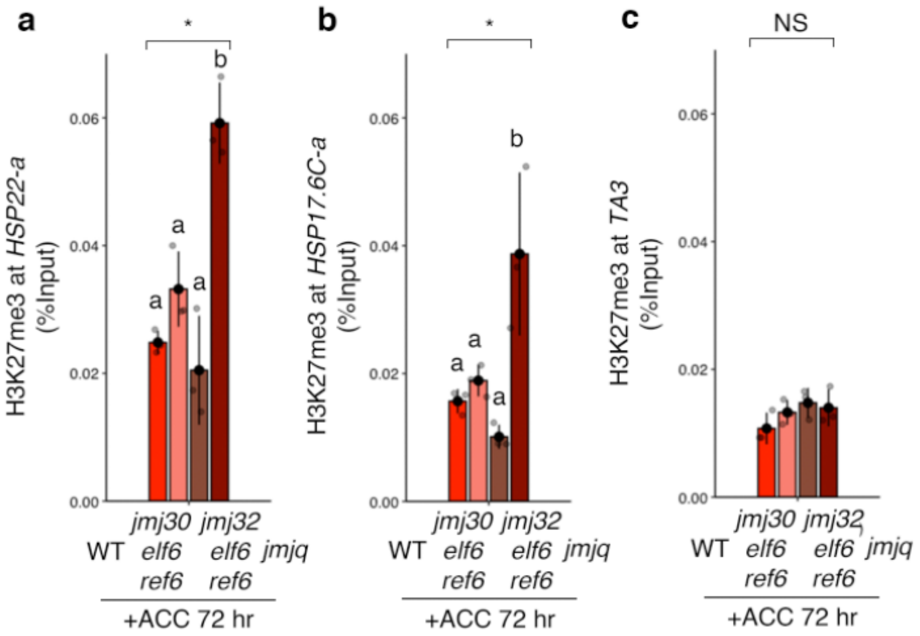

Supplementary Fig. 18 H3K27me3 levels at the *HSP22* and *HSP17.6C* loci in the wild type and *jmj* triple and *jmjq* mutants 3 days after ACC. a, **b**, H3K27me3 levels at *HSP22-a* (a) and *HSP17.6C-a* (b) in the wild type and *jmj30-2 elf6-3 ref6-1*, *jmj32-1 elf6-3 ref6-1*, and *jmjq* mutants after 3 days of ACC and in the corresponding non-acclimatized controls. One-way ANOVA test, *$p < 0.05$. Letters above bars indicate significant difference on post-hoc Tukey HSD test ($p < 0.05$).

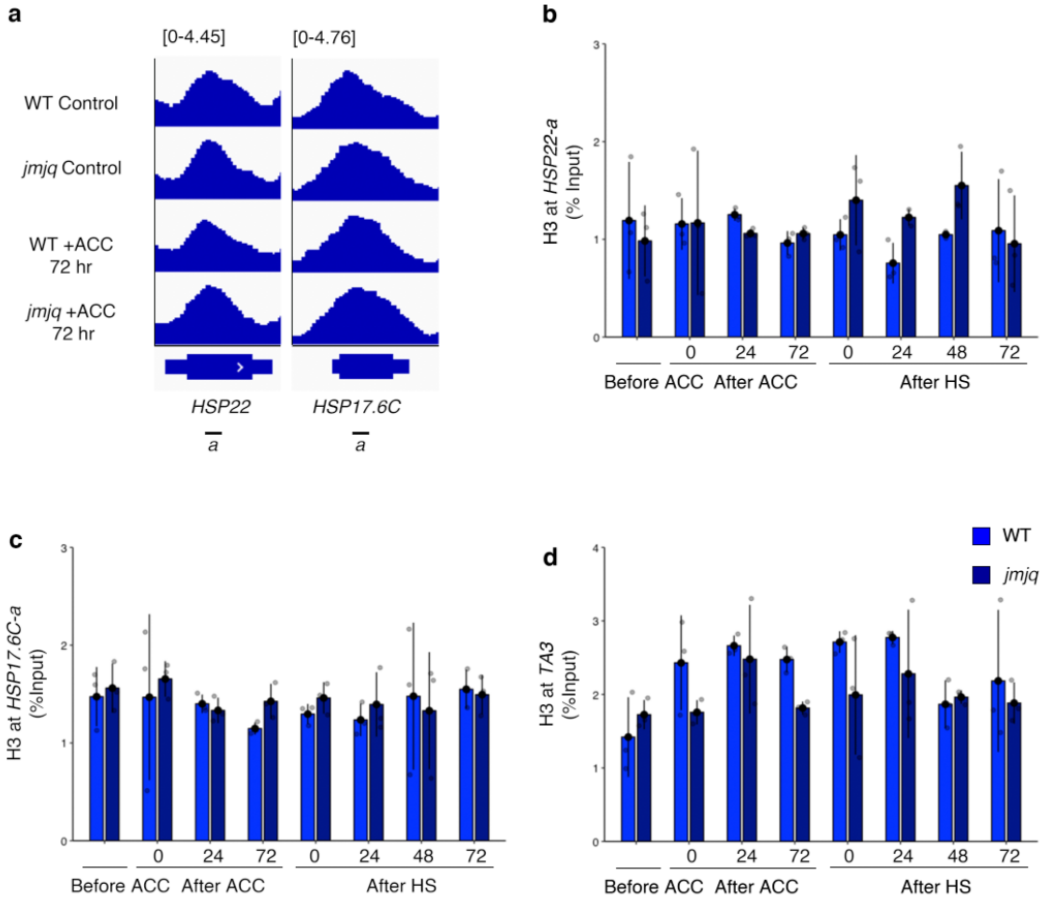

Supplementary Fig. 19 Histone H3 levels at the *HSP22* and *HSP17.6C* loci in the wild type and *jmjq* mutants. a, H3 peaks detected by ChIP-seq at the *HSP22* and *HSP17.6C* loci in the wild type and *jmjq* mutants without acclimation (above) and 3 days (72 h) after acclimation (below). **b** and **c**, Histone H3 levels at *HSP22* (b), *HSP17.6C* (c), and *TA3* (d) determined by ChIP-qPCR in the wild type and *jmjq* mutants. Grey jitter dots represent expression level from each sample. No difference in histone H3 signals at those two loci was observed between the wild type and *jmjq* mutants by a two-tailed Student's *t*-test.

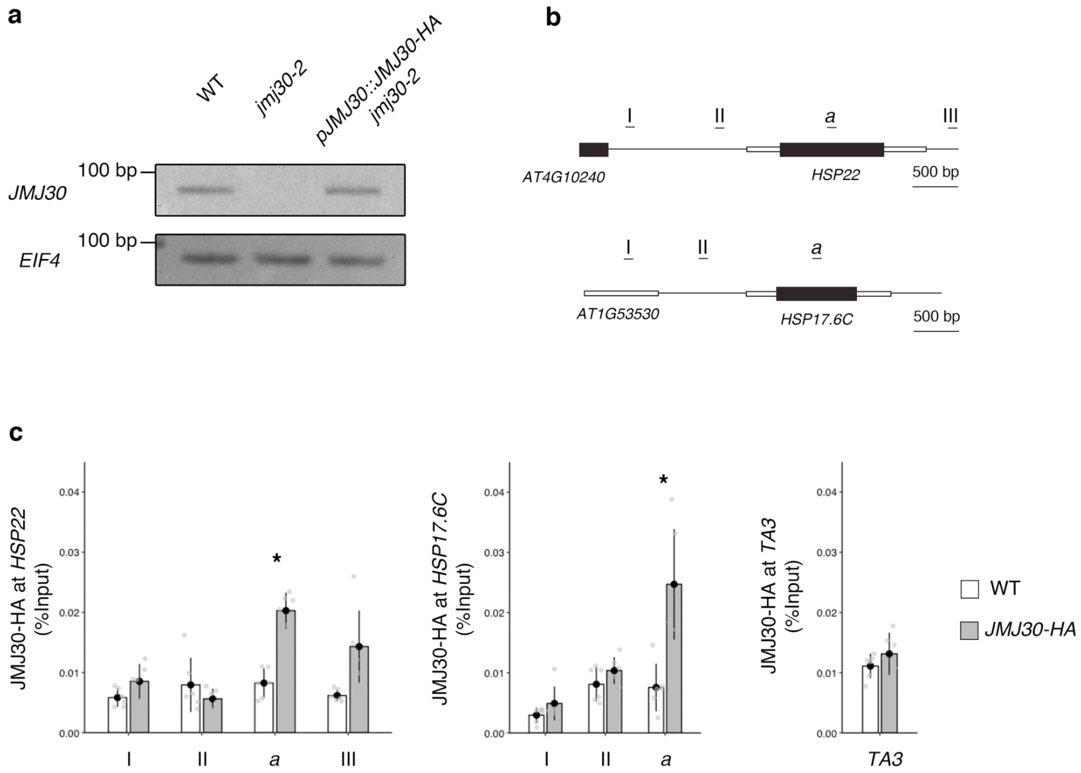

Supplementary Fig. 20 JMJ30 binding at the *HSP22* and *HSP17.6C* loci in response to ACC.

a, Expression of *JMJ30* (above) and *EIF4A1* (below) in wild-type, *jmj30-2*, and *pJMJ30::JMJ30-HA jmj30-2* seedlings. *EIF4A1* was used as a loading control. Primer sets for qRT-PCR were used. Three independent experiments were performed and similar results were obtained. **b**, Schematic diagram of *HSP22* (left) and *HSP17.6C* (right) amplicons indicated as letters I-III and *a* used for ChIP-qPCR. **c**, JMJ30 levels at *HSP22* (left), *HSP17.6C* (middle), and *TA3* (right) determined by ChIP-qPCR using wild type and *pJMJ30::JMJ30-HA jmj30-2* 3 days after ACC. Grey jitter dots represent expression level from each sample. Asterisks indicate significant difference at 0.05 levels between the wild type and *pJMJ30::JMJ30-HA jmj30-2* at the same position based on a two-tailed Student's *t*-test.

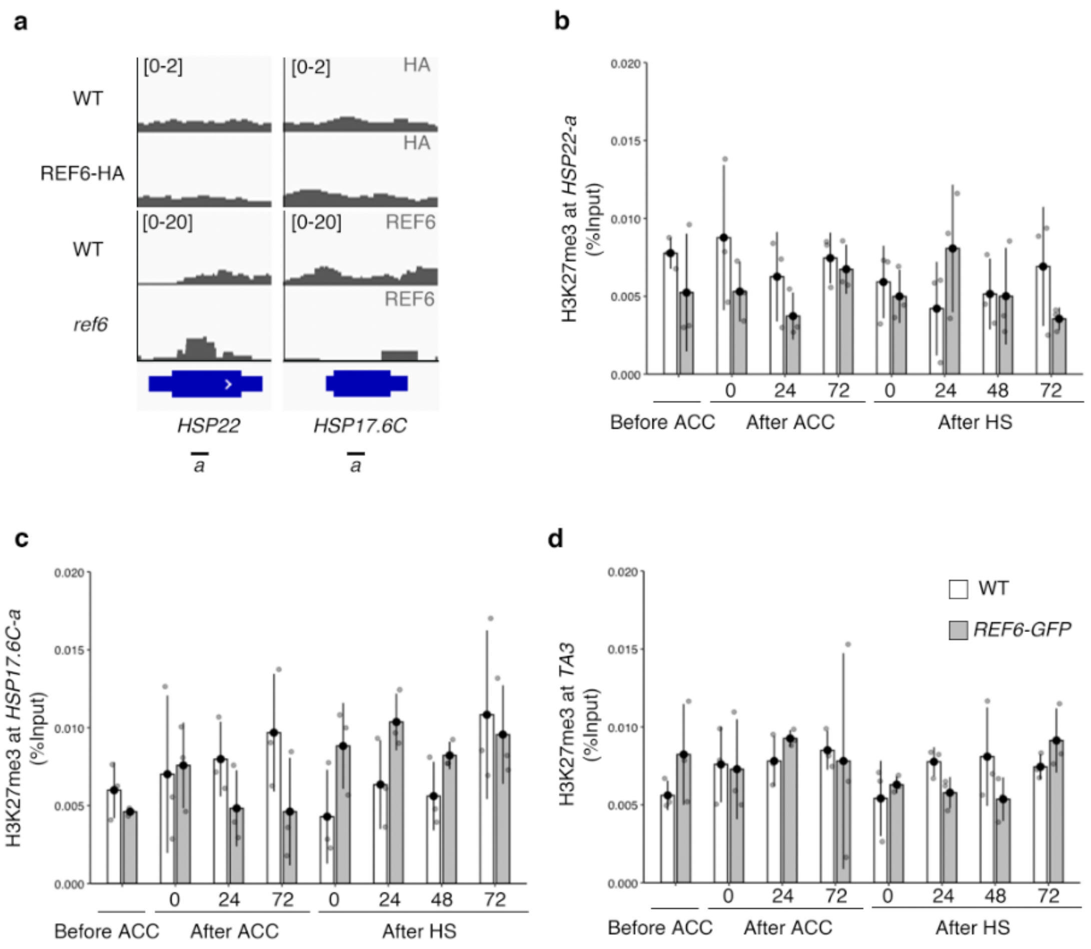

Supplementary Fig. 21 REF6 levels at the *HSP22* and *HSP17.6C* loci. a, REF6 binding peaks detected by ChIP-seq at the *HSP22* and *HSP17.6C* loci. Public REF6 data were used[23, 24]. Above: HA ab ChIP using the *gREF6::REF6-HA* transgenic line. Below: REF6 ab ChIP using WT and *ref6* mutants. **b-d**, REF6 levels at *HSP22* (b), *HSP17.6C* (c), and *TA3* (d) determined by ChIP-qPCR using the wild type and *gREF6::REF6-GFP*. Grey jitter dots represent expression level from each sample. No difference in histone REF6 signals at those three loci was observed between the wild type and *gREF6::REF6-GFP* by a two-tailed Student's *t*-test.

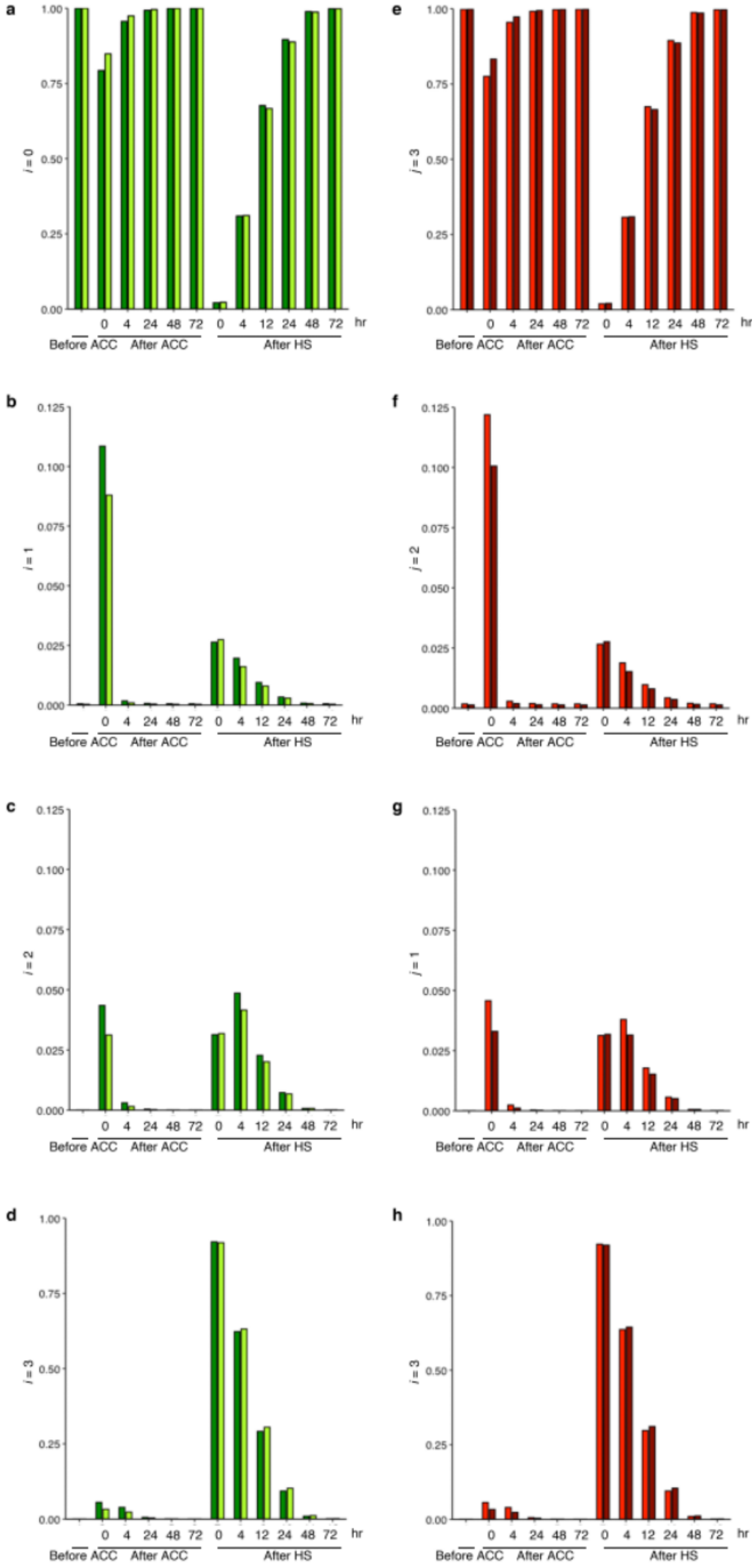

a
b
c
d
e
f
g
h
j = 0
j = 1
j = 2
j = 3
0 4 24 48 72 0 4 12 24 48 72 hr
Before ACC
After ACC
After HS

Supplementary Fig. 22 The memory effects of having multiple actively modified nucleosomes. **a**–**d**, Change in fraction of cells that have 0 (a), 1 (b), 2 (c), and 3 (d) actively modified nucleosomes. **e**–**h**, Change in fraction of cells that have 3 (e), 2 (f), 1 (g), and 0 (h) repressively modified nucleosomes. After ACC and HS, a considerable fraction of cells are suggested to stay at states with 1 or 2 actively modified nucleosomes. These results indicate the importance in the memory effect of having multiple modifiable sites.

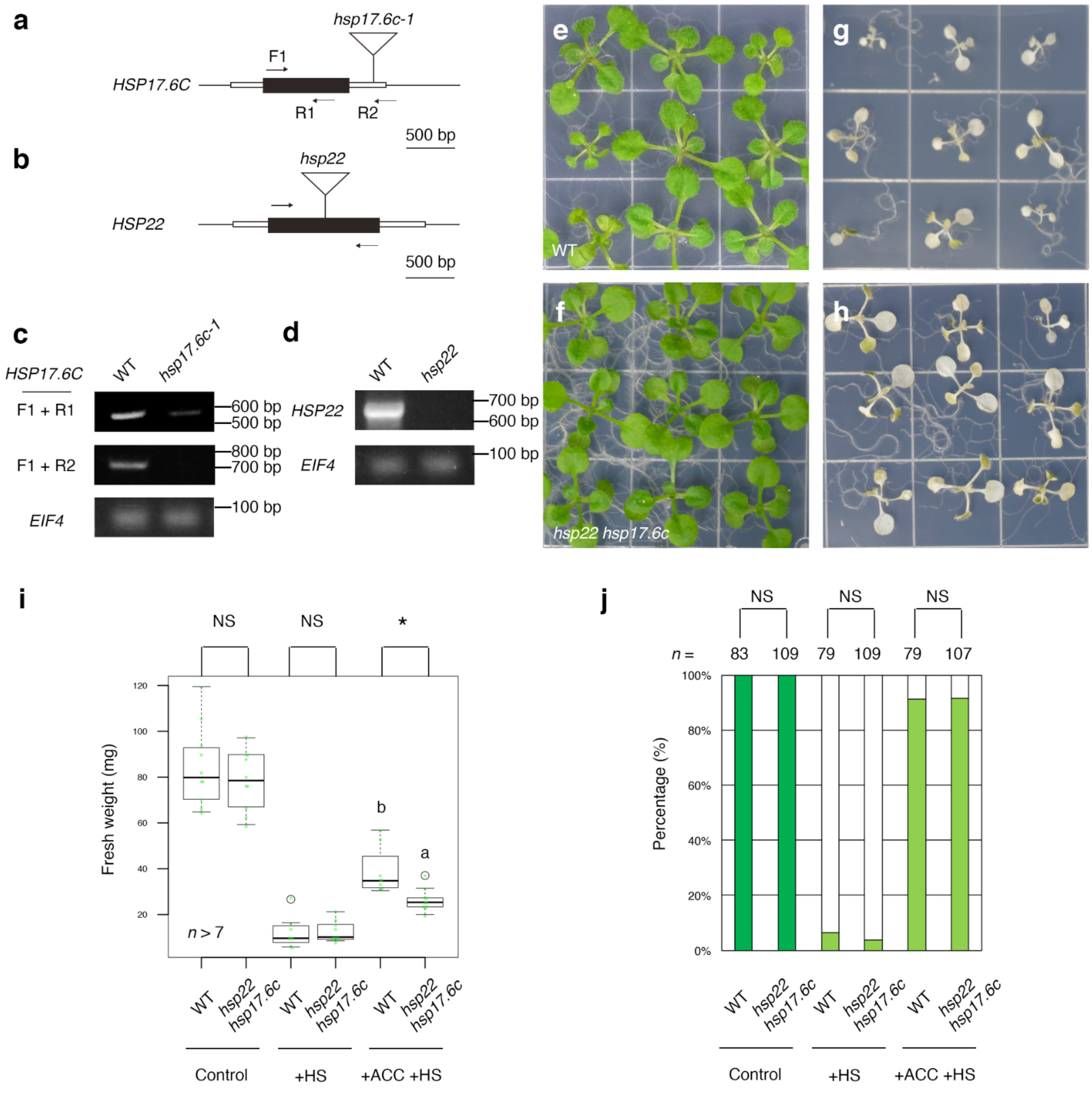

Supplementary Fig. 23 The *hsp22 hsp17.6c* double mutant showed decreased heat acclimation capacity. a and **b**, Schematic structure of *HSP17.6C* (a) and *HSP22* (b) genes and T-DNA insertions. Arrows indicate gene-specific primers used for genotyping. **c**, Expression of *HSP17.6C* (above) and *EIF4A1* (below) in wild-type and the previously identified *hsp17.6c-1* seedlings. *HSP17.6C* PCR was performed with primers upstream of the *hsp17.6c-1* insertion (F1+R1; primers F1 and R1 in a) or flanking the insertions (F1+R2; primers F1 and R2 in a). *EIF4A1* was used for a loading control. Three independent experiments were performed and similar results were obtained. **d**, Expression of *HSP22* (above) and *EIF4A1* (below) in wild-type and previously identified *hsp22* seedlings[18]. PCR amplifiaction of *HSP22* was performed with

primers flanking the insertions (*HSP22*; two primers in a). No *HSP22* expression was detected in the mutant seedlings, as reported previously[15]. *EIF4A1* was used for a loading control. Three independent experiments were performed and similar results were obtained. **e** and **f**, Wild-type (e) and *hsp22 hsp17.6c* (f) seedlings grown under control condition. **g** and **h**, Wild type (g) and *hsp22 hsp17.6c* (h) grown under +HS condition. **i**, Quantification of seedling fresh weights. Sample minimum (lower bar); lower quartile (box); median (middle line); upper quartile (box); sample maximum (upper bar). Asterisks indicate significant difference at 0.05 levels based on a two-tailed Student's *t*-test. * $p < 0.05$. NS, nonsignificant. **j**, Quantification of survival rate. Ten-day-old seedlings grown under three different temperature conditions were categorized into three groups based on phenotypic severity: green, normal growth; light green, partially damaged; white, perished. Significance was determined by χ^2 test and the post-hoc test that followed. NS, nonsignificant. $n > 78$. Although fresh weight in acclimaized *hsp22 hsp17.6c* double mutants was significantly lighter than that in acclimaized wild type after heat shock, no difference in survival rate was observed. The lack of difference in survival rate of *hsp22 hsp17.6c* double mutants, while there is a difference in *jmjq* mutants, suggests that other differentially expressed genes, such as *HSP21* may also contribute to phenotypic consequence of *jmjq* mutants for heat acclimation.

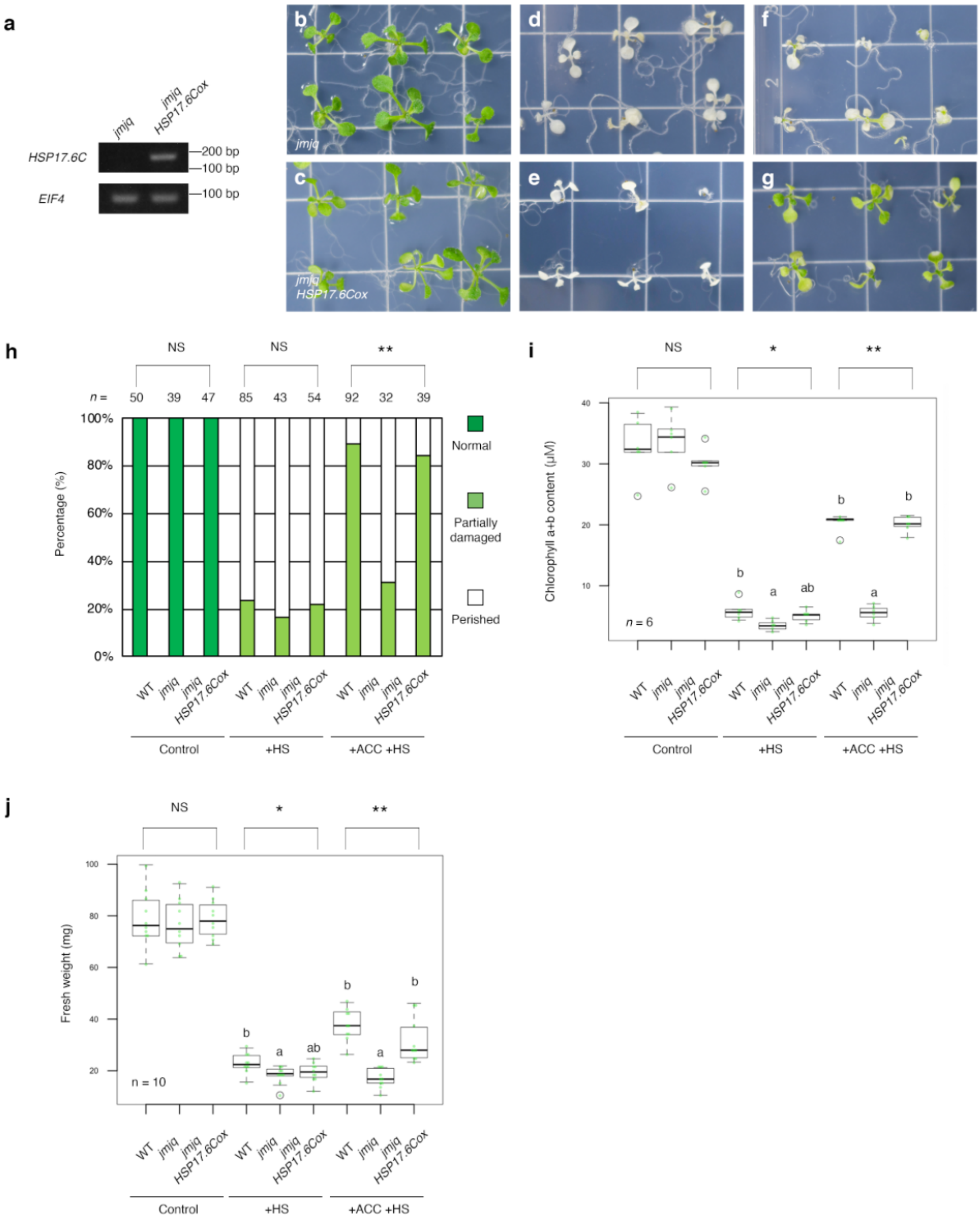

Supplementary Fig. 24 Ectopic expression of *HSP17.6C* rescued the heat acclimation phenotype in *jmjq* mutants. a, Expression of *HSP17.6C* (above) and *EIF4A1* (below) in *jmjq* and *jmjq 35S::HSP17.6C* seedlings. *EIF4A1* was used for a loading control. Three independent experiments were performed and similar results were obtained. **b** and **c**, *jmjq* (b) and *jmjq 35S::HSP17.6C* (c)

grown under control condition. **d** and **e**, Wild type (d) and *jmjq* (e) grown under +HS condition. **f** and **g**, Wild type (f) and *jmjq* (g) grown under +ACC +HS condition. **h**, Quantification of survival rate. Ten-day-old seedlings grown under three different temperature conditions were categorized into three groups based on phenotypic severity: green, normal growth; light green, partially damaged; white, perished. Significance was determined by χ^2 test and the post-hoc test that followed. $n > 31$. **i**, Quantification of chlorophyll contents. Sample minimum (lower bar); lower quartile (box); median (middle line); upper quartile (box); sample maximum (upper bar). Light green jitter dots and white circles represent the chlorophyll content from each sample and statistical outliers, respectively. Asterisks indicate significant differences based on one-way ANOVA test. $^*p < 1.0 \times 10^{-2}$, $^{**}p < 1.0 \times 10^{-3}$. Different letters indicate significant differences, while the same letters indicate non-significant differences based on post-hoc Tukey's HSD test. $p < 0.05$. NS, nonsignificant. **j**, Quantification of seedling fresh weights. Sample minimum (lower bar); lower quartile (box); median (middle line); upper quartile (box); sample maximum (upper bar). Asterisks indicate significant differences based on one-way ANOVA test. $^*p < 5.0 \times 10^{-2}$, $^{**}p < 1.0 \times 10^{-4}$. Different letters indicate significant differences, while the same letters indicate non-significant differences based on post-hoc Tukey's HSD test. $p < 0.05$. NS, nonsignificant.

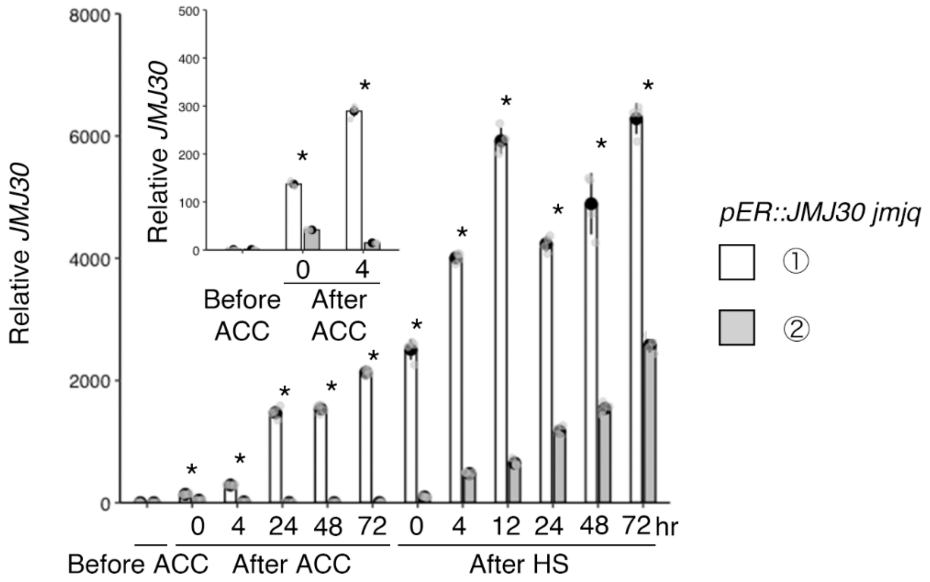

Supplementary Fig. 25 Application of estradiol immediately induces *JMJ30* expression. Gene expression levels of *JMJ30* in the *pER8::JMJ30* transgenic plants in the *jmjq* mutant background with β-estradiol application before acclimation and before heat shock. Asterisks indicate significant difference at 0.05 levels based on a two-tailed Student's *t*-test at the same time point.

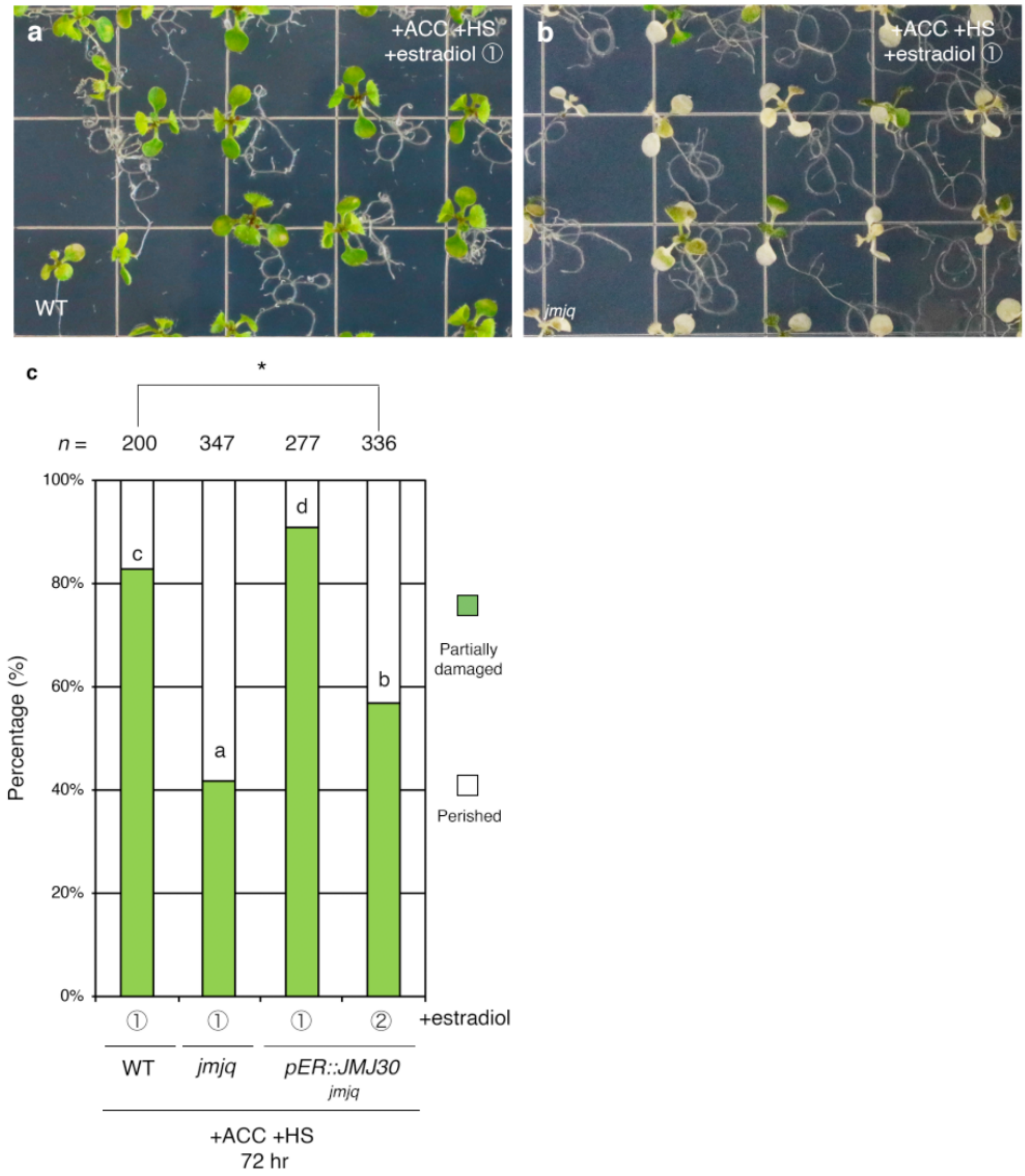

Supplementary Fig. 26 Induction of *JMJ30* in *jmjq* mutants prior to acclimation rescues the mutant phenotype. a and **b**, Images of wild type (a) and the *jmjq* mutant (b) with β-estradiol application before acclimation (left) and before heat shock (right). Plants were grown under +ACC +HS condition. Those two panels are both negative controls that show that the β-estradiol treatment itself did not trigger phenotypic rescue. **c**, Quantification of survival rate shown in Fig. 4b and Supplementary Fig. 26a and b. Ten-day-old seedlings grown

under +ACC +HS condition with β-estradiol application before acclimation and before heat shock were categorized into three groups based on phenotypic severity: green, normal growth; light green, partially damaged; white, perished. Significance was determined by χ^2 test. $n > 199$.

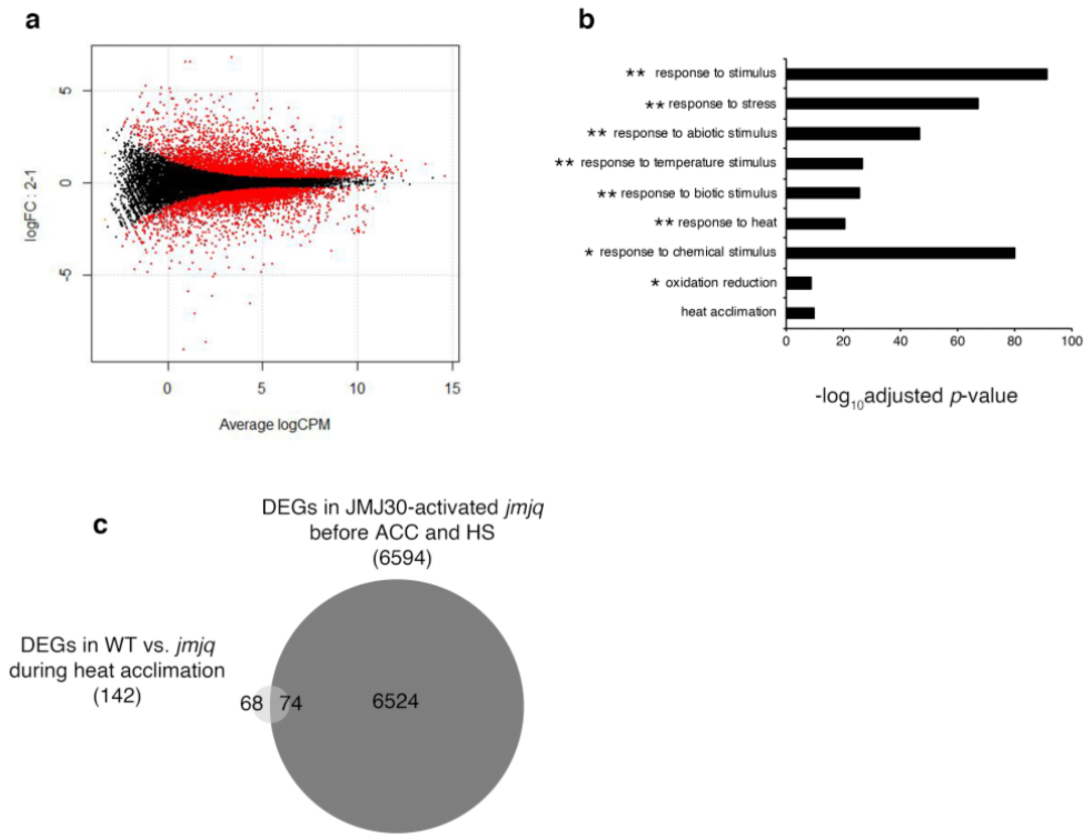

Supplementary Fig. 27 Induction of *JMJ30* in *jmjq* mutants prior to acclimation triggers heat-acclimation-related gene expression. a, The MA plots represent each gene with a dot. The *x* axis is average log CPM over all genes; the *y* axis is the $\log_2$ fold change of normalized count between *pER8::JMJ30* transgenic plants in the *jmjq* mutant background with β-estradiol application before acclimation and before heat shock. Genes with FDR <0.05 are shown in red. 6594 genes were differentially expressed when *JMJ30* was misexpressed before acclimation or before heat shock. **b**, Gene ontology (GO) term enrichment analysis of 6594 genes. Selected GO terms determined by their $-\log_{10}$-adjusted *p*-values based on two-tailed *z*-test are shown. All enriched GO terms are shown in Supplementary Data S8. **, GO terms seen in Fig. 2b; *, GO terms similar to those observed in Fig. 2b. Similar pathways were affected in *jmjq* mutants and JMJ30-induced plants prior to acclimation. **c**, Venn diagram showing the overlap between differentially expressed genes in wild type and *jmjq* mutants, and differentially expressed genes in *pER8::JMJ30*

transgenic plants in the *jmjq* mutant background with β-estradiol application before acclimation and before heat shock. This overlap was significantly larger than expected by chance ($p = 3.0 \times 10^{-18}$). Existence of non-overlapped genes may also suggest an indirect effect due to pleiotropic changes in gene expression by overexpression. The 74 overlapping genes included *HSP17.6C*, *HSP21*, and *HSP22* as well as *HSP23.6-MITO*, *HSP17.6 II*, *HSP70B*, and *HSP70-T2*.

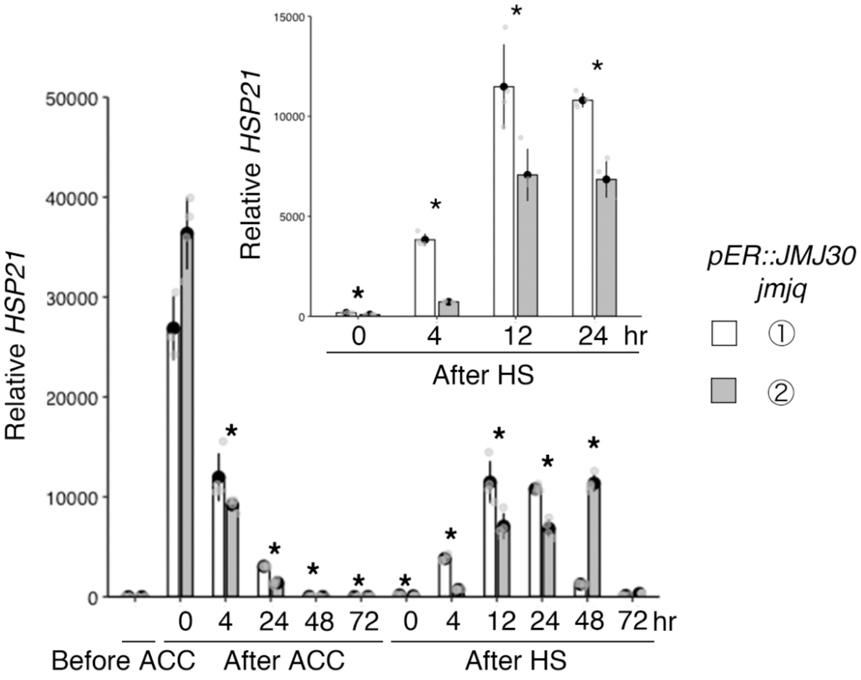

Supplementary Fig. 28 *HSP21* expression in *jmjq* mutants with and without induction of *JMJ30*. Gene expression levels of *HSP21* in the *pER8::JMJ30* transgenic plants in the *jmjq* mutant background before acclimation and heat shock. A two-tailed Student's *t*-test between *HSP21* expression in *pER8::JMJ30* transgenic plants in the *jmjq* mutant background subjected to β-estradiol application before acclimation and before heat shock, *$p < 0.05$.

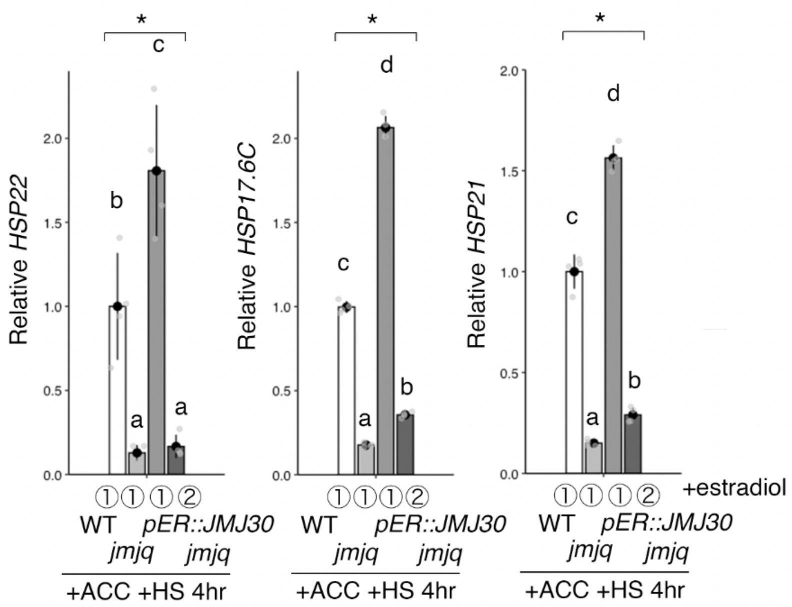

Supplementary Fig. 29 *HSP22*, *HSP17.6C*, and *HSP21* expression in *JMJ30*-induced *jmjq* mutants before ACC and HS treatment. Gene expression levels of *HSP22*, *HSP17.6C*, and *HSP21* in the *pER8::JMJ30* transgenic plants in the *jmjq* mutant background after β-estradiol application and before acclimation and heat shock. Asterisks indicate significant differences based on one-way ANOVA test. *$p < 0.05$. Different letters indicate significant differences, while the same letters indicate non-significant differences based on post-hoc Tukey's HSD test. $p < 0.05$.

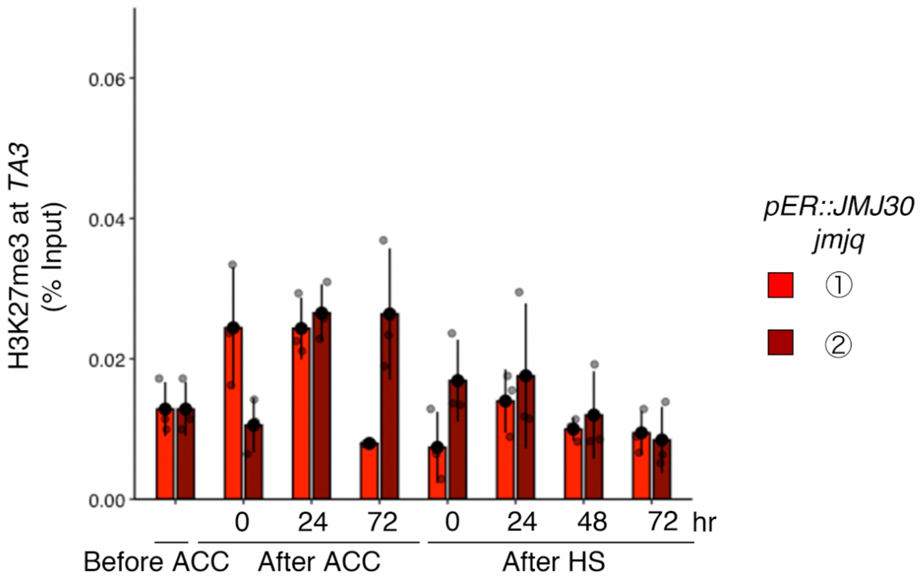

Supplementary Fig. 30 H3K27me3 levels at the *TA*3 locus in the wild type and *jmjq* mutants. H3K27me3 levels in the wild type and *jmjq* mutants at *TA3*, as determined by ChIP-qPCR. Grey jitter dots represent expression level from each sample. No difference at the *TA3* locus was observed by a two-tailed Student's *t*-test.

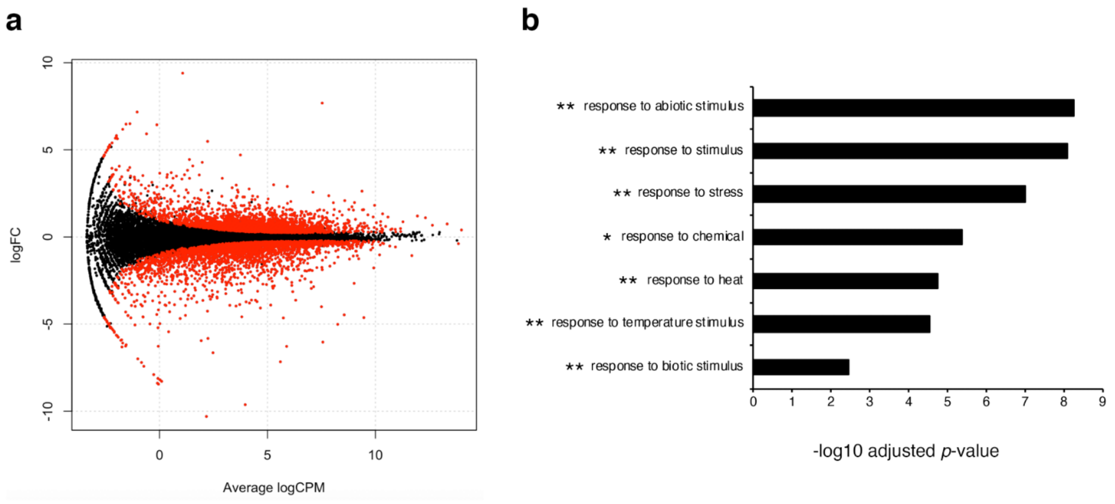

Supplementary Fig. 31 Gene expression in wild type and *jmjq* mutants under the Nara condition. a, The MA plots represent each gene with a dot. The *x* axis is average log CPM over all genes; the *y* axis is the $\log_2$ fold change of normalized count between wild type and *jmjq* mutants under the Nara condition. Genes with FDR <0.05 are shown in red. 5947 genes were differentially expressed. **b**, Gene ontology (GO) term enrichment analysis of 46 genes. Selected GO terms determined by their $-\log_{10}$-adjusted *p*-values based on two-tailed *z*-test are shown. All enriched GO terms are shown in Supplementary Data 12. **, GO terms seen in Fig. 2b; *, GO terms similar to those observed in Fig. 2b. Similar pathways were affected in *jmjq* mutants and JMJ30-induced plants prior to acclimation.

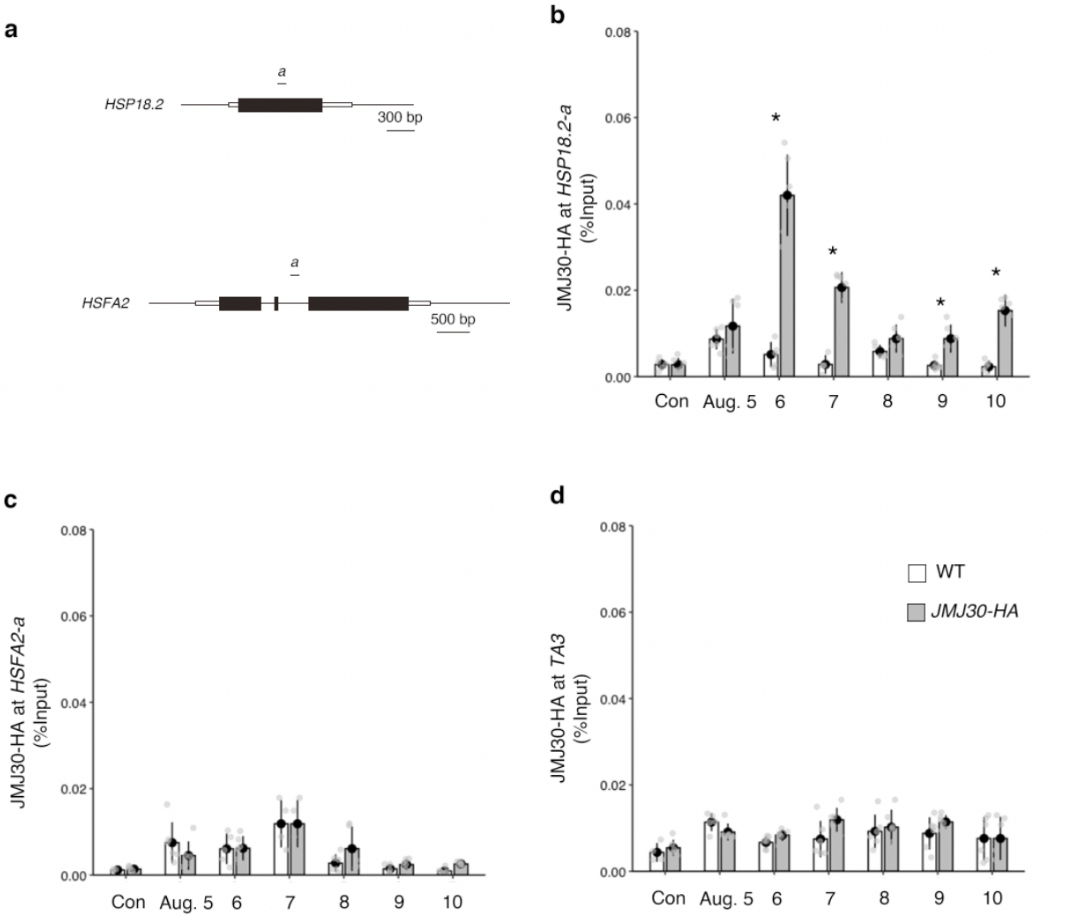

Supplementary Fig. 32 JMJ30 binding at the *HSP18.2*, *HSFA2*, and *TA3* loci under the Nara condition a, Diagram of *HSP18.2*, *HSFA2*, and *TA3* loci and primers used. **b-d**, JMJ30-HA levels at *HSP18.2* (b), *HSFA2* (c), and *TA3* (d) in the wild type and *jmjq* mutants under the Nara condition, as determined by ChIP-qPCR. Gray jitter dots represent the expression level from each sample. A two-tailed Student's *t*-test between wild type and *jmjq* mutants at the same time point, *$p < 0.05$.

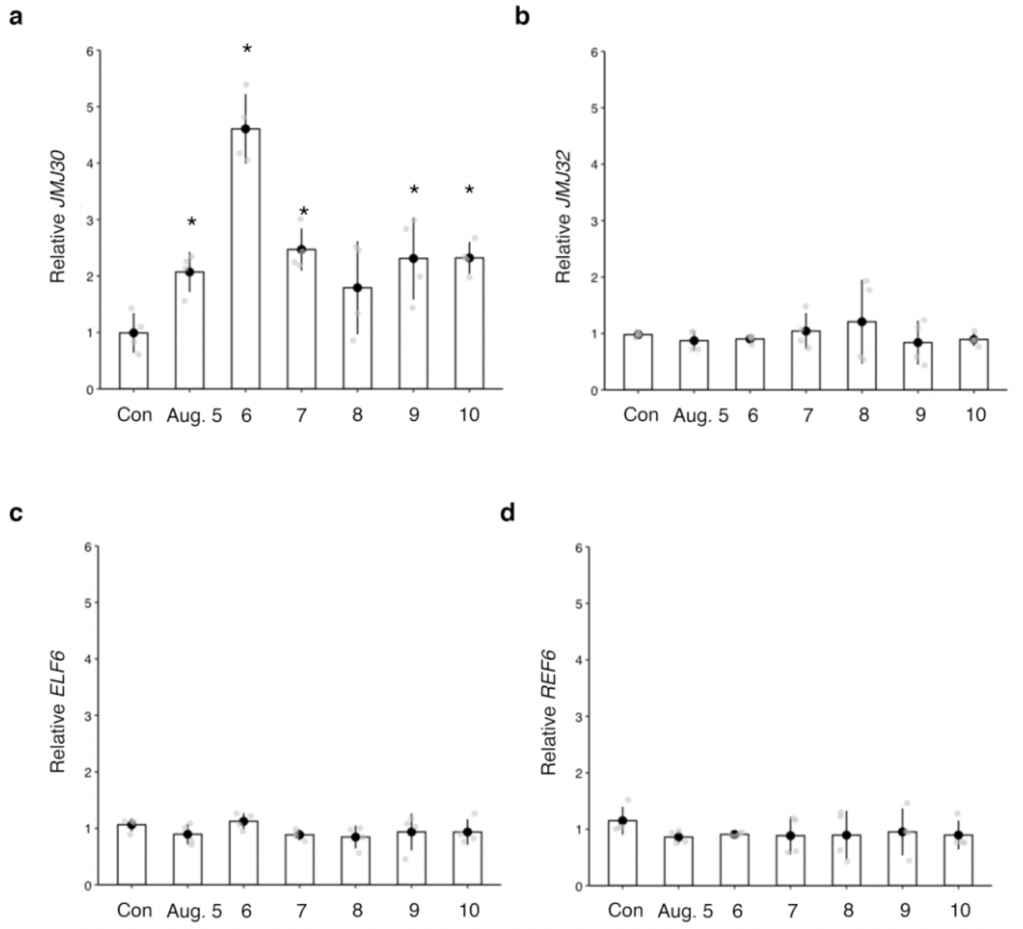

Supplementary Fig. 33 Expression of four *JMJ* genes in the wild type under the Nara condition a-d, qRT-PCR verification of the *JMJ30* (a), *JMJ32* (b), *ELF6* (c) and *REF6* (d) transcript levels in the wild type grown under the Nara conditions. Gray jitter dots represent expression level from each sample. A two-taild Student's *t*-test compared to the wild type before ACC, *$p < 0.05$.

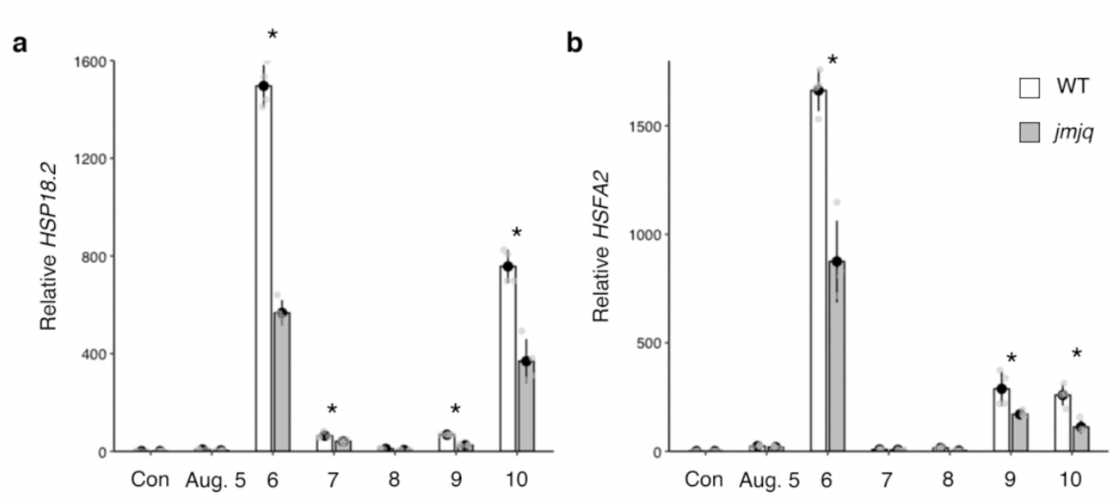

Supplementary Fig. 34 *HSP18.2* and *HSFA2* expression in the wild type and *jmjq* mutants under the Nara condition a, **b**, qRT-PCR verification of *HSP18.2* (a) and *HSFA2* (b) transcript levels in the wild type and *jmjq* mutants grown under Nara conditions. Gray jitter dots represent the expression level from each sample. Asterisks indicate significant difference at 0.05 levels based on a two-tailed Student's *t*-test between the wild type and *jmjq* mutants at the same time point.

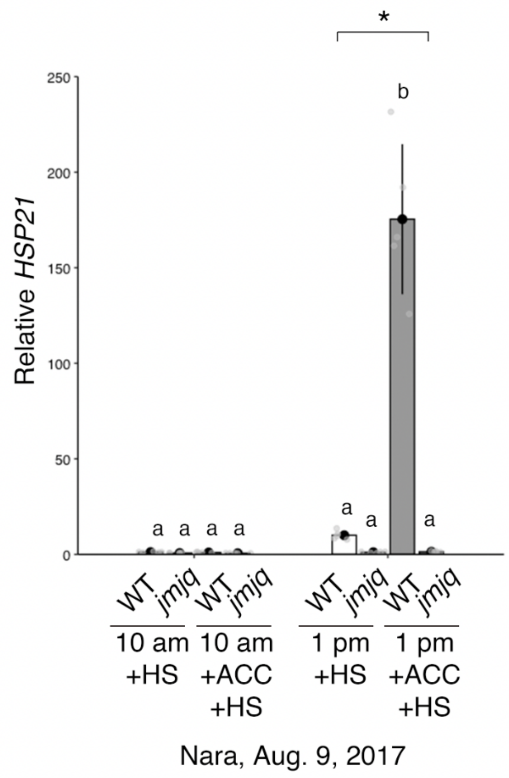

Supplementary Fig. 35 *HSP21* expression in wild type and *jmjq* mutants under the Nara condition. Gene expression levels of *HSP21* in the wild-type and *jmjq* mutants under the Nara condition. Asterisk indicates significant differences based on one-way ANOVA test. Different letters indicate significant differences, while the same letters indicate non-significant differences based on post-hoc Tukey’s HSD test.

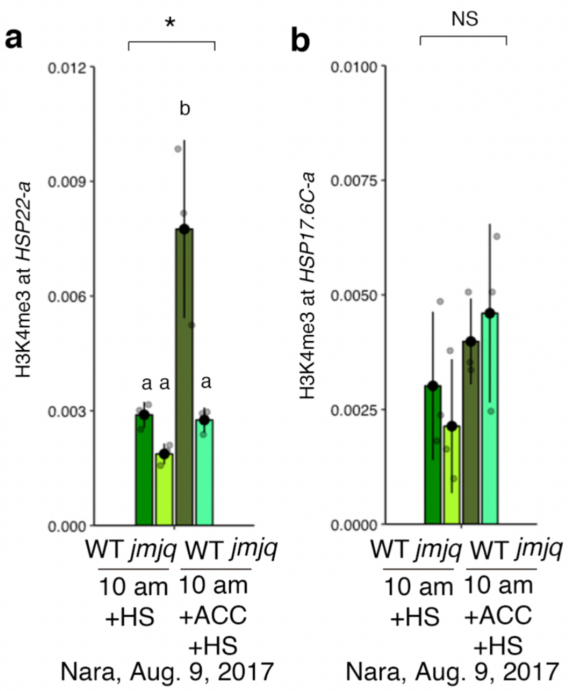

Supplementary Fig. 36 Histone modifications at the *HSP22*, and *HSP17.6C* loci under the Nara condition. a and **b**, H3K4me3 levels at *HSP22* (a), and *HSP17.6C* (b) in the wild type and *jmjq* mutants under the Nara condition. Asterisk indicates significant differences based on one-way ANOVA test. Different letters indicate significant differences, while the same letters indicate non-significant differences based on post-hoc Tukey's HSD test. $p < 0.05$. NS, nonsignificant. The difference in H3K4me3 enrichment between *HSP22* and *HSP17.6C* in wild type at 10 am under the Nara condition suggests that conditions are important for the regulation of *HSP22* and *HSP17.6C* by histone demethylases.

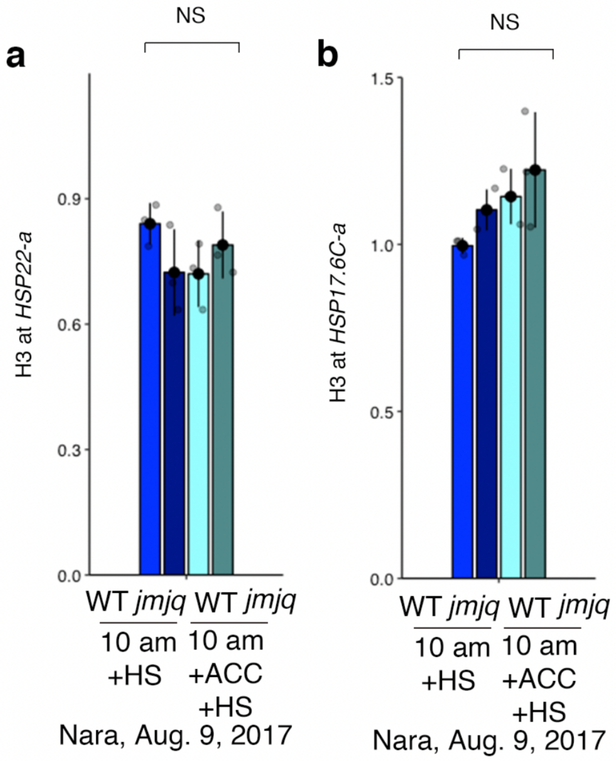

Supplementary Fig. 37 Histone H3 levels at the *HSP22*, and *HSP17.6C* loci under the Nara condition. a and **b**, H3 levels at *HSP22* (a), and *HSP17.6C* (b) in the wild type and *jmjq* mutants under the Nara condition. NS, nonsignificant based on one-way ANOVA test.

Supplementary Note 1. Mathematical model for histone modification.

Definition of transition rates between three states of histone modification

We developed a mathematical model describing state transitions of histone modification at the cell-population level to predict the level of *HSP* expression under changing temperature.

We focus on a locus with N units of nucleosomes. Each nucleosome is in one of the following three states, actively modified (A), unmodified (U), and repressively modified (R) (Fig. 3g), as assumed in a previous study[25,26]. In an actively transcribed *HSP* chromatin, H3K4me3 modifications are enriched. The unmodified state changes to the repressively modified state by the addition of repressive histone marks. H3K27me3 marks are associated with the repressed transcriptional state of genes. Let i and j be the numbers of actively- and repressively-modified nucleosomes, respectively ($0 \leq i, j \leq N$ and $i + j \leq N$). The number of unmodified nucleosomes is calculated as $N - i - j$, and thus every possible state of the locus can be described with a pair of integers (i, j). Transcription of the locus occurs only when all N nucleosomes are in state A (i.e., when $(i, j) = (N, 0)$).

State transition of each nucleosome is modeled based on a previous study[26]. Consider a sufficiently short period of time during which at most one of the N units in each locus can change its state. Let a transition rate at which a nucleosome in the state X changes to the state Y as $r_{X \to Y}$, where $(X, Y) \in \{(\mathrm{U}, \mathrm{A}), (\mathrm{R}, \mathrm{U}), (\mathrm{U}, \mathrm{R}), (\mathrm{A}, \mathrm{U})\}$. Due to positive feedback mechanisms involved in the recruitment of histone modifying complexes, all-A and all-R modification states can be stable at the same time; that is, the system can be 'bistable' [25,26]. This positive feedback is incorporated in the model by assuming transition rates as a function of the number of nucleosomes in the state A or R. To be specific, transition rate from the state A or U to the state A increases when the number of A within the focal locus (i) is greater. It follows that $r_{\mathrm{U} \to \mathrm{A}}$ and $r_{\mathrm{R} \to \mathrm{U}}$ are increasing functions of i as follows:

$$r_{\mathrm{U} \to \mathrm{A}}(i; T) = v_{\mathrm{U} \to \mathrm{A}} \cdot P(T) \cdot (1 + \beta_{\mathrm{U} \to \mathrm{A}} \cdot i) \qquad \text{(Eq. 1a)}$$

$$r_{\mathrm{R} \to \mathrm{U}}(i; T) = v_{\mathrm{R} \to \mathrm{U}} \cdot P(T) \cdot (1 + \beta_{\mathrm{R} \to \mathrm{U}} \cdot i), \qquad \text{(Eq. 1b)}$$

where $v_{X \to Y}$ is a transition rate when the number of A equals zero under ambient temperature of 22˚C. The function $P(T)$ represents the dependence of transition rates on temperature (T). High temperature is expected to induce expression of *HSP* genes, and we define $P(T)$ as follows:

$$\begin{cases} P(22) = 1 & \text{under ambient temperature of } 22^\circ\text{C} \\ P(37) = P_{\text{ACC}} & \text{during acclimation under } 37^\circ\text{C} \\ P(44) = P_{\text{HS}} & \text{during heat shock under } 44^\circ\text{C} \end{cases}.$$

Coefficients $\beta_{X\to Y}$ represent the strength of positive feedback. This linear formulation is not a strong assumption, as we obtain similar results as long as the feedback term is a monotonically increasing function of i.

Similarly, $r_{\text{U}\to\text{R}}$ and $r_{\text{A}\to\text{U}}$ are given as increasing functions of the number of R (j) as follows:

$$r_{\text{U}\to\text{R}}(j) = v_{\text{U}\to\text{R}} \cdot (1 + \beta_{\text{U}\to\text{R}} \cdot j) \qquad \text{(Eq. 2a)}$$

$$r_{\text{A}\to\text{U}}(j) = v_{\text{A}\to\text{U}} \cdot (1 + \beta_{\text{A}\to\text{U}} \cdot j), \qquad \text{(Eq. 2b)}$$

where $v_{X\to Y}$ and $\beta_{X\to Y}$ are defined similarly as in Eq. (1).

Dynamics of histone modification at the cell-population level

Using the transition rate functions defined in Eqs. (1) and (2), we describe the dynamics describing state change of histone modification at the cell-population level by assuming no interaction between cells. Let $s_{i,j}(t)$ be the proportion of cells in the state (i,j) ($\sum_{i=0}^{N}\sum_{j=0}^{N-i} s_{i,j}(t) = 1$). Some fraction of cells in the state (i,j) will transition to the state $(i+1,j)$ by changing the state of one nucleosome from U to A at a rate $r_{\text{U}\to\text{A}}(i;T)$. At the same time, some of cells in the state $(i+1,j)$ will shift into the state (i,j) by reducing the number of A by one at a rate $r_{\text{A}\to\text{U}}(i+1)$. The above outflow and inflow between the states (i,j) and $(i+1,j)$ is summarized in a mathematical form as I^+:

$$I^+ = -r_{\text{U}\to\text{A}}(i;T) \cdot s_{i,j}(t) + r_{\text{A}\to\text{U}}(i+1) \cdot s_{i+1,j}(t). \qquad \text{(Eq. 3)}$$

Likewise, the subpopulation of cells in the state (i,j) can have outflow toward and inflow from another neighboring state $(i,j+1)$, which is denoted by J^+:

$$J^+ = -r_{\text{U}\to\text{R}}(j) \cdot s_{i,j}(t) + r_{\text{R}\to\text{U}}(j+1;T) \cdot s_{i,j+1}(t). \qquad \text{(Eq. 4)}$$

Note that Eqs. (3) and (4) presume that the state (i,j) involves at least one unmodified unit (i.e., $N-i-j > 0$ or $i+j < N$). Similarly, if cells are in states with at least one nucleosome in state A (i.e., $i \geq 1$) there are flows between the state (i,j) and its neighboring state $(i-1,j)$:

$$I^- = -r_{\text{A}\to\text{U}}(i;T) \cdot s_{i,j}(t) + r_{\text{U}\to\text{A}}(i-1;T) \cdot s_{i-1,j}(t) \qquad \text{(Eq. 5)}$$

In the end, flows exist between the state with at least one nucleosome in state R ($j \geq 1$) and its neighboring state $(i,j-1)$:

$$J^- = -r_{\text{R}\to\text{U}}(j;T) \cdot s_{i,j}(t) + r_{\text{U}\to\text{R}}(j-1) \cdot s_{i,j-1}(t) \qquad \text{(Eq. 6)}$$

Using Kronecker's $\delta_{x,y}$ ($\delta_{x,y} = 1$ if $x = y$ and $\delta_{x,y} = 0$ otherwise), we have

$$\frac{\mathrm{d}}{\mathrm{d}t}s_{i,j} = \left(1-\delta_{i+j,N}\right)(I^+ + J^+) + \left(1-\delta_{i,0}\right)I^- + \left(1-\delta_{j,0}\right)J^-. \qquad \text{(Eq. 7)}$$

It can be mathematically shown that the above system has a unique and globally stable equilibrium under a constant temperature. We define the equilibrium state at $T = 22$ (°C) as $\left(\hat{s}_{0,0}, \hat{s}_{0,1}, \dots, \hat{s}_{N,0}\right)$.

Because we assume that *HSP* genes are expressed only when all the N nucleosomes are in state A, expression levels of *HSP* genes at time t, denoted as $m(t)$, are in proportion to the fraction of cells in the state (*N*, 0), $s_{N,0}(t)$, as follows:

$$m(t) = q \cdot s_{N,0}(t), \qquad \text{(Eq. 8)}$$

where q is a positive constant. In the following analyses, we substitute $q = 1/\hat{s}_{N,0}$ so that $m(0) = 1$. Although we do not observe cell death, a majority of cells are damaged after heat shock treatment (Supplementary Fig. 9). Thus, we assume that the fraction h of cells stop transcription of *HSP* genes right after the heat shock at $t = t_{\mathrm{HS}}$ and gradually recover their transcription activity at a constant rate r:

$$m(t) = \begin{cases} q \cdot s_{N,0}(t) & \text{for } t \le t_{\mathrm{HS}} \\ q \cdot \left(1 - h \cdot e^{-r\cdot(t-t_{\mathrm{HS}})}\right) \cdot s_{N,0}(t) & \text{for } t > t_{\mathrm{HS}} \end{cases}. \qquad \text{(Eq. 9)}$$

Because the ORF length of *HSP22* is 588 bp, we choose $N = 3$ for the following analyses. In our formalization, we assume that the order of histone modification at different nucleosomes is not random, but there is a specific order (e.g. a nucleosome closest to the transcription start site is the first to be repressively modified.). For parameter fitting using wild type, the sum of squared errors between log-transformed experimental and simulation data was considered as a cost function to be minimized. The present model has as many as 12 free parameters, in which case it is generally difficult to specify the single optimal solution. Therefore, we used 144 different sets of initial parameter values that were randomly chosen from fixed ranges for each parameter (Supplementary Table 1) and obtained the best fit set of parameters (Supplementary Table 2). For parameter fitting, time unit is set to an hour. We also performed parameter fitting using experimental data from *jmjq* mutant. We chose the wild-type best-fit parameters as initial values for this analysis on the basis that a mutant would not be drastically different from wild type. Comparison of best fit parameters between wild type and *jmjq* mutant showed that the *jmjq* mutant has notably greater values for the strength of positive feedback for the transition from A to U ($\beta_{\mathrm{A}\to\mathrm{U}}$) and temperature dependence during heat shock (P_{HS}). On the contrary, the *jmjq* mutant has smaller values for recovery rate of transcription activity after heat damage (r) and the basic transition rate from R to U ($v_{\mathrm{R}\to\mathrm{U}}$). The greater positive feedback effect

($\beta_{\mathrm{A}\to\mathrm{U}}$) and the smaller basic transition rate from R to U corresponds to the feature that the *jmjq* mutant lacks the demethylation activity for the repressive mark H3K27me3.

The model can predict dynamics of histone modification states that have not measured in the experiments (Supplementary Fig. 22). The results also revealed the importance to the memory effect of having multiple modifiable sites.

Predicting *HSP* expression profiles in natural temperature conditions

We also performed simulations with temperature profiles obtained from several fields. Based on the suggestion from the above parameter fittings that $P(T)$ is a nonlinear, monotonically increasing function of temperature, we assume that $P(T)$ takes the form of exponential function:

$$P(T) = k_1 + k_2 \cdot e^{k_3 \cdot T}, \qquad \text{(Eq. 10)}$$

where k_1, k_2, and k_3 are constant parameters. Substituting the definition $P(22) = 1$ and the best-fit parameter values, $P(37) = P_{\mathrm{ACC}}$ and $P(44) = P_{\mathrm{HS}}$, we obtained estimated values for those coefficients ($k_1 = 3.61 \times 10^{-6}$, $k_2 = 0.355$, and $k_3 = 0.911$). We interpolated hourly temperature data at Nara and substituted it into the present model to obtain Fig. 5g.

We used *Mathematica* 11.3.0.0 (Wolfram Research Inc.) for numerical simulations of the ordinary differential equations (Adams' method), parameter fitting (the interior point method), and interpolation (to sixth order polynomial). The source code can be found below (pp. 54-60).

```
In[ ]:= (* Definition *)
Clear["Global`*"]

n = 3;
tBegin = -1;

(* time points corresponding to experiment *)
tList = {0, 1, 5, 25, 49, 73, 220/3, 232/3, 256/3, 292/3, 364/3, 436/3};

(* time for acclimation *)
t0 = 0;
t1 = 1;

(* time for heat shock *)
t2 = 73;
t3 = 220 / 3;

myRamp[x_] := Max[0, x];
SetAttributes[myRamp, Listable];

model1StateSummary[tList_, α_?NumberQ, βru_?NumberQ, βur_?NumberQ, βau_?NumberQ,
    βua_?NumberQ, vua0_?NumberQ, vau0_?NumberQ, vur0_?NumberQ, vru0_?NumberQ,
    p_?NumberQ, q_?NumberQ, h_?NumberQ, r_?NumberQ, modeJ_ : False] := Module[
    {t, s, fru, fur, fau, fua, dsdt, acc, hs, vru, vua,
     vau, vur, sUnit, equilibrium, tEnd, sol},

    tEnd = Max[tList];

    fru[x_] := α + βru * x;
    fur[x_] := α + βur * x;
    fau[x_] := α + βau * x;
    fua[x_] := α + βua * x;
    dsdt[i_, j_][t_] := With[{k = n - i - j},
      Boole[i ≠ 0]
        * (vua[t] * fua[i - 1] * s[i - 1, j][t] - vau[t] * fau[j] * s[i, j][t])
       + Boole[k ≠ 0]
        * (vau[t] * fau[j] * s[i + 1, j][t] - vua[t] * fua[i] * s[i, j][t]
          + vru[t] * fru[i] * s[i, j + 1][t] - vur[t] * fur[j] * s[i, j][t])
       + Boole[j ≠ 0]
        * (vur[t] * fur[j - 1] * s[i, j - 1][t] - vru[t]
           * fru[i] * s[i, j][t])
     ];
    vru[t_] := vru0 * (1 + acc[t] * (p - 1) + hs[t] * (q - 1));
    vua[t_] := vua0 * (1 + acc[t] * (p - 1) + hs[t] * (q - 1));
    vau[t_] = vau0;
    vur[t_] = vur0;

    (* Obtain the equilibrium *)
    equilibrium =
     First[NSolve[
       Append[
        Flatten[Table[dsdt[i, j][tBegin] == 0 /.
```

```
      {acc[tBegin] → 0, hs[tBegin] → 0}, {i, 0, n - 1}, {j, 0, n - i}]],
    s[n, 0][tBegin] == 1 - Sum[s[i, j][tBegin], {i, 0, n - 1}, {j, 0, n - i}]
   ],
   Flatten[Table[s[i, j][tBegin], {i, 0, n}, {j, 0, n - i}]]
  ]];

(* Change unit *)
sUnit = Simplify[1 / (s[n, 0][tBegin] /. equilibrium)];

(* Solve ODE*)
sol = First[NDSolve[
   {
    Table[s[i, j]'[t] == dsdt[i, j][t], {i, 0, n}, {j, 0, n - i}],
    Table[
     s[i, j][tBegin] == (s[i, j][tBegin] /. equilibrium), {i, 0, n}, {j, 0, n - i}],
    (* acclimation: t0 < t < t1 *)
    acc[tBegin] == 0,
    WhenEvent[t > t0, acc[t] → 1],
    WhenEvent[t > t1, acc[t] → 0],
    (* heat shock: t2 < t < t3 *)
    hs[tBegin] == 0,
    WhenEvent[t > t2, hs[t] → 1],
    WhenEvent[t > t3, hs[t] → 0]
   },
   Join[Flatten[Table[s[i, j], {i, 0, n}, {j, 0, n - i}]], {acc, hs}],
   {t, tBegin, tEnd},
   DiscreteVariables → {acc, hs},
   Method → "StiffnessSwitching"
  ]];

(* Convert NDSolve solution to time series profile *)
If[modeJ,
 Return[Table[
    {t, Sum[s[i, #][t], {i, 0, n - #}] & /@ Range[0, n]},
    {t, tList}]] /. sol,
 Return[Table[
    {t, Sum[s[#, j][t], {j, 0, n - #}] & /@ Range[0, n]},
    {t, tList}]] /. sol]
];
```

```
(* Exec *)

tProfile = Range[0, Max[tList], 1 / 60];

(* WT *)
parsWT = {
   βru → 0.49607155677403914`,
   βur → 4.666653257362136`,
   βau → 8.20858290077248`,
   βua → 1.2617865570935758`,
   vru0 → 0.06322515753151707`,
   vur0 → 4.849433876637601`,
   vau0 → 0.21465092625686888`,
   vua0 → 1.6220079738367554`,
   p → 19.13449631247978`,
   q → 219.44448919226684`,
   h → 1`,
   r → 0.009995742370280614`
  };
wtProfiles = model1StateSummary[tProfile, 1,
    βru, βur, βau, βua, vua0, vau0, vur0, vru0, p, q, h, r] /. parsWT;
Map[(wtProfile[#] = Transpose[Last@Transpose[wtProfiles]][[# + 1]]) &, Range[0, 3]];
wtSummary = model1StateSummary[tList, 1, βru,
    βur, βau, βua, vua0, vau0, vur0, vru0, p, q, h, r] /. parsWT;
wtSummaryj = model1StateSummary[tList, 1, βru, βur, βau, βua,
    vua0, vau0, vur0, vru0, p, q, h, r, True] /. parsWT;

(* mutant *)
parsMT = {
   βru → 0.5429974311474887`,
   βur → 4.551525456899735`,
   βau → 10.425736537992044`,
   βua → 1.2673072133225527`,
   vru0 → 0.050555331634477584`,
   vur0 → 4.867242333492629`,
   vau0 → 0.2027936404008631`,
   vua0 → 1.6156268725026892`,
   p → 19.13234484190158`,
   q → 263.4024048787131`,
   h → 1`,
   r → 0.007789601398272258`
  };
mtProfiles = model1StateSummary[tProfile, 1,
    βru, βur, βau, βua, vua0, vau0, vur0, vru0, p, q, h, r] /. parsMT;
Map[(mtProfile[#] = Transpose[Last@Transpose[mtProfiles]][[# + 1]]) &, Range[0, 3]];
mtSummary = model1StateSummary[tList, 1, βru,
    βur, βau, βua, vua0, vau0, vur0, vru0, p, q, h, r] /. parsMT;
mtSummaryj = model1StateSummary[tList, 1, βru, βur, βau, βua,
    vua0, vau0, vur0, vru0, p, q, h, r, True] /. parsMT;
```

```
(* Output *)
ListPlot[Evaluate[{
   Transpose[{tProfile, wtProfile[2]}],
   Transpose[{tProfile, mtProfile[2]}]
  }], Joined → True, PlotStyle → {Black, Red},
 PlotRange → All]
```

Out[ ]=

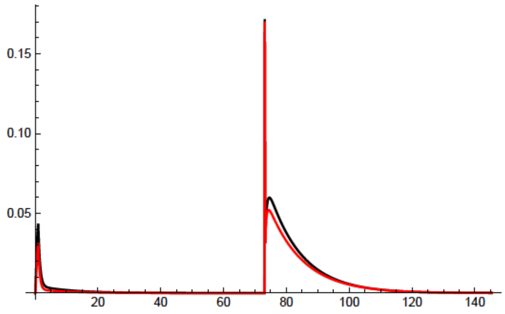

In[ ]:= (* Figs *)

```
(* Color setting *)
{wtRed, mtRed, wtGreen, mtGreen} = RGBColor[# / 255] & /@ {
    {255, 0, 0}, {163, 0, 0},
    {0, 152, 50}, {153, 255, 51}};
csA = {wtGreen, mtGreen};
csR = {wtRed, mtRed};

(* WT *)
BarChart[
 Transpose@Map[Part[Transpose@Last@Transpose[#], 1] &, {wtSummary, mtSummary}],
 BaseStyle → {"Arial", Bold, 12}, ChartStyle → csA,
 Axes → {None, Automatic}, PlotRange → {0, 0.95}]
BarChart[Transpose@Map[Part[Transpose@Last@Transpose[#], 2] &,
   {wtSummary, mtSummary}], BaseStyle → {"Arial", Bold, 12},
 ChartStyle → csA, Axes → {None, Automatic}, PlotRange → {0, 0.125}]
BarChart[Transpose@Map[Part[Transpose@Last@Transpose[#], 3] &,
   {wtSummary, mtSummary}], BaseStyle → {"Arial", Bold, 12},
 ChartStyle → csA, Axes → {None, Automatic}, PlotRange → {0, 0.125}]
BarChart[Transpose@Map[Part[Transpose@Last@Transpose[#], 4] &,
   {wtSummary, mtSummary}], BaseStyle → {"Arial", Bold, 12},
 ChartStyle → csA, Axes → {None, Automatic}, PlotRange → {0, 0.95}]

(* mutant *)
BarChart[
 Transpose@Map[Part[Transpose@Last@Transpose[#], 4] &, {wtSummaryj, mtSummaryj}],
 BaseStyle → {"Arial", Bold, 12}, ChartStyle → csR,
 Axes → {None, Automatic}, PlotRange → {0, 0.95}]
BarChart[Transpose@Map[Part[Transpose@Last@Transpose[#], 3] &,
   {wtSummaryj, mtSummaryj}], BaseStyle → {"Arial", Bold, 12},
 ChartStyle → csR, Axes → {None, Automatic}, PlotRange → {0, 0.125}]
BarChart[Transpose@Map[Part[Transpose@Last@Transpose[#], 2] &,
   {wtSummaryj, mtSummaryj}], BaseStyle → {"Arial", Bold, 12},
 ChartStyle → csR, Axes → {None, Automatic}, PlotRange → {0, 0.125}]
BarChart[Transpose@Map[Part[Transpose@Last@Transpose[#], 1] &,
   {wtSummaryj, mtSummaryj}], BaseStyle → {"Arial", Bold, 12},
 ChartStyle → csR, Axes → {None, Automatic}, PlotRange → {0, 0.95}]
```

Out[ ]=

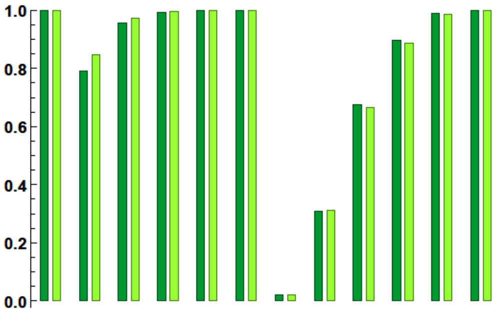

Out[∘]=

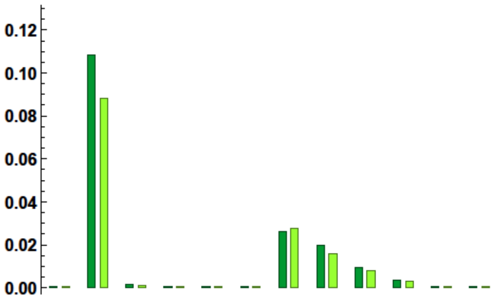

Out[∘]=

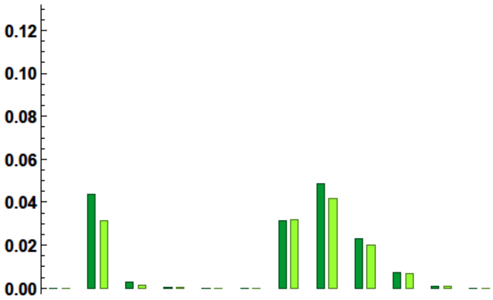

Out[∘]=

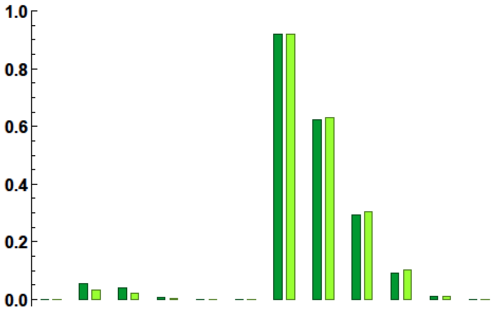

Out[∘]=

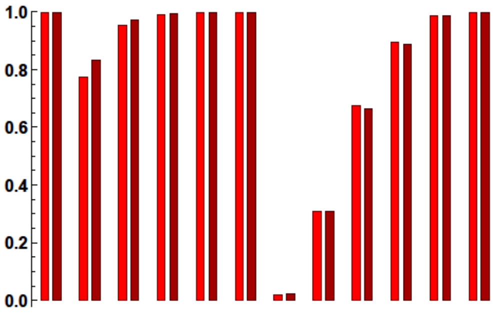

Out[ ]=

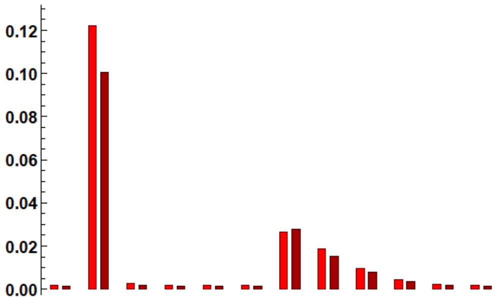
0.12
0.10
0.08
0.06
0.04
0.02
0.00

Out[ ]=

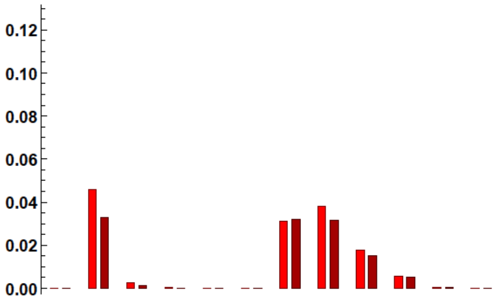
0.12
0.10
0.08
0.06
0.04
0.02
0.00

Out[ ]=

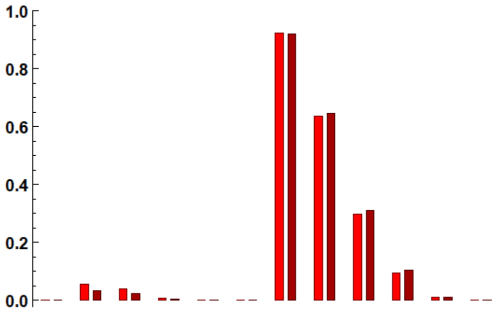
1.0
0.8
0.6
0.4
0.2
0.0

Supplementary Table 1. Ranges of random values from which initial parameter values were chosen.

	**Lower limit**	**Upper limit**
$v_{X\to Y}$	0.1	2
$\beta_{\mathrm{X\to Y}}$	0.1	7
$P_{\mathrm{ACC}}, P_{\mathrm{HS}}$	1	100
h, r	0.1	0.9

Supplementary Table 2. Best-fit parameter values.

	WT parameters	*jmjq* parameters	(*jmjq* − WT) / WT
$v_{\mathrm{U \to A}}$	1.6220	1.6156	$-$
$v_{\mathrm{R \to U}}$	0.063225	0.050555	-20%
$v_{\mathrm{U \to R}}$	4.8494	4.8672	$-$
$v_{\mathrm{A \to U}}$	0.21465	0.20279	-5%
$\beta_{\mathrm{U \to A}}$	1.2618	1.2673	$+9\%$
$\beta_{\mathrm{R \to U}}$	0.49607	0.54300	$-$
$\beta_{\mathrm{U \to R}}$	4.6667	4.6891	$-$
$\beta_{\mathrm{A \to U}}$	8.2086	10.426	$+27\%$
P_{ACC}	19.134	19.132	$-$
P_{HS}	219.44	263.40	$+20\%$
h	1	1	$-$
r	0.0099957	0.0077896	-22%
Value of cost function	10.70	21.74	$-$

Note: Values within $\pm 1\%$ are not shown in the far-right column.
