# Supplementary material for: H3K27me3 demethylases alter HSP22 and HSP17.6C expression in response to recurring heat in Arabidopsis
Source: Nat Commun. 2021 Jun 9;12:3480. doi: 10.1038/s41467-021-23766-w (PMC8190089; doi:10.1038/s41467-021-23766-w)
Supplement: Supplementary file 2 — Descriptions of Additional Supplementary Files [file 41467_2021_23766_MOESM2_ESM.pdf]

Descriptions of Additional Supplementary Files

Supplementary Data 1
Description: Summary of RNA-seq results in wild type and jmjq mutants with and without acclimation.

Supplementary Data 2
Description: 142 genes differentially expressed between wild type and jmjq mutants during heat acclimation.

Supplementary Data 3
Description: Go terms for 142 genes differentially expressed between wild type and jmjq mutants during heat acclimation.

Supplementary Data 4
Description: H3K27 trimethylated genes in wild type and jmjq mutants with and without acclimation.

Supplementary Data 5
Description: Histone H3 enriched genes in wild type and jmjq mutants with and without acclimation.

Supplementary Data 6
Description: H3K4 trimethylated genes in wild type and jmjq mutants with and without acclimation.

Supplementary Data 7
Description: Summary of RNA-seq results in JMJ30-induced jmjq mutants prior to acclimation or heat shock.

Supplementary Data 8
Description: GO terms for 6594 genes differentially expressed between JMJ30-induced jmjq mutants prior to acclimation and prior to heat shock.

Supplementary Data 9
Description: Measurement of temperature in growth chamber mimicking fluctuating field temperature conditions.

Supplementary Data 10
Description: Summary of RNA-seq results in wild type and jmjq mutants under the Nara condition.

Supplementary Data 11
Description: Common downstream genes between RNA-seq experiments.

Supplementary Data 12
Description: Go terms for the 46 genes differentially expressed between the three RNA-seq datasets.

Supplementary Data 13
Description: jmj mutants used in this study.

Supplementary Data 14
Description: Primers used in this study.
