# Supplementary material for: H3K27me3 demethylases alter HSP22 and HSP17.6C expression in response to recurring heat in Arabidopsis
Source: Nat Commun. 2021 Jun 9;12:3480. doi: 10.1038/s41467-021-23766-w (PMC8190089; doi:10.1038/s41467-021-23766-w)
Supplement: Supplementary file 17 — Reporting Summary [file 41467_2021_23766_MOESM17_ESM.pdf]

nature research

Corresponding author(s): Toshiro Ito/Nobutoshi Yamaguchi

Last updated by author(s): Apr 29, 2021

Reporting Summary

Nature Research wishes to improve the reproducibility of the work that we publish. This form provides structure for consistency and transparency in reporting. For further information on Nature Research policies, see Authors & Referees and the Editorial Policy Checklist .

Statistics

For all statistical analyses, confirm that the following items are present in the figure legend, table legend, main text, or Methods section.

n/a	Confirmed	
☐	☒	The exact sample size (n) for each experimental group/condition, given as a discrete number and unit of measurement
☐	☒	A statement on whether measurements were taken from distinct samples or whether the same sample was measured repeatedly
☐	☒	The statistical test(s) used AND whether they are one- or two-sided<br>*Only common tests should be described solely by name; describe more complex techniques in the Methods section.*
☒	☐	A description of all covariates tested
☐	☒	A description of any assumptions or corrections, such as tests of normality and adjustment for multiple comparisons
☐	☒	A full description of the statistical parameters including central tendency (e.g. means) or other basic estimates (e.g. regression coefficient) AND variation (e.g. standard deviation) or associated estimates of uncertainty (e.g. confidence intervals)
☐	☒	For null hypothesis testing, the test statistic (e.g. F, t, r) with confidence intervals, effect sizes, degrees of freedom and P value noted<br>*Give P values as exact values whenever suitable.*
☒	☐	For Bayesian analysis, information on the choice of priors and Markov chain Monte Carlo settings
☒	☐	For hierarchical and complex designs, identification of the appropriate level for tests and full reporting of outcomes
☒	☐	Estimates of effect sizes (e.g. Cohen's d, Pearson's r), indicating how they were calculated

Our web collection on statistics for biologists contains articles on many of the points above.

Software and code

Policy information about availability of computer code

Data collection	All software used (including the version and parameter, when not default) is indicated in the Methods section.
Data analysis	All software used (including the version and parameter, when not default) is indicated in the Methods section.

For manuscripts utilizing custom algorithms or software that are central to the research but not yet described in published literature, software must be made available to editors/reviewers. We strongly encourage code deposition in a community repository (e.g. GitHub). See the Nature Research guidelines for submitting code & software for further information.

Data

Policy information about availability of data

All manuscripts must include a data availability statement. This statement should provide the following information, where applicable:

- Accession codes, unique identifiers, or web links for publicly available datasets
- A list of figures that have associated raw data
- A description of any restrictions on data availability

Data supporting the findings of this work are available within the paper and its Supplementary Information files. The datasets, plant materials, and mathematica programing language-written source code file (nb format file) are available from corresponding authors upon request. Accession code for genomic datasets is provided

Field-specific reporting

Please select the one below that is the best fit for your research. If you are not sure, read the appropriate sections before making your selection.

Life sciences study design

All studies must disclose on these points even when the disclosure is negative.

Sample size	Sample sizes were chosen based on prior experience and typical standards in the field. For statistical tests (i.e., Chi-Square, the Student's t-test, ANOVA test), enough sample size was ensured with 2-3 replicates each.
Data exclusions	Only experimental group and control group were included in the analyses. Any plants without these traits were excluded by genotyping.
Replication	All attempts at replication were successful.
Randomization	Random selection was not conducted. Plants in either experimental group or control group were included in the study. These two groups were distinct from each other; experimental group was compared with control group for phenotyping/expression.
Blinding	Random selection was not necessary since experimental group and control group were distinct from each other.

Reporting for specific materials, systems and methods

We require information from authors about some types of materials, experimental systems and methods used in many studies. Here, indicate whether each material, system or method listed is relevant to your study. If you are not sure if a list item applies to your research, read the appropriate section before selecting a response.

Materials & experimental systems

n/a	Involved in the study	
☐	☒	Antibodies
☒	☐	Eukaryotic cell lines
☒	☐	Palaeontology
☒	☐	Animals and other organisms
☒	☐	Human research participants
☒	☐	Clinical data

Methods

n/a	Involved in the study	
☐	☒	ChIP-seq
☒	☐	Flow cytometry
☒	☐	MRI-based neuroimaging

Antibodies

Antibodies used

H3K27me3 (ab6002; Abcam; 2 micro litter)
H3K4me3 (ab8580; Abcam; 1 micro litter)
H3 antibodies (ab1791; Abcam; 1 micro litter)
HA (12CA5; Roche; 5 micro litter)
HSP17.6 (ab80183) (1:1,000 diluted)
HSP21 (ab80175) (ab80175; Abcam)
HSP22 (Eurofins) (1:1,000 diluted)

Validation

The antibodies were validated by the suppliers.
https://www.abcam.co.jp/histone-h3-tri-methyl-k27-antibody-mabcam-6002-chip-grade-ab6002.html
https://www.abcam.co.jp/histone-h3-tri-methyl-k4-antibody-chip-grade-ab8580.html
https://www.abcam.co.jp/histone-h3-antibody-nuclear-marker-and-chip-grade-ab1791.html
https://www.sigmaaldrich.com/catalog/product/roche/roaha?lang=ja&region=JP
https://www.abcam.co.jp/hsp176-antibody-ab80183.html
https://www.abcam.co.jp/hsp21-antibody-ab80175.html

ChIP-seq

Data deposition

☒ Confirm that both raw and final processed data have been deposited in a public database such as GEO.

☒ Confirm that you have deposited or provided access to graph files (e.g. BED files) for the called peaks.

Data access links
May remain private before publication.

https://ddbj.nig.ac.jp/public/ddbj_database/dra/fastq/DRA011/DRA011879/

Files in database submission

Deposition at DDBJ
ChIP-seq (DRA011879)
H3K27me3 in WT control: 2 replicates

H3K27me3 in WT ACC: 2 replicates
H3K27me3 in jmjq control: 2 replicates
H3K27me3 in jmjq ACC: 2 replicates
H3K4me3 in WT control: 1 replicate
H3K4me3 in WT ACC: 1 replicate
H3K4me3 in jmjq control: 1 replicate
H3K4me3 in jmjq ACC: 1 replicate
H3 in WT control: 2 replicates
H3 in WT ACC: 2 replicates
H3 in jmjq control: 2 replicates
H3 in jmjq ACC: 2 replicates

Genome browser session
(e.g. UCSC)

Provide a link to an anonymized genome browser session for "Initial submission" and "Revised version" documents only, to enable peer review. Write "no longer applicable" for "Final submission" documents.

Methodology

Replicates

Replicates agreed well. Data was further confirmed by ChIP-qPCR

Sequencing depth

Experiment : Reads Mapped reads Length of reads Paired or single end
H3K27me3 in WT control:26407866 22939382 75 Single
H3K27me3 in WT ACC: 23619237 19981573 75 Single
H3K27me3 in jmjq control: 18719873 16146938 75 Single
H3K27me3 in jmjq ACC: 34665488 9888270 75 Single
H3K4me3 in WT control: 25941259 24713684 75 Single
H3K4me3 in WT ACC: 14541144 13663223 75 Single
H3K4me3 in jmjq control: 32172346 30598213 75 Single
H3K4me3 in jmjq ACC: 15888783 15201505 75 Single
H3 in WT control: 28364288 19007266 75 Single
H3 in WT ACC: 29298406 19913105 75 Single
H3 in jmjq control: 31263107 21604380 75 Single
H3 in jmjq ACC: 33354293 22467798 75 Single

Antibodies

H3K27me3 (ab6002; Abcam)
H3K4me3 (ab8580; Abcam)
H3 antibodies (ab1791; Abcam)
HA (12CA5; Roche)
Antibodies were validated by the supplier and by control ChIP reactions using plants lacking the antigene.

Peak calling parameters

Peak calling is described in the Methods.

Data quality

Peak attributes are described in the Methods.

Software

Software used is described in the Methods.
